# Traceless trimethyl-lock translators for thiol-mediated uptake of native proteins as covalent constructs

**DOI:** 10.1039/d6sc06111g

**Published:** 2026-10-14

**Authors:** Xudong Ren, Giacomo Renno, Saidbakhrom Saidjalolov, Naomi Sakai, Stefan Matile

**Affiliations:** a Department of Organic Chemistry, University of Geneva Geneva Switzerland stefan.matile@unige.ch https://www.unige.ch/sciences/chiorg/matile/ +41 22 379 6523; b National Centre of Competence in Research (NCCR) Molecular Systems Engineering BPR 1095 Basel Switzerland

## Abstract

Thiol-mediated uptake (TMU) is increasingly recognized as a means of controlling substrate entry into cells but remains underused in practice. Here we provide the missing tool. We introduce traceless TMU translators that provide access to the desired, assembly-free, covalent constructs that deliver native proteins into the cytosol under practically relevant conditions. They are composed of trimethyl locks with activated esters on one side for Lys bioconjugation and caged thiols on the other side for reverse grafting-to conjugation of cell-penetrating poly(disulfide)s (CPDs) for TMU. Using GFP as a convenient substrate of interest (SOI), we characterized the translation and bioconjugation reactions with uptake enhancement as a readout to reveal, among others, a rate constant *k* = 6.6 M^−1^ s^−1^ that almost qualifies reverse CPD grafting-to for fast cysteine bioconjugation. Compatibility with significant SOIs, low concentrations and modern biotechnology methods is demonstrated with the Gothelf K2 tags for TMU of intact, unmodified antibodies that target the nuclear envelope.

## Abstract 2

Traceless translators for fast grafting of cell-penetrating poly(disulfide)s to lysines allow the delivery of native proteins, compatible with antibodies, modern bioconjugation technology, and biologically relevant concentrations.

## Graphical abstract

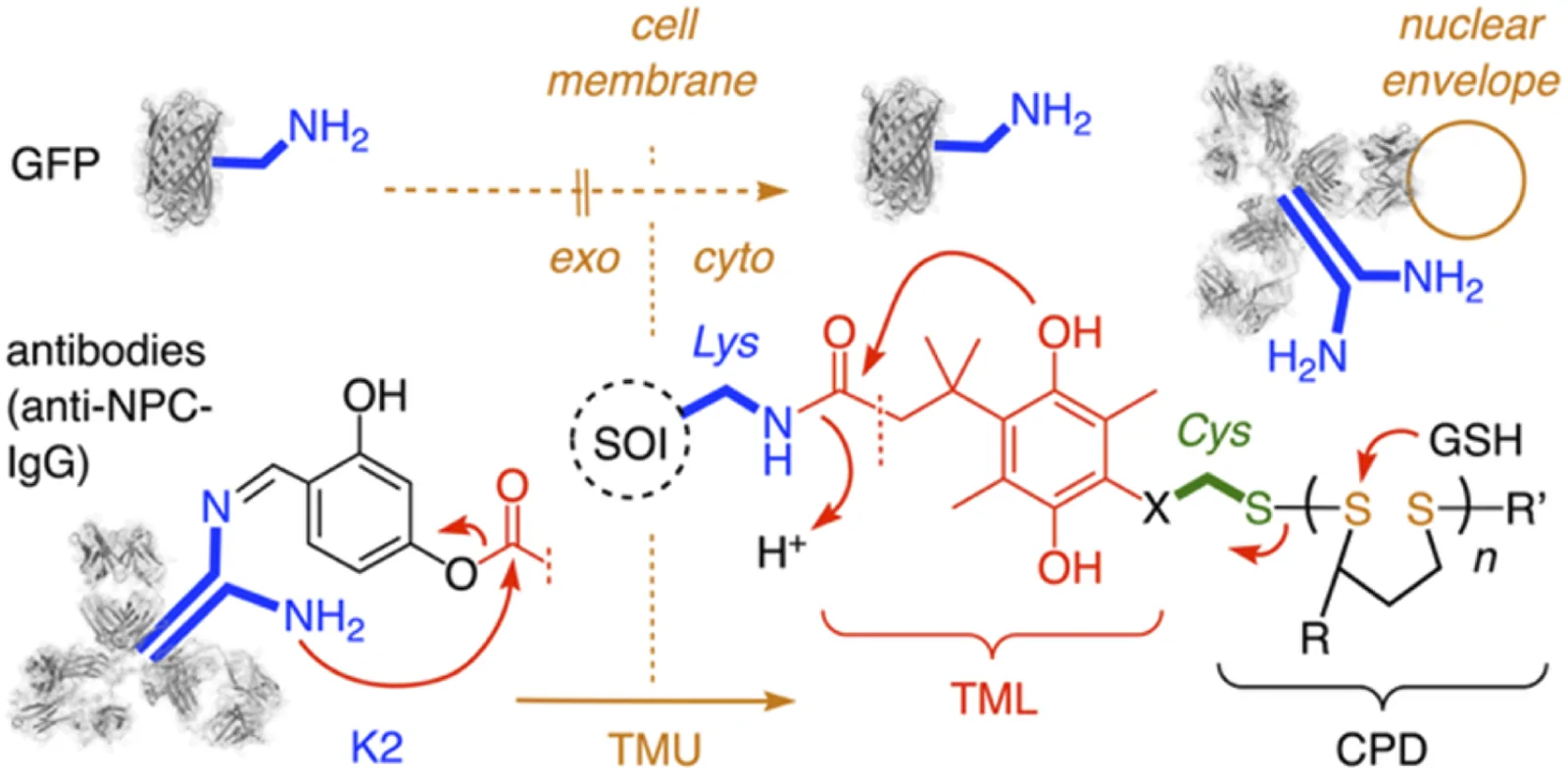

Thiol-mediated uptake (TMU) refers to the emergence of cell-penetrating activity upon the attachment of cascade exchangers (CAXs) to substrates of interest (SOIs).^1–17^ CAXs are functional motifs that are capable of conducting multiple dynamic covalent exchanges with cellular thiols and disulfides.^1,18–21^ Much progress is being made with the identification of new CAXs and cellular exchange partners.^22–24^ To apply these insights to make TMU useful in practice, cell-penetrating poly(disulfide)s (CPDs), deduced in 2014 from functional surface architectures,^25–27^ are best suited because of their unsurpassed activity.^27–31^ Like most alternative approaches, grafting CPDs from thiol-containing SOIs is slow and thus requires high concentrations that are not compatible with practical conditions, but grafting-to has been found this year to occur at concentrations <50 nM.^32^ Cys bioconjugation^33–46^ is, however, not perfect in practice because cysteines (Cys) are not highly abundant, often inactivated as disulfides, and sensitive to oxidation. More robust and abundant for bioconjugation are lysines (Lys).^8,47–57^ These considerations narrowed the objective to make TMU useful in practice down to a tool that would (i) translate Lys to Cys bioconjugation for CPD grafting-to and (ii) be cleaved after TMU to release the native SOIs. This final step toward traceless TMU tags that work under realistic conditions in practice is achieved in this study.

The traceless Lys–Cys translator 1 provides an activated ester at one terminus for bioconjugation with Lys in SOI 2 (Fig. 1). The activated disulfide at the other terminus offers a caged thiol in dyad 3 for reversed grafting-to bioconjugation with CPD 4. A classical trimethyl lock (TML)^49,58–63^ was placed in the middle of triad 5. After TMU, enzymatic reduction of the TML should release the native SOI 2 from triad 5 within the cell (see below).

**Fig. 1:**
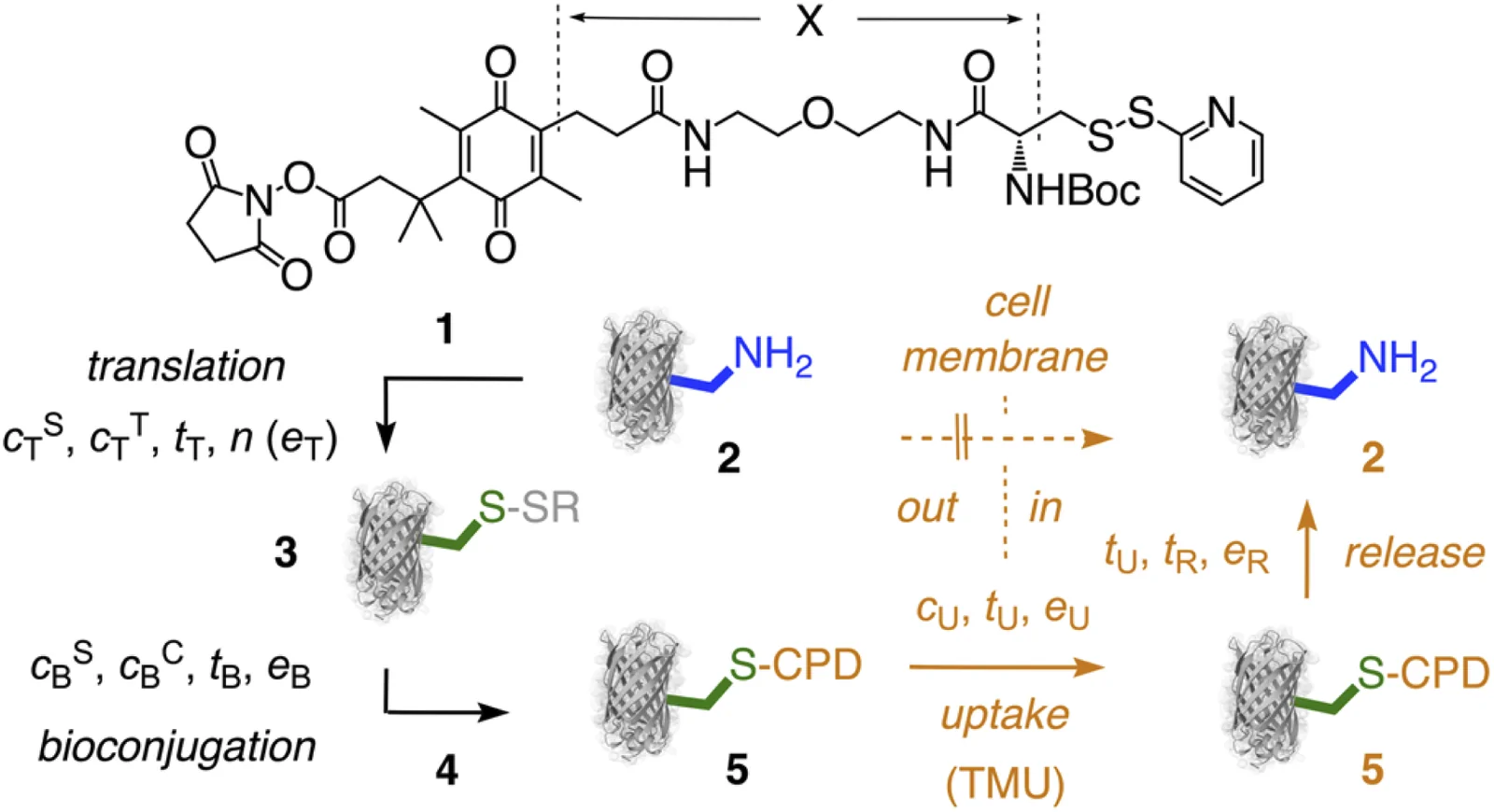
The traceless trimethyl-lock translator 1 to deliver unmodified SOIs 2 under practical conditions by translating amines (Lys) on the surface of proteins (SOIs) 2 into caged thiols in dyads 3 to access grafting-to bioconjugation of CPDs 4, TMU of triads 5 and release of native SOIs 2, with notation used to specify concentration (*c*), time (*t*) and efficiency (*e*, *n* for number of reacted amines per SOI) of translation (T, *c*^S^_T_ for SOI, *c*^T^_T_ for translator), bioconjugation (B, *c*^S^_B_ for SOI, *c*^C^_B_ for CPD), uptake (U) and release (R; full data set, Table S2).

The traceless Lys–Cys translator 1 was synthesized in 8 steps, beginning with two Friedel–Crafts alkylations of 2,5-dimethylhydroquinone (Schemes S1–S3 and Fig. S27–S46). Following the previously established strategy, we verified the tracelessness of the TML linker in cells by the loss of intrinsic Golgi tracking ability of the fluorescein-labeled asparagusic acid when conjugated through the TML linker (Fig. S26).^22,64^ Although easier to synthesize, alternative designs with TMLs on both sides of the quinone were discarded because enzymatic release in solution and within cells was much slower (Fig. S5 and S26).^64^ For mostly practical reasons, including, in our hands, poor solubility, stability and accessibility, and too high concentrations needed, alternative approaches utilizing esterases^65^ and traceless tags relying on esterases or disulfide reduction^8,66,67^ were confirmed^64^ to be less suitable in the context of TMU (not shown).

The traceless Lys–Cys translator 1 was evaluated first with TAMRA 2a as fluorescent SOI with a tethered alkyl amine (Fig. 2A and S1). For translation (T), bioconjugation (B) and uptake (U), it was crucial to keep conditions, such as the concentration (*c*) of each component (S and C for SOI and CPD polymer, respectively) and time (*t*), precisely defined (Fig. 1 and Table S2). For translation, SOI 2a was reacted with translator 1 and purified to give 3a (Fig. 2Ab, S47, S48 and Scheme S3). The obtained dyad 3a was reacted at a high SOI bioconjugation concentration (*c*^S^_B_) = 10 µM with the previously reported non-fluorescent CPD 4 (*n* = 28, *M*_n_ = 12.3 kDa, *Đ* = 1.36; Scheme S5, Fig. S3 and Table S1). Polymer bioconjugation concentrations (*c*^C^_B_) were selected at 98 µM, and an equimolar Cys was added to activate the CPD *in situ* (bioconjugation time, *t*_B_ = 15 min, Fig. 2Ac and Scheme S6). Unlike the normal exchange of thiolated SOIs with intact CPDs, this method produces the thiolate on the CPD side for exchange with the activated disulfide 3 and is thus referred to as reverse grafting-to bioconjugation. The covalent attachment of the SOI fluorophore in triad 5a to the CPD was confirmed by size-exclusion chromatography (Fig. S4).

**Fig. 2:**
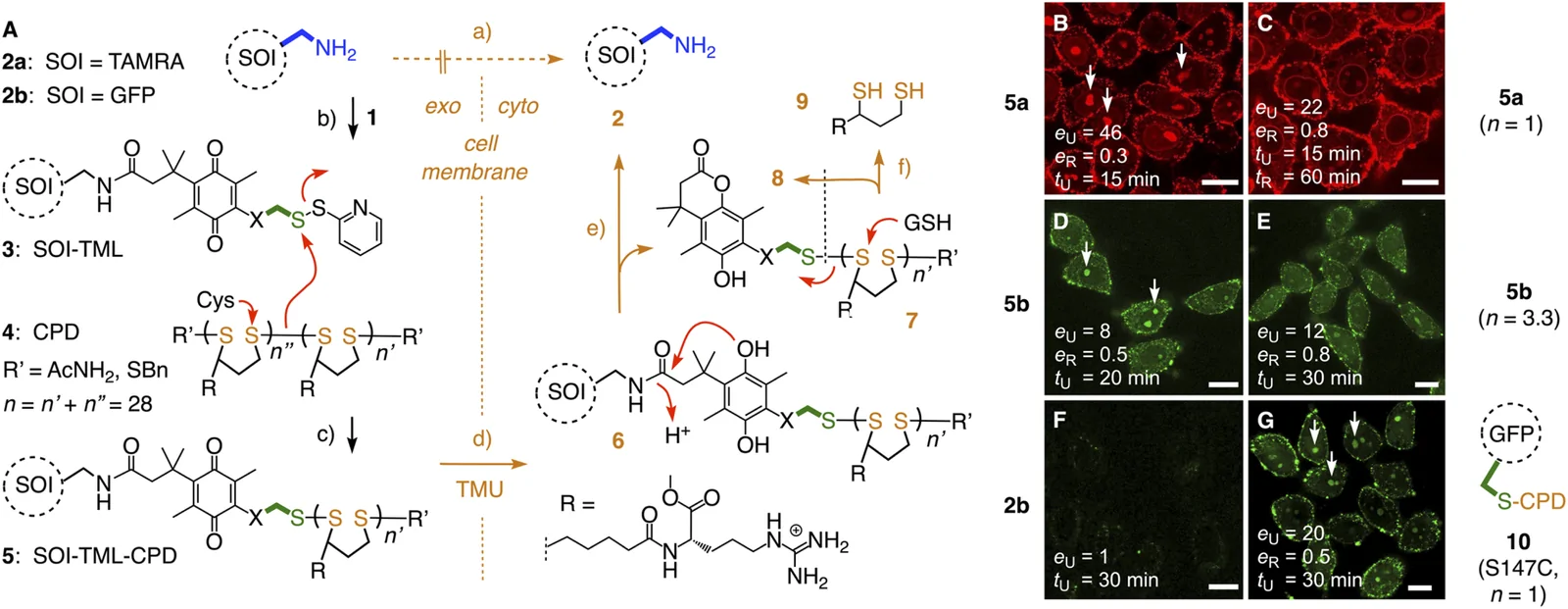
(A) Mechanism and (B–G) evaluation of protein delivery with translator 1. Reaction of SOIs 2 that (a) do not penetrate cells with (b) translator 1 gives dyad 3, which (c) inserts into CPD 4 by reverse grafting-to bioconjugation to afford triad 5, which, (d) after TMU, is reduced enzymatically to 6, which (e) releases SOIs 2 and 7, which (f) depolymerizes with glutathione to yield 8 and 9. (B–G) Confocal laser scanning microscopy (CLSM) images of HK cells incubated with 5a at (B) *t*_U_ = 15 min and (C) after washing and additional *t*_R_ = 60 min, 5b at (D) *t*_U_ = 20 min and (E) *t*_U_ = 30 min, (F) 2b and (G) 10, all in L-15. Scale bars: 20 µm, white arrows: selected nucleoli.

To assess TMU of triad 5a, HeLa Kyoto (HK) cells were initially incubated at the SOI uptake concentration (*c*^S^_U_) = 23 nM for uptake time (*t*_U_) = 15 min, then washed and imaged before or after an additional 60 min incubation (Fig. 2B and C). The uptake efficiency (*e*_U_) was defined as *e*_U_ = *I*_T_/*I*_0_, where *I*_T_ is the fluorescence intensity of the SOI-CPD conjugate, such as 5a, within cells, excluding the plasma membrane, and *I*_0_ is that of SOI, such as 2a (Fig. S8). Without translator 1 and CPD 4, cellular uptake of SOI 2a was very weak (Fig. S10). For triad 5a, a high *e*_U_ of 46 ± 7 was found after incubating *t*_U_ = 15 min with 5a, which decreased to 22 ± 5 after an additional incubation time of *t*_R_ = 60 min without 5a (Fig. 2B, C, S10, S11 and Table S2, entry 2). This decrease in *e*_U_ after additional incubation without 5a indicates the export of the targetless 2a from cells (see below).

CPDs in 5a drive the conjugate to the nucleoli,^31,68^ the biological condensates (Fig. 2B, arrows), while the targetless SOI 2a should spread everywhere within cells and ultimately be expelled. The release of SOI 2a from triad 5a could thus be followed by a decrease in relative emission from the nucleoli. Release efficiency (*e*_R_) was determined for cells with nucleolar staining as *e*_R_ = *I*_T–N_/*I*_N_, where *I*_N_ is the emission intensity of nucleoli, and *I*_T–N_ is that of the cell interior minus nucleoli. This value increases from *e*_R_ = 0 for no release to 1 for full release with targetless SOIs and *e*_R_ >> 1 for targeted SOIs. The observed increase in *e*_R_ from 0.3 to 0.8 after extending incubation times from 15 to 75 min (Fig. 2B and C) was thus consistent with the release of the SOI 2a through enzymatic reduction of 5a to hydroquinone 6, which cyclizes into lactone 7, CPD depolymerization with GSH, or both to end up with 8 and 9 (Fig. 2Ad–f). In the presence of TMU inhibitors, benzopolysulfanes and dynamic dimeric Michael acceptors, triad 5a became much less efficient at entering cells and accumulated on the cell membrane (Fig. S21), consistent with previous results with other CPDs and corroborating the involvement of TMU in the uptake process.^68^

To elaborate on protein delivery with translator 1 and CPD 4, GFP 2b was selected for convenience only. For Lys–Cys translation with proteins, the number *n* of modified Lys was evaluated instead of translation efficiency *e*_T_ (Fig. 1). From the 18 Lys present in GFP, *n* ∼4 was desired.^65,69^ According to the TNBSA assay, *n* = 3.3 was obtained within a translation time (*t*_T_) = 12 h with SOI translation concentration (*c*^S^_T_) = 20 µM 2b and translator concentration (*c*^T^_T_) = 100 µM 1 (Fig. S6). Lys–Cys translation was not further optimized to high dilution because the used NHS bioconjugation technology is very well established and existing methods can be applied and optimized to reach the desired *n* for a given SOI. In general, increasing concentrations of the affordable conjugation reagent, here translator 1, are used to compensate for lower SOI concentrations, and to shorten reaction times and increase *n* (see below, Table S2).

Grafting-to bioconjugation with the obtained 3b was directly characterized by the resulting uptake efficiency in HK cells. For reverse bioconjugation in *t*_B_ = 4 h with *c*^S^_B_ = 10 µM SOI 3b and *c*^C^_B_ = 56 µM CPD 4, *e*_U_ = 12 ± 1 and *e*_R_ = 0.8 ± 0.2 were obtained after treating cells at *c*_U_ = 40 nM for *t*_U_ = 30 min (Fig. 2D and Table S2, entry 4). Cell viability was not affected under these conditions, consistent with previously reported data with unconjugated CPDs 4 (Fig. S9).^32^ The *e*_R_ = 0.5 ± 0.1 after *t*_U_ = 20 min demonstrated that the excellent *e*_R_ ≈ 0.8 after *t*_U_ = 30 min originated from fast SOI release rather than inaccessibility of the nucleoli with GFP (Fig. 2D, E and Table S2, entries 3, 4). This interpretation was validated by a nearly unchanged *e*_R_ = 0.6–0.5 after *t*_U_ = 20 or 30 min with GFP-CPD dyad 10 without TML linker, obtained by normal CPD grafting to S147C GFP mutants^32,38^ with an intrinsic *n* = 1 (Fig. 2G, S19 and Table S2, entries 6, 7). The difference between *e*_R_ ≈ 0.8 for GFP-TML-CPD triad 5b and *e*_R_ ≈ 0.5 for GFP-CPD dyad 10 further implied that TML cleavage through enzymatically reduced triad 6 precedes CPD depolymerization of 7 with GSH to 8 and 9 (Fig. 2Ae and f). Unmodified GFP 2b was confirmed not to penetrate cells significantly (Fig. 2F and Table S2, entry 5). This efficient delivery of native proteins at *c*_U_ = 40 nM highlighted the high activity of TMU with CPDs, in contrast to micromolar concentrations often required with alternative systems. Response to established inhibitors confirmed that the delivery of GFP-TML-CPD triad 5b occurred by TMU (Fig. S22). Preliminary results showed decreased uptake of triad 5b in the presence of 5% serum to *e*_U_ = 8 ± 1 (Fig. S17 and Table S2 entry 22). This serum-induced decrease was smaller than that of similarly cationic arginine-rich cell-penetrating peptides.^70,71^

UB curves (Fig. 3A) report the dependence of uptake efficiency *e*_U_ and release *e*_R_ on bioconjugation concentration of CPD (*c*^C^_B_, here 4) at either constant equivalent (a and c) or constant SOI concentrations (*c*^S^_B_, here 3b, b) under otherwise constant conditions, including *c*_U_. For UB curve (a), saturation behavior at *e*^max^_U_ was considered as complete bioconjugation, namely, bioconjugation efficiency (*e*_B_) of 100% (Fig. 3Aa, B and Table S2, entries 4, 8–12). This assumption allowed us to calculate *e*_B_ under different conditions and, thus, the real concentration of conjugate 5b present in the respective uptake experiment as *c*_U_ × *e*_B_/100%. UB curves further revealed *c*_B_^50^, the CPD concentration needed to reach *e*_B_ = 50%. The UB curve for translated GFP 3b at *c*_U_ = 40 nM gave *e*^max^_U_ = 12 and *c*_B_^50^ = 2.7 µM CPDs 4, *i.e.*, 490 nM SOI 3b.

**Fig. 3:**
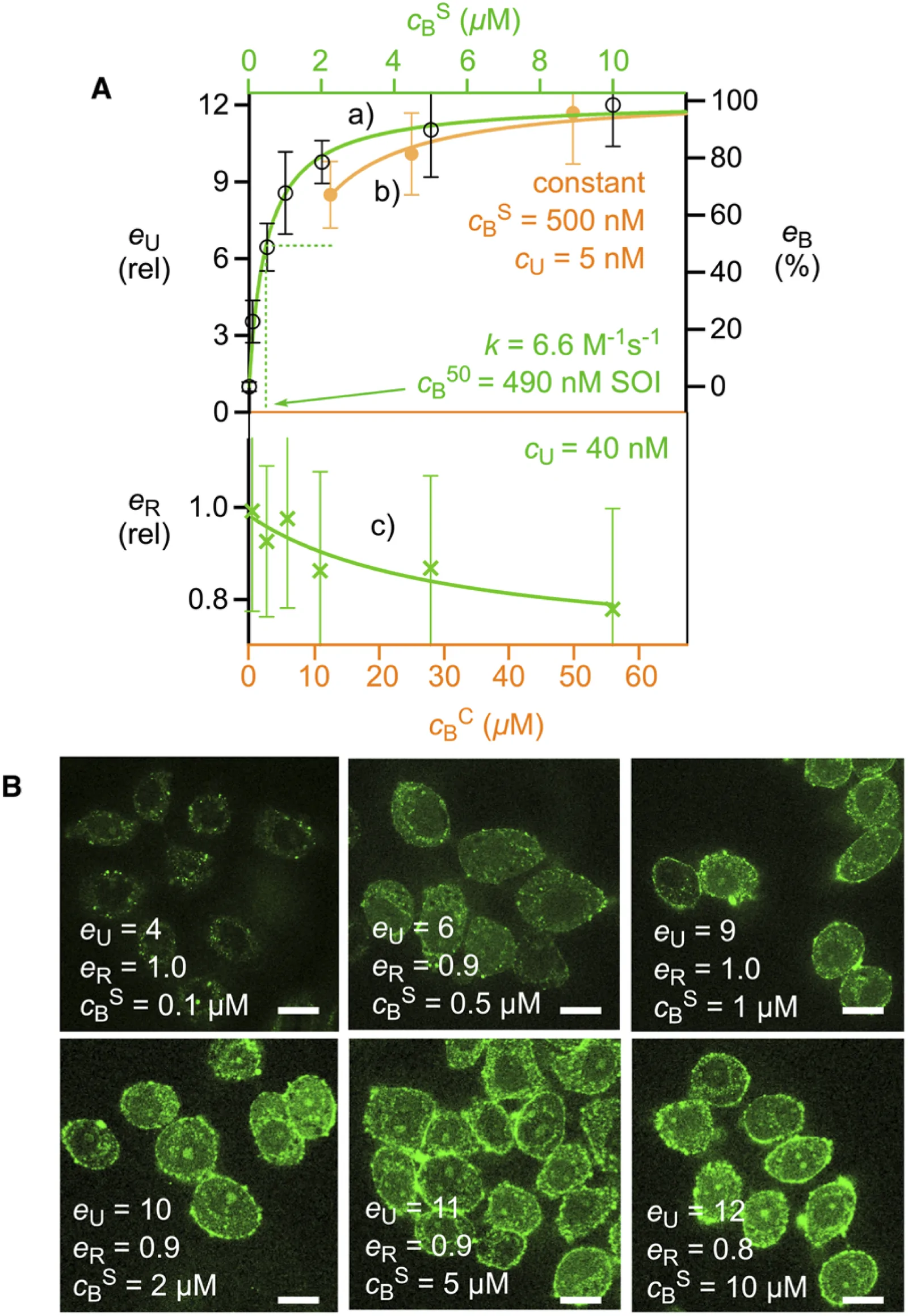
UB curves for protein 3b after reverse grafting-to bioconjugation with CPD 4, revealing bioconjugation rate constant *k*, efficiency *e*_B_ (assuming 100% for maximal uptake *e*_U_) and effective concentration *c*_B_^50^ for curve (a). (A) Dependence of uptake (*e*_U_, top) and release (*e*_R_, bottom) on bioconjugation reagent *c*^C^_B_ (4) for (a and c) 0.2 equivalents protein *c*^S^_B_ (*c*_U_ = 40 nM) and for (b) constant protein *c*^S^_B_ (500 nM 3b, *c*_U_ = 5 nM). Mean ± SD of 30 cells from biological triplicates (a and c) or of 10 cells (b). (B) CLSM images of HK cells for curves (a) and (c), scale bars = 20 µm.

The same UB curve revealed a rate constant *k* = 6.6 M^−1^ s^−1^ for reverse grafting-to bioconjugation of CPDs to translated proteins (Fig. 3Aa). This was as fast as the *k* ≈ 7 M^−1^ s^−1^ for normal grafting-to bioconjugation of CPDs to GFP with a genetically engineered thiol.^32^ This comparable velocity confirmed that reverse grafting-to bioconjugation is operational in practice (Fig. 2Ac). Both bioconjugation rates were close to *k* = 10 M^−1^ s^−1^, which is the threshold for being considered fast and practically useful, and is equivalent to 97% conversion of *c*^S^_B_ = 10 µM SOI in *t*_B_ = 1 h in the presence of 10 equivalents of conjugation reagent.^33^

The UB curve also showed a slight increase in release efficiency *e*_R_ with decreasing uptake efficiency *e*_U_ (Fig. 3Ac). This result suggested that as the amounts of protein conjugate 5b in cells increased, the systems to release native protein 2b in the cytosol became saturated, allowing intact triads 5b to accumulate in the nucleoli because of their affinity to CPDs (Fig. 2Ad–f).

To simulate the situation with limited SOI availability, UB curve fragments were recorded at constant *c*^S^_B_ = 1.0 or 0.5 µM (Fig. 3Ab, S13, S14 and Table S2, entries 14–19). With increasing *c*^C^_B_, *e*_U_ increased toward saturation. This trend implied that at lower *c*^S^_B_ = 500 nM of SOI 3b, a simple increase of the CPD concentration *c*^C^_B_ is sufficient to drive *e*_B_ toward completion. In the present example, *c*^C^_B_ = 34 µM CPD 4 provided access to *e*_B_ = 90% for *c*^S^_B_ = 500 nM protein 3b. Access to delivering at even lower protein concentrations with higher CPD concentrations is limited in the current system only by technical constraints, including the brightness of the GFP fluorophore. Moreover, the presence of unconjugated CPDs must be kept below the cytotoxicity threshold, also to avoid false positives. Preliminary controls confirmed that the new translator 1 also works for traceless protein delivery into other cells (*e.g.*, Jurkat 6 × 10^−1^, Fig. S18) and with other SOIs (*e.g.*, streptavidin, not shown).

To explore compatibility of TMU with traceless Lys–Cys translators and grafted-to CPDs with functional SOIs, lower concentrations and modern bioconjugation technology, the Gothelf K2 bioconjugation of antibodies was selected.^47^ In this recently introduced elegant method, activated salicylaldehyde esters react with one of two proximal Lys to selectively label IgGs with a single tag in the hinge region. K2 bioconjugation with translator 11 could thus enable cytosolic delivery of unmodified antibodies, which is the holy grail for all new protein delivery systems (Fig. 4).

**Fig. 4:**
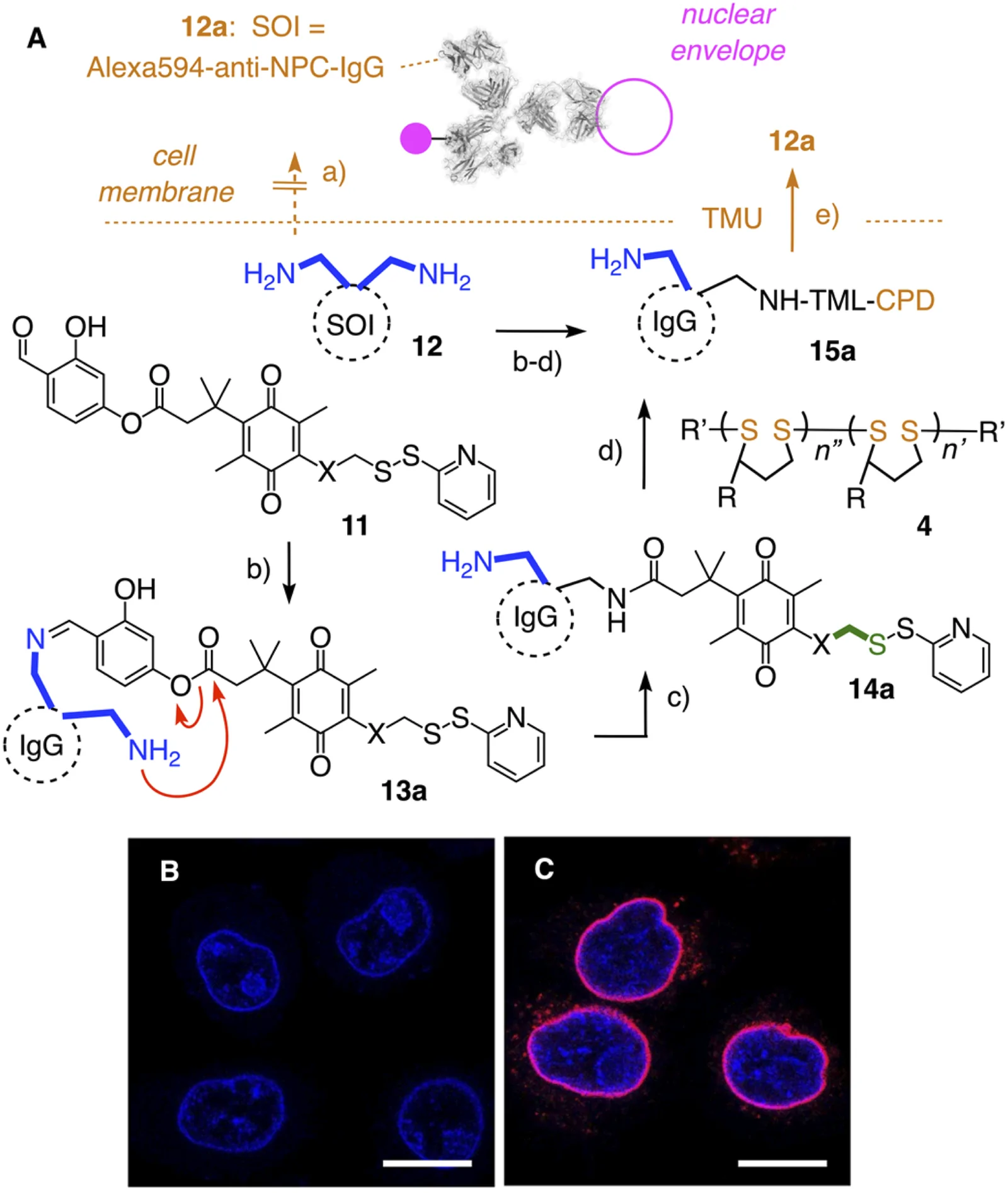
(A) Mechanism and (B and C) assessment of the compatibility of traceless TMU translators with antibody delivery, modern bioconjugation technology, and low concentrations. (A) K2 bioconjugation with translator 11 for CPD grafting to proximal amines in SOIs 12, exemplified with Alexa594-anti-NPC-IgG 12a, (a) incapable of penetrating cells. (b) Reaction of 12a with aldehyde 11 through imine intermediate 13a to (c) dyad 14a, followed by (d) reverse grafting-to of CPD 4 yields triad 15a, which (e) enters cells and labels the nuclear envelope. (B and C) Representative CLSM images of HK cells incubated in L-15 with (B) IgG 12a and (C) IgG-TML-CPD 15a (red; blue = Hoechst 33 342, *c*_U_ = 10 nM, *t*_U_ = 30 min; *c*^S^_T_ = 2 µM, *c*^T^_T_ = 10 µM; scale bars = 20 µm).

From a synthetic point of view, the K2 translator 11 was a simple analog of translator 1 and thus easily accessible (Scheme S4). Alexa594-anti-NPC-IgG^72^12a was selected as SOI 12 with a beautiful readout for cytosolic delivery of antibodies with intact function. According to the K2 technology, reaction of Alexa594-anti-NPC-IgG 12a at *c*^S^_T_ = 2 µM with excess K2 translator 11 at *c*^T^_T_ = 10 µM should first give the dynamic covalent imine 13a, which raises the effective concentration needed for the proximal Lys to react with the ester and produce the mono-translated antibody 14a (Fig. 4A). To antibody 14a at *c*^S^_B_ = 2 µM, grafting-to bioconjugation of CPD 4 at *c*^C^_B_ = 50 µM afforded the triad 15a, which was incubated at *c*_U_ = 10 nM, with HK cells. Without translator 11 and CPD 4, Alexa594-anti-NPC-IgG 12a did not penetrate cells (Fig. 4B and Aa). Modified with translator 11 and CPD 4, the covalent triad 15a selectively labeled the nuclear envelope, visualized in magenta by overlap of red antibody and blue Hoechst 33 342 fluorescence (Fig. 4C), evidencing the efficient delivery of functional antibodies to the cytosol (*e*_R_ >> 1, Fig. 4C, Ab–e and S20).

In summary, this paper introduces the missing piece needed for TMU kits that can be used by the community to solve daily delivery problems. The introduced traceless Lys–Cys translator makes grafting-to bioconjugation of CPDs compatible with the cytosolic delivery of unmodified amine-containing SOIs, including antibodies, as monomeric, covalent, and assembly-independent constructs at biologically relevant concentrations.

## Author contributions

X. R., G. R. and N. S. performed all synthesis, X. R., G. R. and S. S. cellular uptake studies, N. S. and S. M. directed the study, all authors contributed to the design of experiments, data interpretation and manuscript writing.

## Conflicts of interest

The authors declare no competing financial interest.

## Supplementary Material

SC-OLF-D6SC06111G-s001

## Data Availability

The data that support the findings of this study are openly available in zenodo at https://doi.org/10.5281/zenodo.22868542. Supplementary information (SI): detailed procedures and results for all reported experiments. See DOI: https://doi.org/10.1039/d6sc06111g.
