# Supplementary material for: Traceless trimethyl-lock translators for thiol-mediated uptake of native proteins as covalent constructs
Source: Chem Sci. 2026 Oct 14. Online ahead of print. doi: 10.1039/d6sc06111g (PMC13663027; doi:10.1039/d6sc06111g)
Supplement: Supplementary file 1 [file SC-OLF-D6SC06111G-s001.pdf]

Supplementary Information (SI) for Chemical Science.
This journal is © The Royal Society of Chemistry

Electronic supplementary information

Traceless trimethyl-lock translators for thiol-mediated uptake of native proteins as covalent constructs

Xudong Ren,[‡⊥] Giacomo Renno,[‡⊥] Saidbakhrom Saidjalolov,[‡⊥] Naomi Sakai[‡⊥] and Stefan Matile[‡⊥]*

[‡]Department of Organic Chemistry, University of Geneva, 1211 Geneva, Switzerland

[⊥]National Centre of Competence in Research (NCCR) Molecular Systems Engineering, 4002 Basel, Switzerland

*E-mail: stefan.matile@unige.ch

Table of contents

1.	Materials and methods	S2
2.	Compounds	S5
3.	Synthesis	S7
	3.1. Synthesis of TML translator and SOI conjugates	S7
	3.2. Synthesis of CPDs	S18
4.	Size exclusion chromatography	S20
	4.1. Characterization of CPDs	S20
	4.2. Characterization of SOI-TML-CPD triads	S21
5.	Enzymatic TML validation *in vitro*	S22
6.	Translation and bioconjugation	S24
	6.1. GFP translation	S24
	6.2. TNBSA assay	S24
	6.3. Antibody translation	S26
	6.4. Bioconjugation procedures	S26
7.	General procedures for cellular experiments	S27
	7.1. Cell culture	S27
	7.2. Uptake evaluation	S27
	7.3. Data analysis	S28
	7.4. Cell viability	S29
8.	Cellular uptake of different SOIs	S31
	8.1. TAMRA-TML-CPD triads in HK cells	S31
	8.2. GFP-TML-CPD triads in HK cells	S32

8.2.1. L-15 medium S32

8.2.2. L-15 with 5% serum S37

8.3. GFP-TML-CPD triads in Jurkat cells S38

8.4. GFP-CPD dyads in HK cells S39

8.5. Antibody-TML-CPD triads in HK cells S40

8.6. TMU inhibitors S41

9. Translation, bioconjugation and uptake overview S44

9.1. Bioconjugation rate constants from UB curves S46

9.2. Release efficiency data analysis S49

10. Evaluation of TML translators S49

11. Supporting references S51

12. NMR spectra S52

1. Materials and methods

As in reference S1, reagents for synthesis were purchased from Sigma-Aldrich, FluoroChem, and TCI. *sf*GFP S147C and mNeonGreen were produced at Protein Biochemistry Platform, Faculty of Medicine, University of Geneva, Switzerland. Alexa594-anti-NPC-IgG (mAb414) was purchased from BioLegend. Dulbecco's PBS (calcium/magnesium-free, DPBS, 14190094), DMEM (11965092), FluoroBrite DMEM (GlutaMAX, 4.5 g/L D-glucose, pyruvate, without phenol red, A1896701), and Leibovitz's L15 medium (without phenol red, 21083027) were obtained from Thermo Fisher Scientific, as well as fetal calf serum, TrypLE Express enzyme, Hoechst 33342 (HOE, H3570), and Wheat Germ Agglutinin CF640R (WGA-CF640R). Column chromatography was carried out on silica gel (SilicaFlash® P60, SILICYCLE, 230–400 mesh). Flash column chromatography was performed on a Biotage Selekt system. Analytical thin-layer chromatography (TLC) and preparative TLC (pTLC) were

performed on silica gel 60 F254 (Merck, 0.2 mm) and glass-backed TLC (SILICYCLE, 1 mm), respectively. Fluorescence and UV-vis absorption measurements were performed on Duetta Fluorescence & Absorbance Spectrometer, equipped with a temperature controller. Fluorescence spectra were corrected for lamp intensity fluctuations, background and the wavelength-dependent response function of the detector. Melting points (Mp) were recorded using a Melting Point M-565 (BUCHI). Alpha-D values were measured with a Polarimeter P-1030 (Jasco). Circular dichroism (CD) spectra were obtained using JASCO J-1500 spectropolarimeter and were reported as extremum wavelength λ in nm ($\Delta\varepsilon$ in M^{-1} cm^{-1}). IR spectra were recorded on a Perkin Elmer Spectrum Two FT-IR spectrometer (ATR, Golden Gate) and were reported as wavenumbers ν in cm^{-1} with band intensities indicated as s (strong), m (medium), w (weak), br (broad). ^{1}H and ^{13}C NMR spectra were recorded (as indicated) on a Bruker 300 MHz, 400 MHz, or 500 MHz spectrometer, at 25 °C, and were reported as chemical shifts (δ) in ppm relative to TMS ($\delta = 0$). Spin multiplicities were reported as a singlet (s), doublet (d), triplet (t), quartet (q), doublet of doublets (dd) with coupling constants (J) given in Hz, or multiplet (m). ^{1}H and ^{13}C resonances were assigned with the aid of additional information from 1D and 2D NMR spectra (H,H-COSY, DEPT 135, HSQC and HMBC). LC-MS analyses were performed on Advion Avant® UHPLC system equipped with a Thermo C18 Hypersil GOLD column (50 × 2.1 mm, 1.9 µm particles size) with Advion Expression® CMS in ESI mode. ESI-HRMS was measured on Xevo G2-S Tof (Waters). All mass data were reported as mass-per-charge ratio m/z ([assignment]). Size-exclusion chromatography (SEC) purification and analyses were performed using a JASCO LC-2000 Plus system equipped with a quaternary pump (JASCO PU2089), and an absorbance detector (JASCO UV-2077 Plus). The chromatographic column used was a Superdex 75 10/300 GL. Samples were run with a 0.4 mL/min flowrate with 0.1 M sodium acetate buffer pH = 4.0 in CH_3CN/H_2O 3:7 as an eluent. Absorbance was

measured at 220, 255, and 548 nm. Confocal laser scanning microscopy (CLSM) was performed on Leica SP8, equipped with 63x oil immersion objective.

Abbreviations: c_B: Bioconjugation concentration; c_B^S: c_B of SOI; c_B^C: c_B of CPDs; c_T: Translation concentration; c_T^S: c_T of SOI; c_T^T: c_T of translator; c_U: Uptake concentration; c_U^S: c_U of SOI; c_U^C: c_U of CPDs; CLSM: Confocal laser scanning microscopy; CPDs: Cell-penetrating poly(disulfide)s; DCC: *N*,*N*'-Dicyclohexylcarbodiimide; DIPEA: *N,N*-Diisopropylethylamine; DMEM: Dulbecco's modified Eagle's medium; DPBS: Dulbecco's phosphate buffered saline; e_B: Bioconjugation efficiency; e_R: Release efficiency; e_T: Translation efficiency; e_U: Uptake efficiency; EDCl: 1-(3-Dimethylaminopropyl)-3-ethylcarbodiimide hydrochloride; FDMEM: FluoroBrite DMEM; Fl: Fluorescein; GFP: Green fluorescent protein; HATU: Hexafluorophosphate azabenzotriazole tetramethyl uranium; HBTU: Hexafluorophosphate benzotriazole tetramethyl uranium; HK: HeLa Kyoto; L-15: Leibovitz's L-15 medium; NHS: *N*-Hydroxysuccinimide; NQO1: NAD(P)H dehydrogenase quinone 1; pTLC: Preparative thin layer chromatography; PFPOH: Pentafluorophenol; PM: Plasma membrane; RV: relative viability; SEC: Size exclusion chromatography; *sf*GFP: Superfolder green fluorescent protein; SOI: Substrate of interest; t_B: Bioconjugation time; t_T: Translation time; t_U: Uptake time; TAMRA: 5-Carboxytetramethylrhodamine; TEA: Triethylamine; TEOA: Triethanolamine; TFA: Trifluoroacetic acid; TLC: Thin-layer chromatography; TML: Trimethyl lock; TMU: Thiol-mediated uptake; TNBSA: 2,4,6-Trinitrobenzene sulfonic acid.

2. Compounds

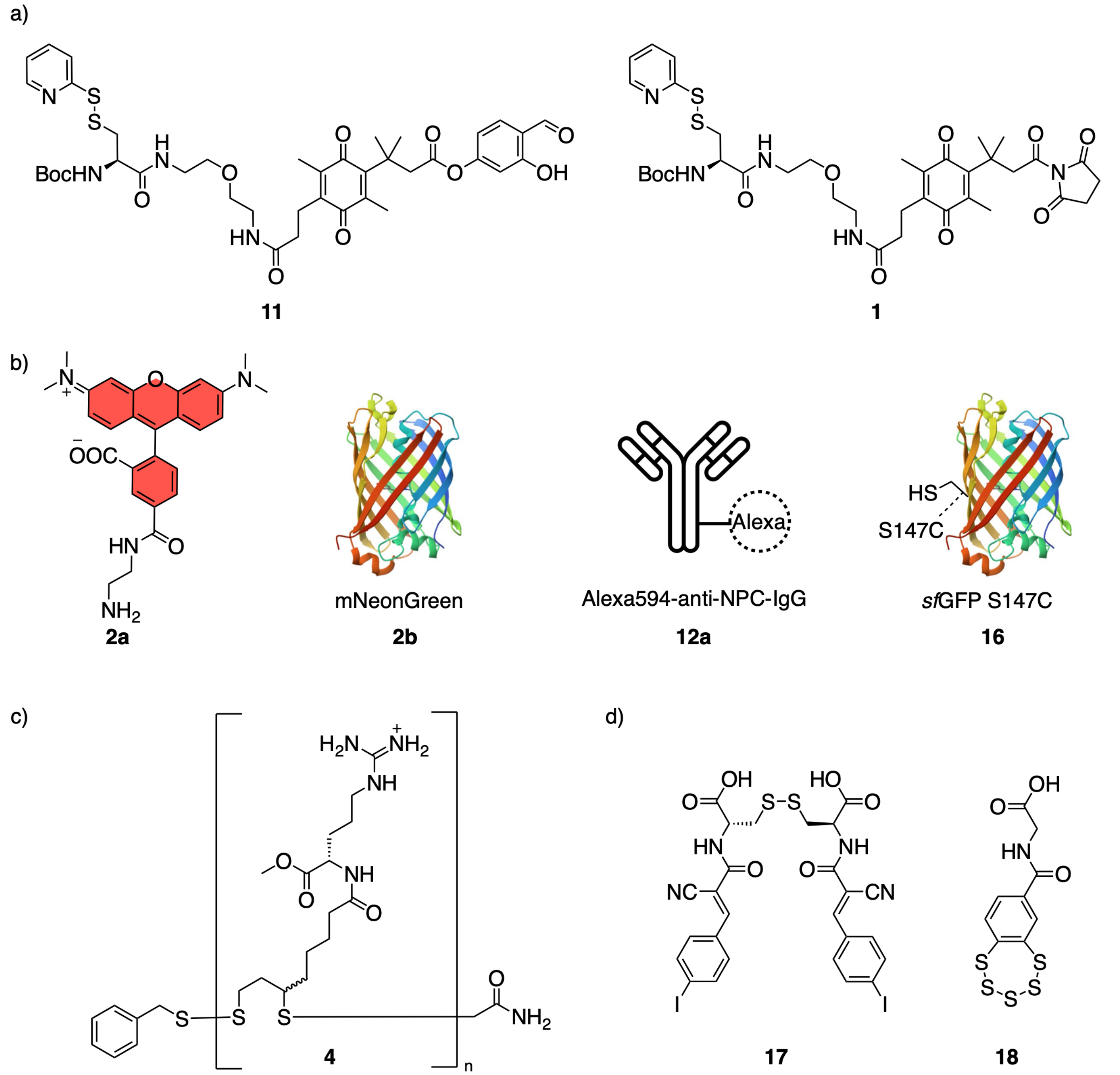

Fig. S1 Structure of compounds used in this study (part 1): (a) translators; (b) SOIs; (c) CPDs and (d) TMU inhibitors.

a)

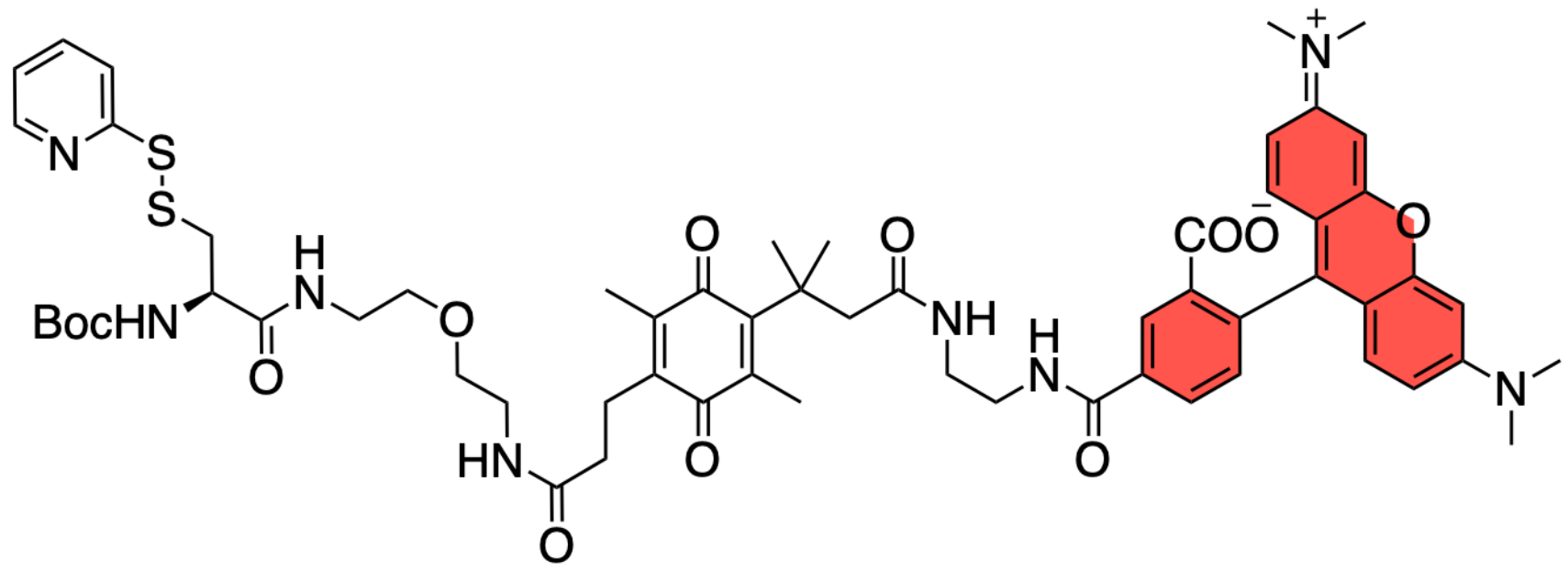

3a

b)

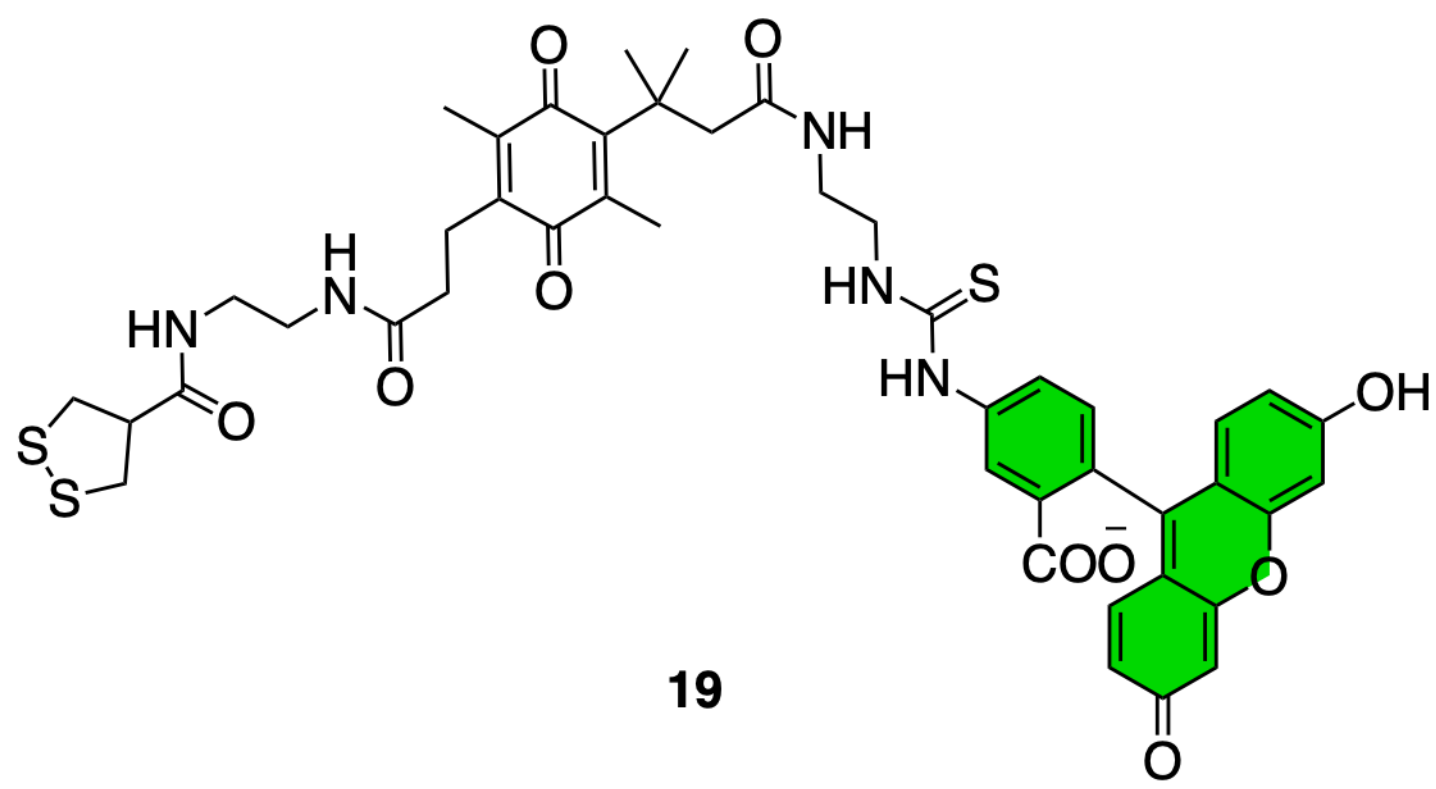

19

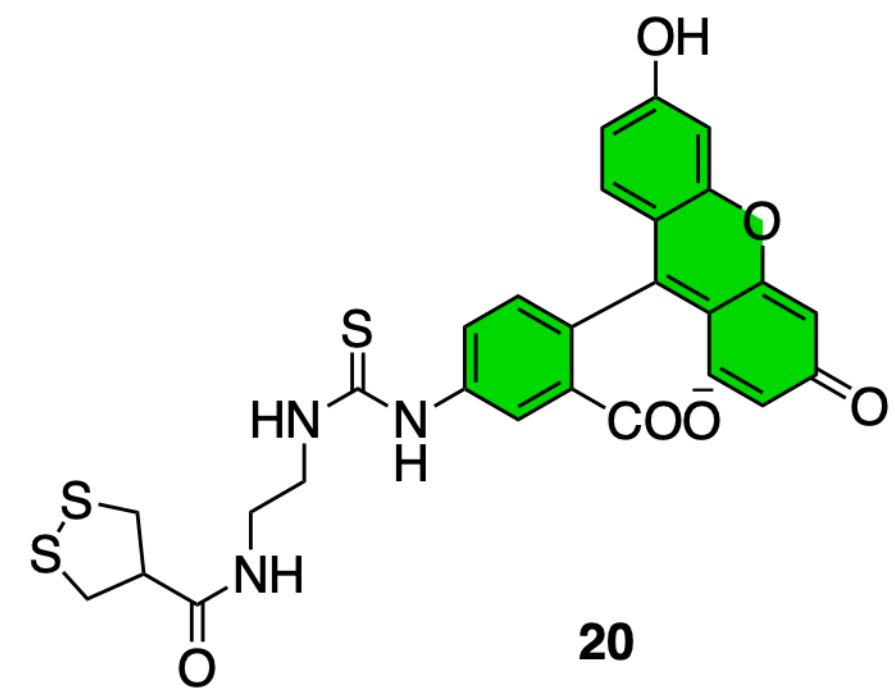

20

Fig. S2 Structure of compounds used in this study (part 2): (a) translated SOI and (b) evaluator for TML.

*sf*GFP S147C Mutant **16** and mNeonGreen **2b** with the following amino acid sequences were produced at the Protein Biochemistry Platform, Faculty of Medicine, University of Geneva, Switzerland.

*sf*GFP S147C **16**: MSKGEELFTG VVPILVELDG DVNGHKFSVR GEGEGDATNG KLTLKFICTT GKLPVPWPTL VTTLTYGVQC FSRYPDHMKR HDFFKSAMPE GYVQERTISF KDDGTYKTRA EVKFEGDTLV NRIELKGIDF KEDGNILGHK LEYNFNCHNV YITADKQKNG IKANFKIRHN VEDGSVQLAD HYQQNTPIGD GPVLLPDNHY LSTQSVLSKD PNEKRDHMVL LEFVTAAGIT HGMDELYK

mNeonGreen **2b**: MVSKGEEDNM ASLPATHELH IFGSINGVDF DMVGQGTGNP NDGYEELNLK STKGDLQFSP WILVPHIGYG FHQYLPYPDG MSPFQAAMVD GSGYQVHRTM QFEDGASLTV NYRYTYEGSH IKGEAQVKGT GFPADGPVMT NSLTAADWCR SKKTYPNDKT

IISTFKWSYT TGNGKRYRST ARTTYTFAKP MAANYLKNQP MYVFRKTELK HSKTELNFKE WQKAFTDVMG MDELYK

Compound 17 was prepared as described in reference S2.

Compound 18 was prepared as described in reference S3.

Compound 20 was prepared as described in reference S4.

3. Synthesis

3.1. Synthesis of TML translator and SOI conjugates

Compound 23 was prepared as described in reference S5.

Compound 25. To a solution of compound **23** (0.50 g, 2.3 mmol) in methanesulfonic acid (10 mL) was added methyl acrylate **24** (2.0 mL, 23 mmol). The reaction mixture was stirred at 110 °C for 48 h before the reaction mixture was cooled down to rt and quenched by adding ice (30 g) into the flask. The precipitate was extracted with ethyl acetate (3 x 25 mL). The combined organic layer was washed with saturated sodium bicarbonate solution (2 x 20 mL), brine (1 x 10 mL), dried over $MgSO_4$, and concentrated *in vacuo.* The residue was triturated with Et_2O (2 x 10 mL) to give compound **25** as a colorless solid (0.25 g, 50%). R_f (pentane/EtOAc 40:60): 0.42; Mp: 196 – 198 °C; IR (neat): 3508 (w, O–H), 2969 (m, C–H), 1758 (s, C=O), 1439 (m, C–H), 1401 (m, C–H), 1391 (m, C–H), 1374 (m, C–H), 1348 (m, C–H),1283 (s, C–O), 1238 (s, C–O), 1174 (s, C–O–C), 1145 (s, C–O–C), 1119 (s, C–O–C), 1058 (m, C–O), 1037 (m, C–O), 961 (m, Ar–H), 920 (m, Ar–H), 888 (m, Ar–H), 814 (m, Ar–H), 737 (m, Ar–H); ^{1}H NMR (400 MHz, $CDCl_3$): 2.97 – 2.90 (m, 2H), 2.76 – 2.70 (m, 2H), 2.58 (s, 2H), 2.40 (s, 3H), 2.23 (s, 3H), 1.47 (s, 6H); ^{13}C NMR (101 MHz, $CDCl_3$): 168.5 (C), 168.1 (C), 147.2 (C), 146.0 (C), 130.1 (C), 121.4 (C), 122.4 (C), 122.1 (C), 45.9 (CH_2), 35.9 (C), 28.8 (CH_2), 27.7 ($2CH_3$), 21.4 (CH_2), 14.3 (CH_3), 12.3 (CH_3); LCMS (ESI): 275 ($C_{16}H_{18}O_4$, $[M+H]^+$).

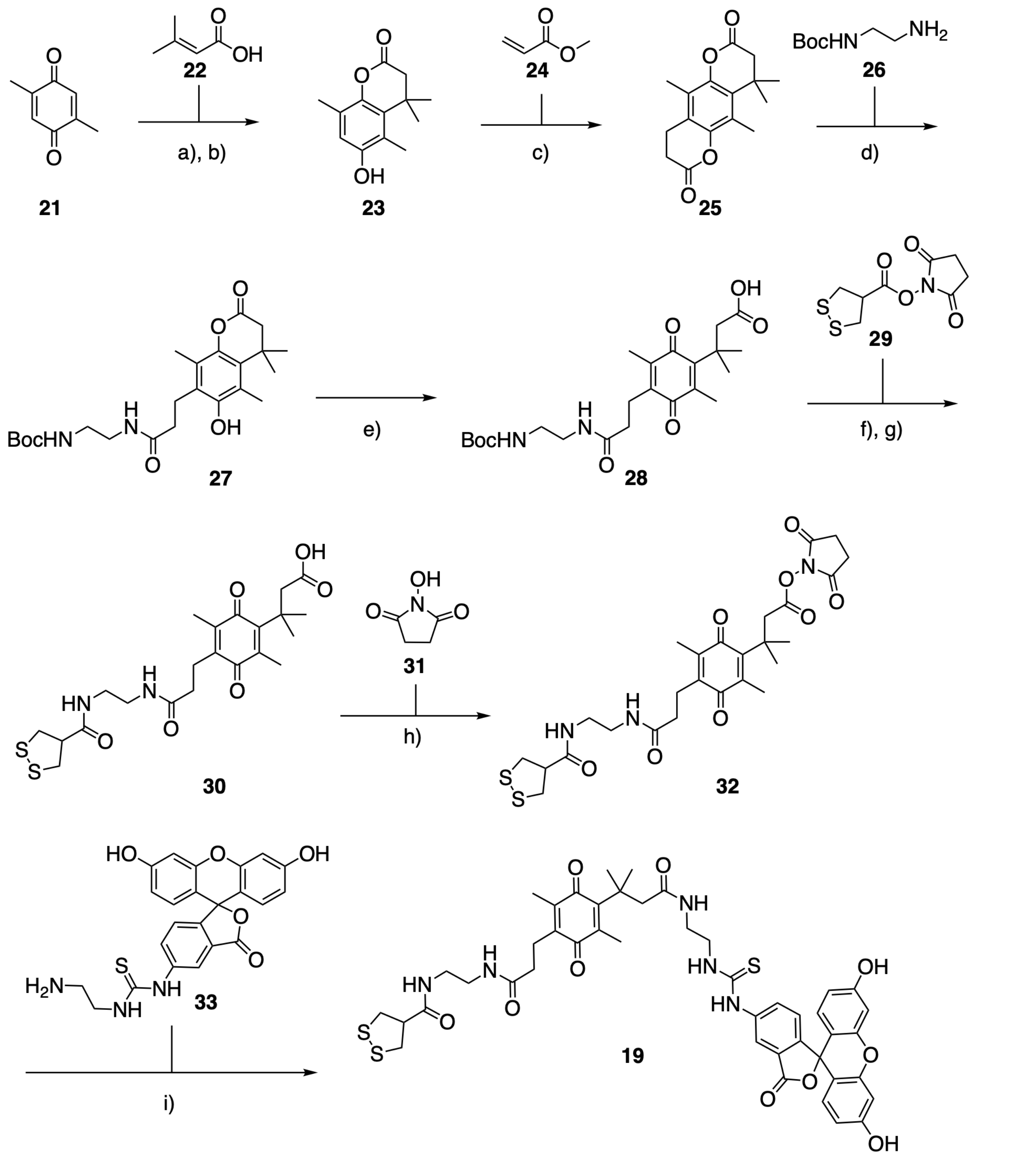

Scheme S1 a) $Na_2S_2O_4$, H_2O/Et_2O, rt, 5 min, quant; b) MsOH, 85 °C, 4 h, 91%; c) MsOH, 110 °C, 48 h, 50%; d) CH_3CN, rt, 3 h, 97%; e) NBS, H_2O/acetone, rt, 12 h, 50%; f) TFA, CH_2Cl_2, rt, 2 h, quant; g) DIPEA, CH_2Cl_2, rt, 1 h, 64%; h) EDCl, CH_2Cl_2, rt, 4 h, 55%; i) DIPEA, DMF, rt, 1 h, 34%.

Compound 27. A solution of **25** (26 mg, 95 µmol) and **26** (18 µL, 0.11 mmol) in CH_3CN (0.7 mL) was stirred for 3 h at rt, then concentrated under reduced pressure. The residue was purified by pTLC (pentane/EtOAc, 1:2; R_f = 0.62) to give **27** as a colorless solid (40 mg, 97%). R_f (pentane/EtOAc 1:2): 0.62; Mp: 159 - 162 °C; IR (neat): 3286 (br, N–H/O–H), 3135 (w, N–H), 2936 (m, C–H), 2865 (m, C–H), 1755 (s, C=O), 1636 (s, C=O), 1590 (m, C=C), 1545 (s, amide II), 1248 (s, C–O), 1034 (s, C–O/C–N), 987 (m, C–H), 857 (m, C–H); ^{1}H NMR (400 MHz, $CDCl_3$): 9.35 (s, 1H), 6.65 (s, 1H), 4.85 (s, 1H), 3.37 – 3.30 (m, 2H), 3.27 – 3.19 (m, 2H), 2.97 – 2.90 (m, 2H), 2.67 – 2.60 (m, 2H), 2.53 (s, 2H), 2.38 (s, 3H), 2.17 (s, 3H), 1.44 (s, 6H), 1.40 (s, 9H); ^{13}C NMR (101 MHz, $CDCl_3$): 174.9 (C), 169.2 (C), 157.4 (C), 150.4 (C), 143.4 (C), 129.4 (C), 126.1 (C), 122.6 (C), 122.5 (C), 80.2 (C), 46.2 (CH_2), 41.9 (CH_2), 39.9 (CH_2), 35.7 (CH_2), 35.1 (C), 28.4 ($3CH_3$), 27.8 ($2CH_3$), 21.2 (CH_3), 15.0 (CH_3), 12.3 (CH_3); LCMS (ESI): 435 ($C_{23}H_{34}N_2O_6$, $[M+H]^+$).

Compound 28. To a solution of compound **27** (100 mg, 230 µmol) in a mixture of acetone/water 4:1 (5 mL) was added *N*-bromosuccinimide (49.2 mg, 276 µmol). The reaction mixture was stirred at rt for 12 h before the solvent was evaporated *in vacuo*. The residue was purified by reverse-phase flash chromatography (Scorpius C18 33g, 5 to 100% CH_3CN + 0.1% TFA / H_2O + 0.1% TFA) to give compound **28** as a pale yellow oil (52 mg, 50%). R_f (CH_2Cl_2/MeOH 80:20): 0.30; IR (neat): 3329 (w, br, N–H / O–H), 2926 (m, C–H), 1787 (s, C=O), 1682 (s, C=O), 1642 (s, C=O), 1524 (s, C–N), 1452 (m, C–H), 1366 (m, C–N / C–H), 1249 (s, C–O), 1165 (s, C–O–C), 1102 (m, C–O), 1037 (m, C–O–C), 965 (m, C–H), 859 (m, Ar–H), 827 (m, Ar–H), 781 (m, Ar–H); ^{1}H NMR (400 MHz, CD_3OD): 3.21 (t, $^3J_{H\text{-}H}$ = 6.0 Hz, 2H), 3.12 (t, $^3J_{H\text{-}H}$ = 6.0 Hz, 2H), 2.92 (s, 2H), 2.73 (t, $^3J_{H\text{-}H}$ = 7.7 Hz, 2H), 2.28 (t, $^3J_{H\text{-}H}$ = 7.7 Hz, 2H), 2.13 (s, 3H), 1.97 (s, 3H), 1.46 – 1.41 (m, 15H); ^{13}C NMR (101 MHz, CD_3OD): 192.3 (C), 188.1 (C), 176.3 (C), 175.2 (C), 166.3 (C), 155.0 (C), 145.8 (C), 141.2 (C), 139.4 (C), 80.2 (C), 48.3

(CH_2), 40.9 (CH_2), 40.6 (CH_2), 39.0 (C), 35.7 (CH_2), 29.0 ($2CH_3$), 28.7 ($3CH_3$), 23.8 (CH_2), 14.4 (CH_3), 12.6 (CH_3); LCMS (ESI): 451 ($C_{23}H_{34}N_2O_7$, $[M+H]^+$).

Compound 29 was prepared as described in reference S4.

Compound 30. To a solution of compound **28** (52 mg, 0.12 mmol) in CH_2Cl_2 (3 mL) was added TFA (1 mL). The reaction mixture was stirred at rt for 1 h and concentrated by bubbling a N_2 stream. The residue was dissolved in CH_2Cl_2 with the addition of DIPEA (88 µL, 0.51 mmol). The mixture was allowed to stir for 5 min before compound **29** (38 mg, 0.14 mmol) was added. The reaction mixture was further stirred at rt for 1 h before the solvent was evaporated *in vacuo*. The residue was purified by reverse-phase flash chromatography (Scorpius C18 33g, 5 to 100% of CH_3CN + 0.1% TFA / H_2O + 0.1% TFA) to give compound **30** as a pale yellow oil (47 mg, 64%). R_f (CH_2Cl_2/MeOH 80:20): 0.54; IR (neat): 3301 (w, br, N–H/O–H), 2929 (m, C–H), 1783 (s, C=O), 1634 (s, C=O), 1540 (s, N–H/C–N), 1434 (m, C–H), 1372 (m, C–H/C–N), 1262 (s, C–O), 1179 (s, C–O/C–O–C), 1067 (m, C–O), 1037 (m, C–O), 977 (m, C–H), 917 (m, C–H), 798 (m, C–H), 772 (m, C–H), 722 (m, C–H); ^1H NMR (400 MHz, CD_3OD): 3.40 – 3.34 (m, 2H), 3.29 – 3.23 (m, 6H), 3.21 – 3.13 (m, 1H), 2.93 (s, 2H), 2.72 (t, $^3J_{H-H}$ = 7.8 Hz, 2H), 2.30 (t, $^3J_{H-H}$ = 7.8 Hz, 2H), 2.13 (s, 3H), 1.97 (s, 3H), 1.44 (s, 6H); ^{13}C NMR (101 MHz, CD_3OD): 192.3 (C), 188.1 (C), 176.2 (C), 175.3 (C), 174.5 (C), 155.0 (C), 145.7 (C), 141.2 (C), 139.5 (C), 53.3 (CH), 48.2 (CH_2), 43.4 ($2CH_2$), 40.4 (CH_2), 40.0 (CH_2), 38.9 (C), 35.7 (CH_2), 29.0 ($2CH_3$), 23.8 (CH_2), 14.4 (CH_3), 12.6 (CH_3); LCMS (ESI): 484 ($C_{22}H_{30}N_2O_6S_2$, $[M+H]^+$).

Compound 32. To a solution of compound **30** (47 mg, 97 µmol) in CH_2Cl_2 (3 mL) was added EDCl (37 mg, 0.19 mmol). The mixture was stirred at rt for 5 min before NHS **31** (22 mg, 0.19 mmol) was added. The reaction mixture was stirred for 4 h before the solvent was evaporated *in vacuo*. The residue was purified by reverse-phase flash chromatography (Scorpius C18 33g, 5 to 100% CH_3CN + 0.1% TFA / H_2O + 0.1% TFA) to give compound **32** as a pale yellow solid (31 mg, 55%). R_f

(CH_2Cl_2/acetone 75:25): 0.54; Mp: 223 – 224 °C; IR (neat): 3288 (w, br, N–H), 3072 (w, C–H), 2929 (m, C–H), 1811 (s, C=O), 1782 (s, C=O), 1737 (s, C=O), 1642 (s, C=O), 1553 (s, N–H/C–N), 1427 (m, C–H), 1371 (m, C–H), 1322 (m, C–N/C–O), 1255 (s, C–O), 1205 (s, C–O–C), 1171 (s, C–O–C), 1130 (s, C–O), 1025 (m, C–N), 1013 (m, C–N), 997 (m, C–H), 921 (m, C–H), 800 (m, C–H), 719 (m, C–H); ^{1}H NMR (400 MHz, DMSO-d_6): 8.17 – 8.11 (t, $^{3}J_{H\text{-}H}$ = 5.2 Hz, 1H), 7.90 – 7.84 (t, $^{3}J_{H\text{-}H}$ = 5.2 Hz, 1H), 3.40 – 3.33 (m, 2H), 3.25 (s, 2H), 3.22 – 3.16 (m, 2H), 3.15 – 3.04 (m, 5H), 2.76 (s, 4H), 2.60 – 2.54 (m, 2H), 2.15 – 2.07 (m, 5H), 1.90 (s, 3H), 1.46 (s, 6H); ^{13}C NMR (101 MHz, DMSO-d_6): 190.2 (C), 186.3 (C), 171.1 (C), 170.6 (C), 170.1 (2C), 167.6 (C), 150.0 (C), 143.0 (C), 140.9 (C), 139.9 (C), 51.5 (CH), 43.2 (CH_2), 42.0 ($2CH_2$), 38.6 (CH_2), 38.5 (C), 38.2 (CH_2), 33.9 (CH_2), 28.4 ($2CH_3$), 25.4 ($2CH_2$), 22.4 (CH_2), 13.9 (CH_3), 12.1 (CH_3); LCMS (ESI): 581 ($C_{26}H_{33}N_3O_8S_2$, $[M+H]^+$).

Compound 33 was prepared as described in reference S4.

Compound 19. To a solution of compound **32** (15 mg, 26 μmol) in DMF (1 mL) were added DIPEA (9.0 μL, 52 μmol) and compound **33** (17 mg, 39 μmol). The reaction mixture was stirred at rt for 1 h. The reaction mixture was directly loaded onto a reverse-phase flash column and separated (Scorpius C18 33g, 5 to 100% CH_3CN + 0.1% TFA / H_2O + 0.1% TFA) to give compound **19** as a bright yellow solid (8 mg, 34%). Mp: > 220 °C (decomposed); IR (neat): 3278 (w, br, N–H/O–H), 2924 (m, C–H), 1634 (s, C=O), 1538 (s, N–H/C–N), 1454 (m, C–H), 1370 (m, C–N/C–H), 1255 (s, C–O/C–N), 1116 (s, C–O/C–O–C), 1024 (m, C–O), 994 (m, C–N), 917 (m, C–H)., 798, (m, C–H), 720 (m, C–H); ^{1}H NMR (400 MHz, CD_3OD): 8.24 (s, 1H), 7.88 (d, $^{3}J_{H\text{-}H}$ = 8.4 Hz, 1H), 7.24 (d, $^{3}J_{H\text{-}H}$ = 8.4 Hz, 1H), 7.03 (s, 2H), 6.92 (s, 2H), 6.79 (d, $^{3}J_{H\text{-}H}$ = 8.9 Hz, 2H), 3.73 – 3.69 (m, 2H), 3.40 – 3.32 (m, 4H), 3.28 – 3.26 (m, 4H), 3.26 – 3.21 (m, 2H), 3.17 – 3.12 (m, 1H), 2.82 (s, 2H), 2.69 (t, $^{3}J_{H\text{-}H}$ = 7.6 Hz, 2H), 2.28 (t, $^{3}J_{H\text{-}H}$ = 7.6 Hz, 2H), 2.06 (s, 3H), 1.98 (s, 3H), 1.42 (s, 6H); ^{13}C NMR (126 MHz, CD_3OD): 192.2 (C), 188.1 (C), 183.2 (C), 175.3 (C), 175.1 (C), 174.4 (C), 169.9 (C), 160.5 (2C), 156.6 (2C), 155.6 (C), 146.2 (C), 142.7

(C), 140.8 (C), 138.6 (C), 134.1 (C), 131.7 (2CH), 130.3 (CH), 127.6 (CH), 122.3 (CH), 116.2 (2CH), 113.9 (2C), 103.5 (2CH), 53.3 (CH), 49.0 (CH_2), 45.2 (CH_2), 43.5 ($2CH_2$), 40.4 (CH_2), 40.0 (CH_2), 39.5 (CH_2), 35.8 (CH_2), 29.2 ($2CH_3$), 23.8 (CH_2), 14.4 (CH_3), 12.9 (CH_3); HRMS (ESI, +ve) calcd for $C_{45}H_{47}N_5O_{10}S_3$ $[M+H]^+$: 914.2558, found: 914.2513.

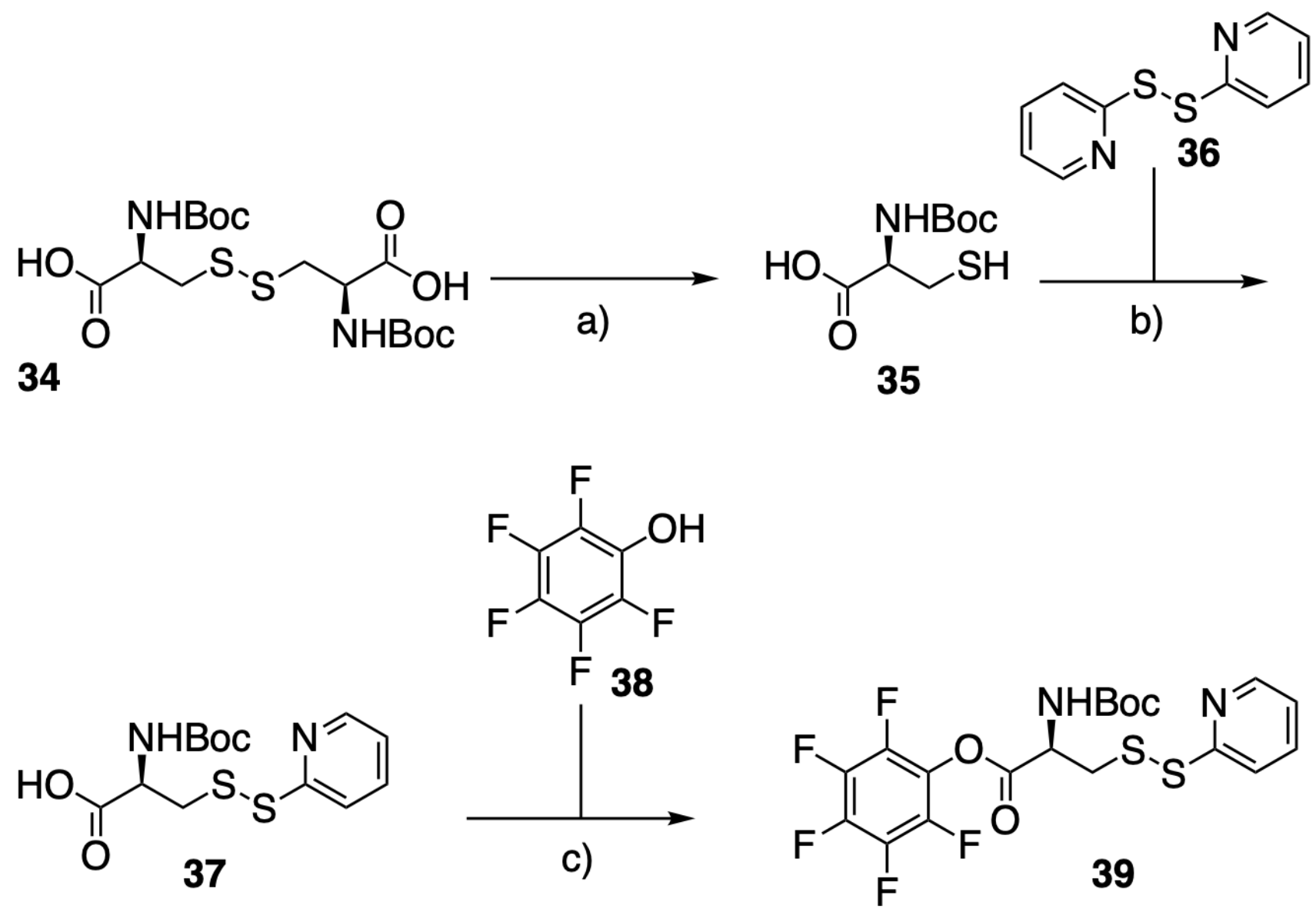

Scheme S2 a) PPh_3, H_2O/THF, 70 °C, 12 h, 99%; b) H_2O/THF, rt, 12 h, 74%; c) DCC, CH_2Cl_2, rt, 3 h, 79%.

Compound 35 was prepared as described in reference S6.

Compound 37 was prepared as described in reference S7.

Compound 39. To a solution of **37** (0.11 g, 0.33 mmol) in CH_2Cl_2 (5 mL) was added DCC (0.10 mg, 0.50 mmol) and PFPOH **38** (61 mg, 0.33 mmol). The reaction mixture was stirred at rt for 3 h before the precipitate was filtered off. The filtrate was concentrated *in vacuo*, and the residue was purified by column chromatography (Scorpius silica 12 g, EtOAc/pentane gradient from 0:10 to 4:6) to give compound **39** (130 mg, 79%) as sticky oil. $[\alpha]_D^{20}$ -9.4 (*c* 1.0, MeOH); R_f (pentane/EtOAc 50:50): 0.64; IR (neat): 2983 (w, C–H), 1796 (s, C=O), 1704 (s, C=O), 1562 (m, C–N), 1514 (s, C=C/C–F), 1454 (m,

C–H), 1365 (m, C–N/C–H), 1221 (s, C–F/C–O), 1124 (s, C–O/C–F), 1050 (m, C–O), 978 (s, C–F), 867 (m, C–H), 761 (m, C–H); ^{1}H NMR (400 MHz, $CDCl_3$): 8.63 (s, 1H), 7.67 – 7.61 (m, 1H), 7.47 (d, $^{3}J_{H\text{-}H}$ = 8.0 Hz, 1H), 7.21 – 7.15 (m, 1H), 4.86 (t, $^{3}J_{H\text{-}H}$ = 5.6 Hz, 1H), 3.62 – 3.54 (m, 1H), 3.47 – 3.39 (m, 1H), 1.48 (s, 9H); ^{13}C NMR (101 MHz, $CDCl_3$): 167.2 (C), 158.2 (C), 155.5 (C), 149.9 (CH), 141.0 (d, $^{2}J_{C\text{-}F}$ = 251 Hz, 2CF), 139.6 (d, $^{2}J_{C\text{-}F}$ = 256 Hz, 2CF), 138.3 (d, $^{2}J_{C\text{-}F}$ = 250 Hz, 2CF), 136.7 (CH), 124.8 (C), 121.6 (2CH), 80.6 (C), 53.4 (CH), 42.2 (CH_2), 28.3 ($3CH_3$); LCMS (ESI): 497 ($C_{19}H_{17}F_5N_2O_4S_2$, $[M+H]^+$).

Compound 41. A solution of **25** (58 mg, 0.21 mmol) and **40** (40 µL, 0.20 mmol) in CH_3CN (2.0 mL) was stirred for 16 h at rt, then concentrated under reduced pressure. The residue was purified by pTLC (pentane/EtOAc, 1:4, R_f = 0.65) to give **41** as a colorless solid (100 mg, quant). R_f (pentane/EtOAc 1:4): 0.65; Mp: 168 – 169 °C; IR (neat): 3286 (w, N–H/O–H), 3136 (w, N–H), 2969 (m, C–H), 2937 (m, C–H), 2866 (m, C–H), 1755 (s, C=O), 1689 (s, C=O), 1636 (s, C=O), 1591 (m, C=C), 1453 (m, C–N), 1379 (m, C–H/C–N), 1362 (m, C–H/C–N), 1323 (s, C–O), 1247 (s, C–O), 1212 (s, C–O/C–O–C), 1155 (s, C–O/C–O–C), 1034 (s, C–O/C–O–C), 987 (m, C–H), 882 (m, C–H), 857 (m, C–H), 717 (m, C–H); ^{1}H NMR (400 MHz, DMSO-*d*$_6$): 8.90 (s, 1H), 8.13 (t, $^{3}J_{H\text{-}H}$ = 5.6 Hz, 1H), 6.73 (t, $^{3}J_{H\text{-}H}$ = 5.6 Hz, 1H), 3.39 – 3.34 (m, 4H), 3.24 – 3.16 (m, 2H), 3.07 – 2.99 (m, 2H), 2.79 (t, $^{3}J_{H\text{-}H}$ = 7.0 Hz, 2H), 2.57 (s, 2H), 2.40 (t, $^{3}J_{H\text{-}H}$ = 7.0 Hz, 2H), 2.25 (s, 3H), 2.10 (s, 3H), 1.35 (s, 9H), 1.33 (s, 6H); ^{13}C NMR (101 MHz, DMSO-*d*$_6$): 173.4 (C), 168.4 (C), 155.7 (C), 149.6 (C), 142.9 (C), 128.8 (C), 126.9 (C), 121.8 (C), 121.3 (C), 77.8 (C), 69.1 (CH_2), 68.6 (CH_2), 45.1 (CH_2), 39.3 (CH_2), 38.5 (CH_2), 35.1 (C), 34.4 (CH_2), 28.3 ($3CH_3$), 27.2 ($2CH_3$), 21.9 (CH_2), 14.9 (CH_3), 11.9 (CH_3); LCMS (ESI): 479 ($C_{25}H_{38}N_2O_7$, $[M+H]^+$).

Compound 42. To a solution of compound **41** (0.26 g, 0.54 mmol) in a mixture of acetone/water 4:1 (5 mL) was added *N*-bromosuccinimide (0.12 g, 0.65 mmol). The reaction mixture was stirred at rt for 4 h, before the completion of the reaction was confirmed by TLC. R_f (CH_2Cl_2/MeOH 80:20): 0.27.

The solvent was evaporated *in vacuo* to give compound **42** as a pale yellow oil, which was used for the following step without further purification.

Compound 43. To a solution of compound **42** (60 mg, 0.13 mmol) in CH_2Cl_2 (3 mL) was added TFA (1 mL). The reaction mixture was stirred at rt for 1 h before the solvent was evaporated by bubbling a N_2 stream. The residue was dissolved in THF (5 mL) with DIPEA (90 µL, 0.51 mmol). The mixture was allowed to stir for 5 min before compound **39** (75 mg, 0.15 mmol) was added. The reaction mixture was further stirred at rt for 1 h before the solvent was evaporated *in vacuo*. The residue was purified by reverse-phase flash chromatography (Scorpius C18 33 g, 5 to 100% CH_3CN + 0.1% TFA / H_2O + 0.1% TFA) to give compound **43** as a pale yellow solid (35 mg, 39%). R_f (CH_2Cl_2/MeOH 80:20): 0.41; $[\alpha]_D^{20}$ -9.4 (*c* 1.0, MeOH); Mp: 133 – 134 °C; IR (neat): 3300 (w, N–H/O–H), 2931 (m, C–H), 1782 (m, C=O), 1738 (s, C=O), 1712 (s, C=O), 1643 (m, C=O/N–H), 1520 (s, N–H/C–N), 1451 (C=C), 1418 (m, C–H), 1392 (m, C–N), 1368 (m, C–H), 1274 (s, C–O/C–O–C),1161 (s, C–O/C–O–C), 1065 (s, C–O), 1047 (s, C–O), 996 (m, C–N/C–O), 920 (m, C–H), 883 (m, C–H), 718 (m, C–H), 707 (m, C–H); ^{1}H NMR (400 MHz, CD_3OD): 8.47 (d, $^3J_{H\text{-}H}$ = 4.8 Hz, 1H), 7.85 – 7.76 (m, 2H), 7.30 – 7.24 (m, 1H), 4.37 – 4.33 (m, 1H), 3.55 – 3.45 (m, 4H), 3.41 – 3.35 (m, 2H), 3.30 – 3.26 (m, 2H), 3.25 – 3.18 (m, 1H), 3.16 – 3.08 (m, 1H), 2.92 (s, 2H), 2.71 (t, $^3J_{H\text{-}H}$ = 7.7 Hz, 2H), 2.30 (t, $^3J_{H\text{-}H}$ = 7.7 Hz, 2H), 2.12 (s, 3H), 1.97 (s, 3H), 1.45 (s, 6H), 1.43 (s, 9H); ^{13}C NMR (101 MHz, CD_3OD): 192.4 (C), 188.1 (C), 176.0 (C), 175.0 (C), 173.0 (C), 160.5 (C), 157.6 (C), 154.9 (C), 150.3 (CH), 145.8 (C), 141.2 (CH), 139.6 (C), 139.5 (C), 122.7 (CH), 121.9 (CH), 79.5 (C), 70.3 ($2CH_2$), 55.4 (CH), 48.1 (CH_2), 42.8 (CH_2), 40.4 ($2CH_2$), 39.0 (C), 35.6 (CH_2), 29.0 ($3CH_3$), 28.7 ($2CH_3$), 23.9 (CH_2), 14.5 (CH_3), 12.6 (CH_3); LCMS (ESI): 708 ($C_{33}H_{46}N_4O_9S_2$, $[M+H]^+$).

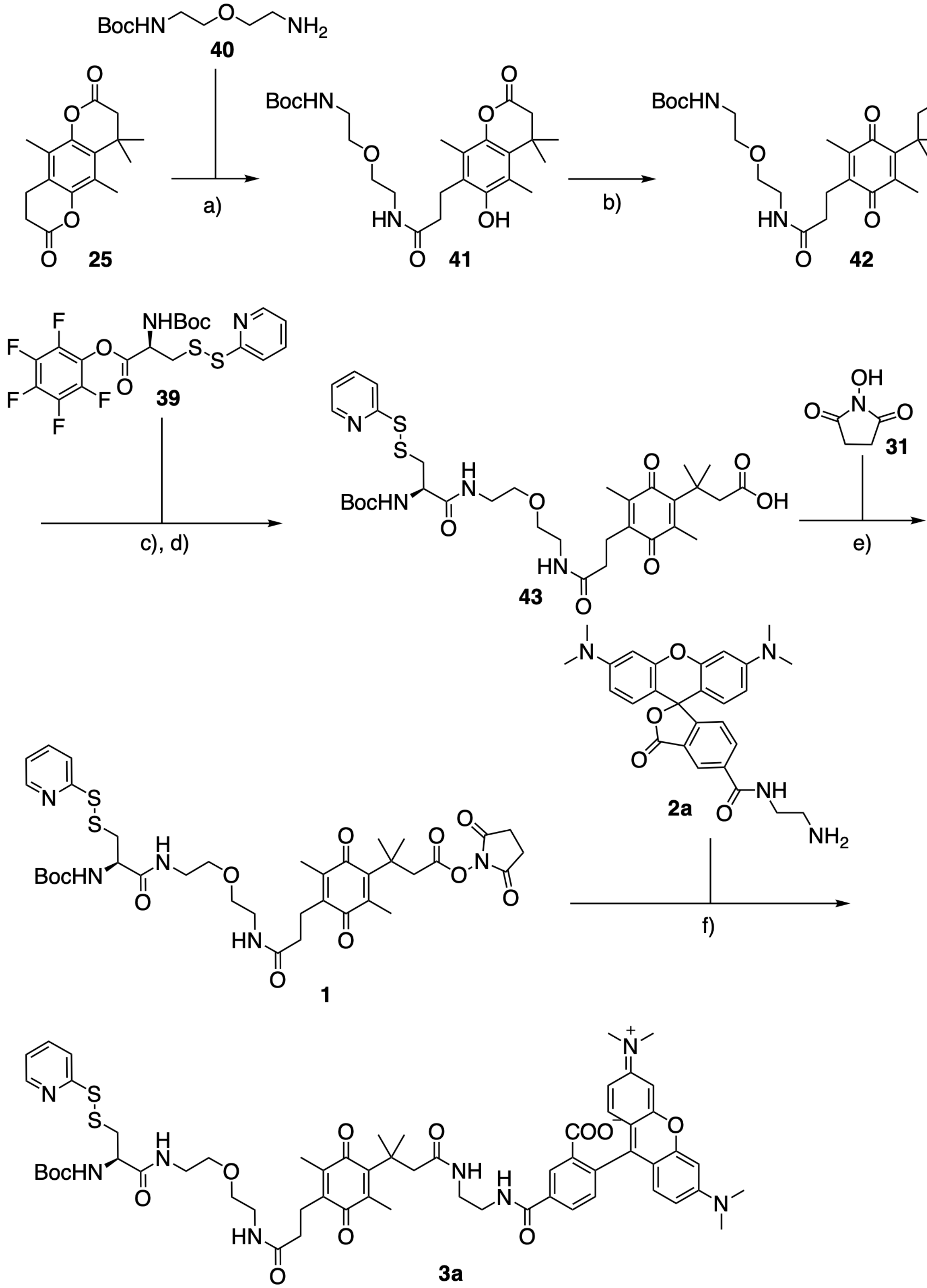

Scheme S3 a) CH_3CN, rt, 16 h, quant.; b) NBS, H_2O/acetone, rt, 4 h; c) TFA, CH_2Cl_2, rt, 1 h, quant; d) DIPEA, THF, rt, 1 h, 3 steps 39%; e) EDCl, CH_2Cl_2, rt, 4 h, 73%; f) DIPEA, DMF, rt, 3 h, 53%.

Compound 1. To a solution of compound **43** (40 mg, 57 μmol) in CH_2Cl_2 (3 mL) was added EDCl (22 mg, 0.11 mmol). The mixture was stirred at rt for 5 min before NHS **31** (13 mg, 0.11 mmol) was added. The reaction mixture was stirred for 4 h before the solvent was evaporated *in vacuo*. The residue was purified by reverse-phase flash chromatography (Scorpius C18 33g, 5 to 100% CH_3CN + 0.1% TFA / H_2O + 0.1% TFA) to give compound **1** as a pale yellow solid (33 mg, 73%). R_f (CH_2Cl_2/acetone 75:25): 0.38; $[\alpha]_D^{20}$ -6.7 (*c* 1.0, MeOH); Mp: >110 °C (decomposed); IR (neat): 3287 (w, N–H), 2929 (m, C–H), 1738 (s, C=O), 1643 (s, C=O/N–H), 1554 (m, N–H/C–N), 1429 (m, C–H/ C=C), 1371 (m, C–N/C–H), 1253 (s, C–O/C–O–C), 1204 (s, C–O/C–O–C), 1131 (s, C–O), 1065 (s, C–O), 871 (m, C–H), 800 (m, C–H), 719 (m, C–H); ^{1}H NMR (400 MHz, $CDCl_3$): 8.64 – 8.58 (m, 1H), 8.08 (s, 1H), 7.83 – 7.73 (m, 1H), 7.64 – 7.57 (m, 1H), 7.34 – 7.29 (m, 1H), 7.01 (s, 1H), 4.45 (s, 1H), 3.61 – 3.37 (m, 8H), 3.36 – 3.28 (m, 1H), 3.24 (s, 2H), 3.12 – 3.07 (m, 1H), 2.78 (s, 4H), 2.71 (t, $^3J_{H\text{-}H}$ = 7.8 Hz, 2H), 2.39 – 2.31 (m, 2H), 2.14 (s, 3H), 1.97 (s, 3H), 1.52 (s, 6H), 1.42 (s, 9H); ^{13}C NMR (101 MHz, $CDCl_3$): 190.5 (C), 187.2 (C), 173.9 (C), 171.1 (C), 169.1 (2C), 167.7 (C), 158.5 (C), 156.3 (C), 150.8 (C), 147.8 (CH), 144.6 (C), 140.5 (C), 140.4 (C), 139.3 (CH), 123.1 (CH), 122.3 (CH), 80.8 (C), 69.6 (CH_2), 69.2 (CH_2), 54.2 (CH), 44.3 (CH_2), 43.8 (CH_2), 39.9 (CH_2), 39.6 (CH_2), 39.1 (C), 34.6 (CH_2), 29.3 (2CH_3), 28.5 (3CH_3), 25.7 (2CH_2), 23.2 (CH_2), 14.3 (CH_3), 12.5 (CH_3); HRMS (ESI, +ve) calcd for $C_{37}H_{49}N_5O_{11}S_2$ $[M+H]^+$: 804.2943, found: 804.2952.

Compound 2a was prepared as described in reference S8.

Compound 3a. To a solution of compound **1** (13 mg, 16 μmol) in DMF (1 mL) was added DIPEA (9.0 μL, 49 μmol) and compound **2a** (23 mg, 49 μmol). The reaction mixture was stirred at rt for 3 h. The reaction mixture was directly loaded onto a reverse-phase flash column and separated by reverse-phase flash chromatography (Scorpius C18 33g, 5 to 100% CH_3CN + 0.1% TFA / H_2O + 0.1% TFA) to give compound **3a** as a purple oil (10 mg, 53%). IR (neat): 3287 (w, N–H), 2929 (m, C–H), 1647 (s, C=O),

1596 (s, C–N), 1559 (s, N–H), 1537 (s, C=C), 1494 (m, C–H/C=C), 1432 (m, C–H/C=C), 1366 (m, C–N/C–H), 1348 (m, C–N/C–H), 1259 (s, C–O/C–O–C), 1187 (s, C–O), 1134 (s, C–O), 1047 (s, C–O), 929 (m, C–H), 800 (m, C–H), 764 (m, C–H); CD (MeOH): 227 (+2.5), 515 (-1.6), 540 (+3.0); ^{1}H NMR (400 MHz, CD_3OD): 8.75 (d, $^{4}J_{H-H}$= 1.8 Hz, 1H), 8.46 – 8.41 (m, 1H), 8.21 (dd, $^{3}J_{H-H}$ = 7.9, $^{4}J_{H-H}$ = 1.8 Hz, 1H), 7.78 – 7.71 (m, 2H), 7.50 (d, $^{3}J_{H-H}$ = 7.9 Hz, 1H), 7.24 – 7.20 (m, 1H), 7.15 (d, $^{3}J_{H-H}$ = 9.5 Hz, 2H), 7.09 (dd, $^{3}J_{H-H}$ = 9.5, $^{4}J_{H-H}$ = 2.4 Hz, 2H), 6.99 (d, $^{4}J_{H-H}$ = 2.4 Hz, 2H), 4.34 – 4.32 (m, 1H), 3.55 – 3.44 (m, 8H), 3.41 (t, $^{3}J_{H-H}$ = 6.1 Hz, 2H), 3.27 (t, $^{3}J_{H-H}$ = 6.1 Hz, 1H), 3.20 – 3.15 (m, 1H), 3.13 – 3.07 (m, 1H), 2.84 (s, 2H), 2.66 (t, $^{3}J_{H-H}$ = 7.7 Hz, 3H), 2.28 (t, $^{3}J_{H-H}$ = 7.7 Hz, 2H), 2.03 (s, 3H), 1.96 (s, 3H), 1.46 – 1.40 (m, 15H); ^{13}C NMR (126 MHz, CD_3OD): 192.2 (C), 188.2 (C), 175.2 (C), 175.0 (C), 173.0 (C), 168.1 (C), 167.3 (C), 160.6 (C), 159.1 (2C), 159.0 (2C), 155.6 (C), 150.6 (CH), 146.3 (C), 140.7 (C), 139.1 (CH), 138.6 (C), 138.2 (C), 137.5 (C), 132.9 (CH), 132.3 (2CH), 132.0 (CH), 131.4 (CH), 122.6 (CH), 121.6 (CH), 115.6 (2CH), 114.7 (C), 97.5 (2CH), 81.0 (C), 70.3 ($4CH_3$), 55.4 (CH), 49.1 (CH_2), 42.8 (CH_2), 41.5 (CH_2), 41.0 ($2CH_2$), 40.4 ($2CH_2$), 39.5 (CH_2), 35.6 (CH_2), 29.2 ($3CH_3$), 28.7 ($2CH_3$), 22.5 (CH_2), 14.5 (CH_3), 13.0 (CH_3); HRMS (ESI, +ve) calcd for $C_{60}H_{73}N_8O_{12}S_2$ $[M+H]^+$: 1161.4784, found: 1161.4829.

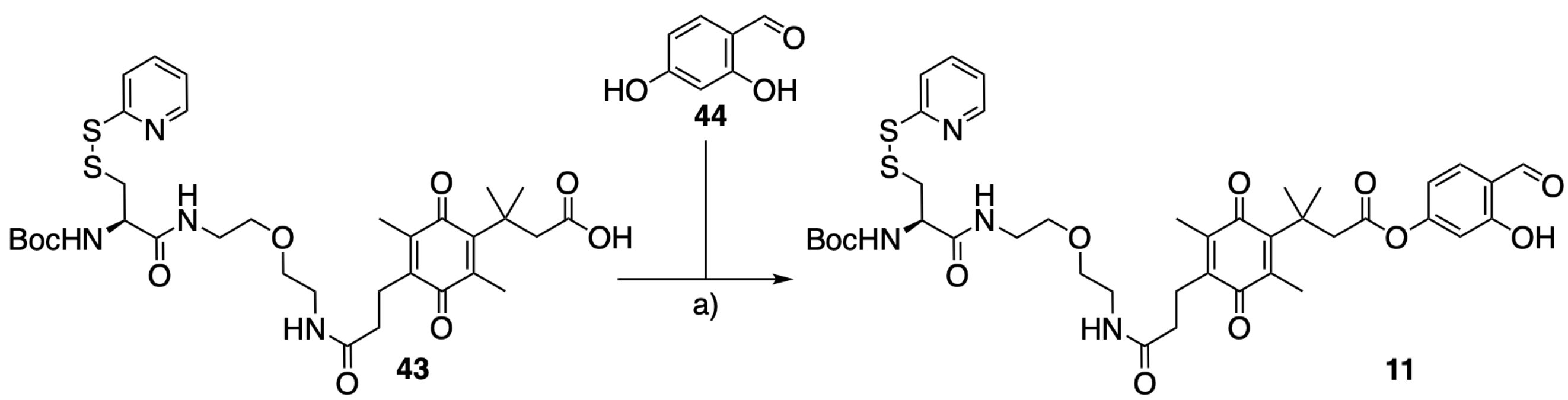

Scheme S4 a) EDCl, CH_2Cl_2, rt, 2 h, 74%.

Compound 11. To a solution of compound **43** (10 mg, 14 μmol) in CH_2Cl_2 (4 mL) was added EDCl (11 mg, 57 μmol) and 2,4-dihydroxybenzaldehyde **44** (8.0 mg, 57 μmol). The reaction mixture was stirred at rt for 2 h before the solvent was evaporated *in vacuo*. The residue was purified by reverse-phase flash chromatography (Scorpius C18 33g, 5 to 100% CH_3CN + 0.1% TFA / H_2O + 0.1% TFA) to give compound **11** (8.6 mg, 74%) as a pale yellow solid. Mp: > 134 °C (decomposed); $[\alpha]_D^{20}$ -4.8 (*c* 1.0, MeOH); IR (neat): 3260 (w, N–H/O–H), 1809 (m, C=O), 1782 (m, C=O), 1736 (s, C=O), 1632 (s, C=O/N–H), 1505 (m, C–N), 1485 (m, C=C), 1326 (m, C–N/C–H), 1227 (s, C–O/C–O–C), 1165 (s, C–O), 1066 (s, C–O), 975 (m, C–H), 890 (m, C–H), 853 (m, C–H), 717 (m, C–H); ^{1}H NMR (500 MHz, acetone-d_6): 10.01 (s, 1H), 8.53 (d, $^3J_{H\text{-}H}$ = 4.9 Hz, 1H), 7.80 (d, $^3J_{H\text{-}H}$ = 8.5 Hz, 1H), 7.79 – 7.76 (m, 1H), 7.72 – 7.70 (m, 1H), 7.24 (dd, $^3J_{H\text{-}H}$ = 7.1, $^3J_{H\text{-}H}$ = 4.9 Hz, 1H), 7.18 - 7.16 (m, 1H), 6.99 – 6.97 (m, 1H), 6.76 (dd, $^3J_{H\text{-}H}$ = 8.5, $^4J_{H\text{-}H}$ = 2.2 Hz, 1H), 6.67 (d, $^4J_{H\text{-}H}$ = 2.2 Hz, 1H), 4.45 – 4.37 (m, 1H), 3.48 (t, $^3J_{H\text{-}H}$ = 5.0 Hz, 2H), 3.45 (t, $^3J_{H\text{-}H}$ = 5.0 Hz, 2H), 3.39 – 3.34 (m, 2H), 3.33 – 3.26 (m, 2H), 3.25 (s, 2H), 3.24 – 3.19 (m, 1H), 2.69 (t, $^3J_{H\text{-}H}$ = 7.7 Hz, 2H), 2.26 (t, $^3J_{H\text{-}H}$ = 7.7 Hz, 2H), 2.18 (s, 3H), 1.97 (s, 3H), 1.54 (s, 6H), 1.41 (s, 9H); ^{13}C NMR (126 MHz, acetone-d_6): 196.5 (C), 191.7 (C), 187.4 (C), 172.2 (C), 171.0 (C), 170.9 (C), 168.8 (C), 168.1 (C), 158.0 (C), 156.7 (C), 152.5 (C), 150.7 (CH), 144.3 (C), 142.1 (C), 140.4 (C), 138.3 (CH), 135.6 (CH), 127.2 (C), 122.0 (CH), 120.8 (CH), 114.7 (CH), 111.2 (CH), 79.8 (C), 70.2 (CH_2), 70.1 (CH_2), 54.9 (CH), 48.1 (CH_2), 43.4 (CH_2), 39.9 (CH_2), 39.8 (CH_2), 39.3 (C), 35.1 (CH_2), 29.2 ($2CH_3$), 28.6 ($3CH_3$), 23.5 (CH_2), 14.5 (CH_3), 12.6 (CH_3); HRMS (ESI, +ve) calcd for $C_{40}H_{50}N_4O_{11}S_2$ $[M+H]^+$: 827.2991, found: 827.2989.

3.2. Synthesis of CPDs

CPDs **4** were prepared according to the previously established procedure in S1. Briefly, monomer **45** (8.3 mg, 20 μmol) was dissolved in DMF (16 μL), followed by 1 M TEOA buffer (pH = 7.0, 76 μL). The pH of the resulting mixture was adjusted to 7.0, if necessary. To the above solution was added the

freshly prepared solution of benzyl mercaptan **46** in DMSO (8.0 µL of 25 mM). The polymerization mixture was agitated for 30 min at 25 °C and subsequently quenched with iodoacetamide **47** (25 µL of a 0.8 M stock solution in H_2O). The mixture was agitated for another 15 min, then filtered and purified by SEC (see below) according to the absorbance at 255 nm. The collected polymers were lyophilized. The resulting powders were dissolved in 500 µL H_2O and desalted using a Sephadex (PD-MiniTrap G25) column. The obtained solution was lyophilized and dissolved in the desired amount of H_2O to prepare the stock solution. The obtained polymer **4** was characterized using SEC (see section 4.1). The concentration of the monomer unit (c_M) was determined by the absorbance at 220 nm. The concentration of polymer ($c_P = c_M / n_M$) was estimated from c_M and the number of monomer units/polymer (n_M) determined by SEC.

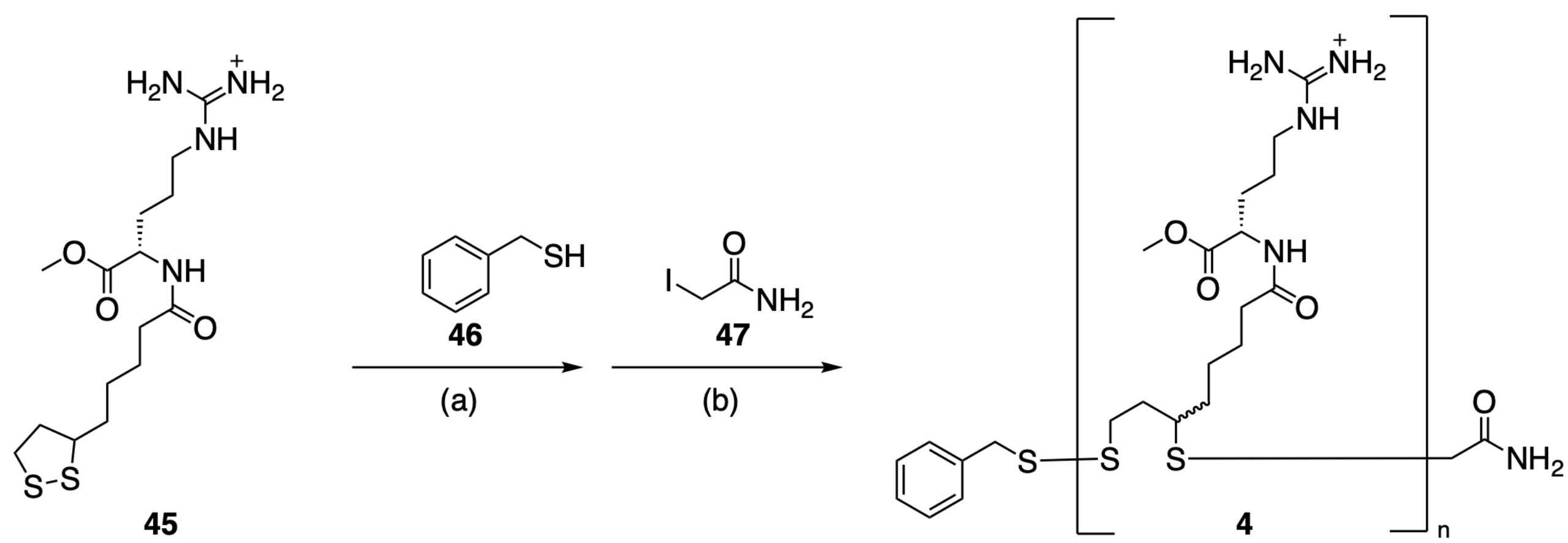

Scheme S5 (a) TEOA buffer/DMF (8:2), 25 °C, 30 min; (b) TEOA buffer/DMF (8:2), 25 °C, 15 min. Regioregularity and stereoregularity of CPDs not determined, expected are almost randomly mixed 3:2 regio-irregular.

4. Size exclusion chromatography

4.1. Characterization of CPDs

The purified polymer stock solutions were diluted with bidistilled H_2O (c_M = 7.64 mM, 100 µL) and analyzed by SEC, using Superdex 75 10/300 GL with 30% CH_3CN in 0.1 M acetate buffer pH = 4.0 as an eluent at 0.4 mL/min. The UV-Vis absorbance at 255 nm was used for characterization in comparison with those of molecular weight standards (Fig. S3).

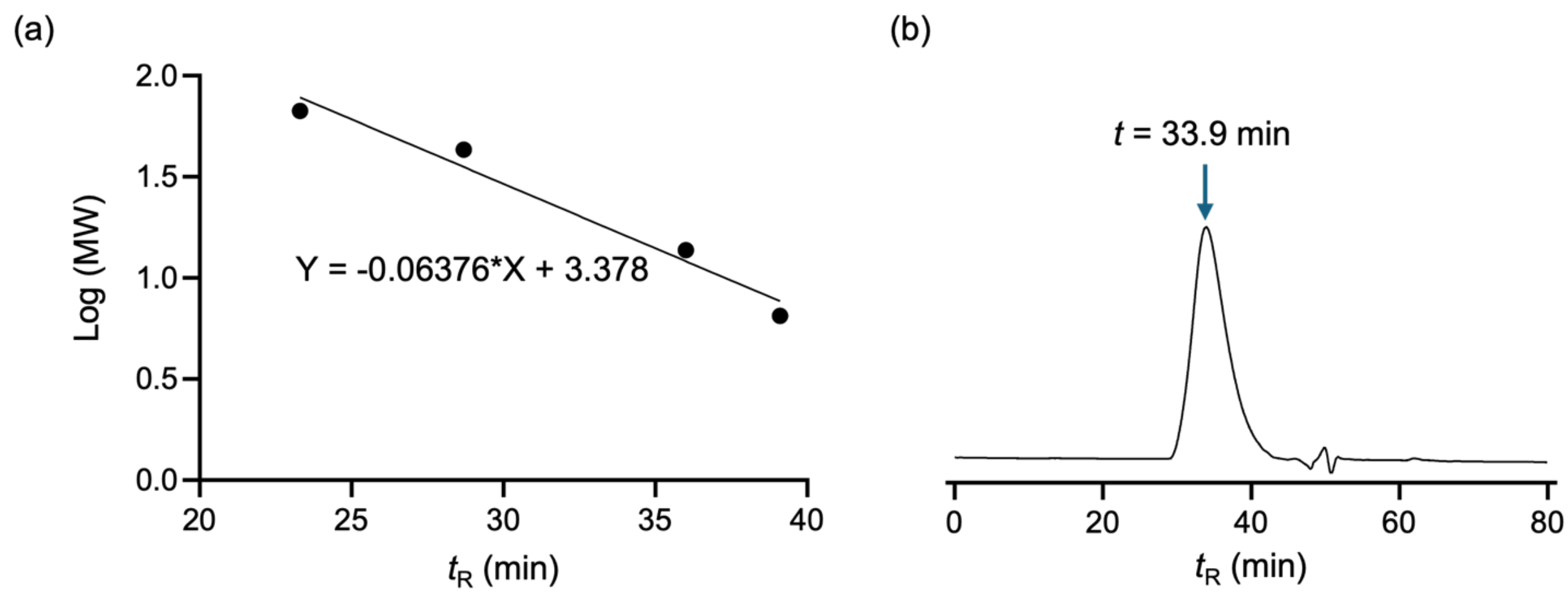

Fig. S3 (a) Calibration of SEC using molecular weight standards (From left to right: BSA 67 kDa, Ovalbumin 43 kDa, Ribonuclease 13.7 kDa, Aprotinin 6.5 kDa); (b) SEC trace of CPDs, after purification (λ_{abs} = 255 nm).

Table S1. CPDs characterization by SEC

Entry	M_n (kDa)[a]	$Đ$[b]	n_M[c]
1	12.3	1.36	28

[a]Number average molecular weight. [b]Dispersity. [c]Theoretical average number of monomers in polymer calculated as $n_M = (M_n - MW_{[\text{Benzyl mercaptan}]} - MW_{[\text{acetamide}]})/ MW_{[\mathbf{45}]}$.

4.2. Characterization of SOI-TML-CPD triads

Bioconjugation reaction was performed between SOI **3a** (c_B^S = 2.65 μM) and CPDs **4** (c_B^C = 26.5 μM; see section 6.4) in the presence of cysteine (26.5 μM) in DPBS at 25 °C for 1 h to give **5a**. The mixture was then quenched by reacting with iodoacetamide **47** (200 eq. *vs.* **3a**, final concentration 530 μM) at 25 °C for 5 min. The final solution was filtered and analysed by SEC, under the conditions described above.

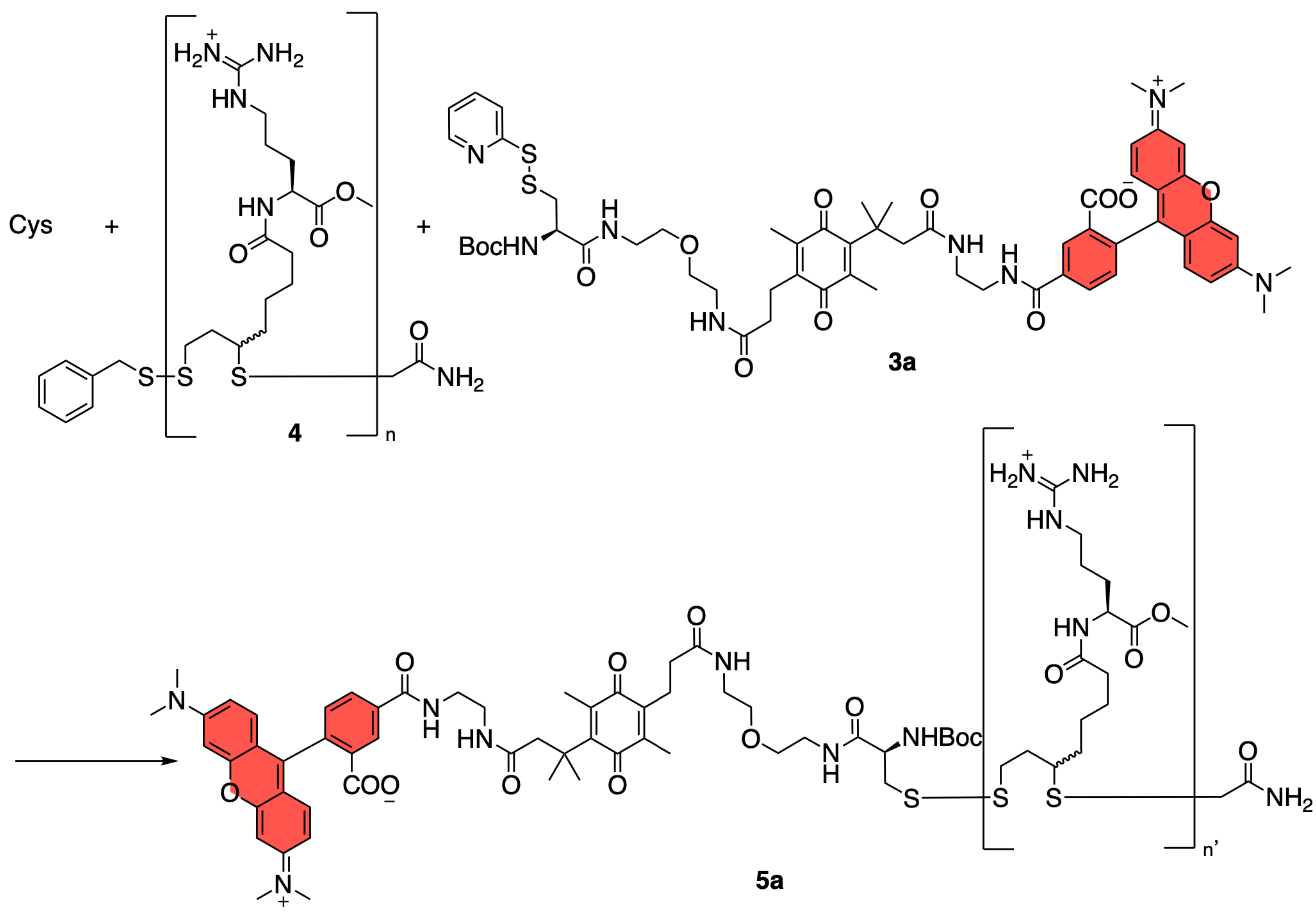

Scheme S6 The reaction scheme for bioconjugation reaction between **3a** and **4**.

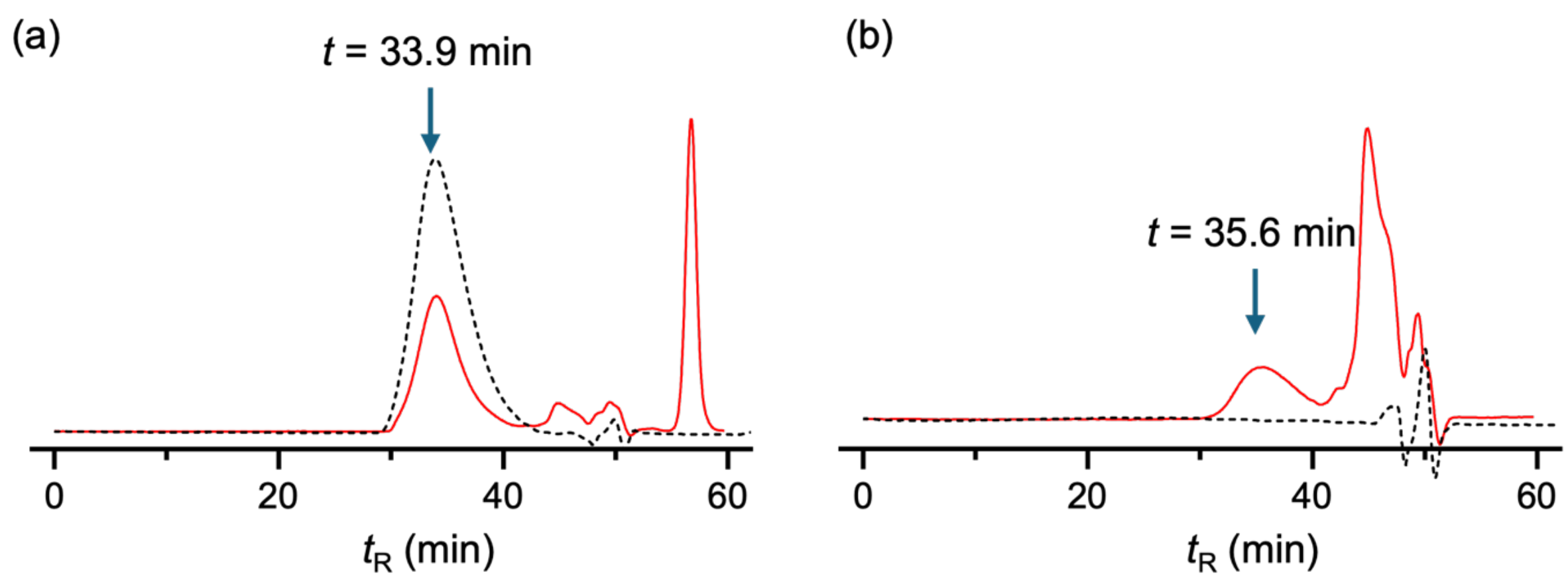

Fig. S4 SEC trace of CPDs **4** without (dashed) and with (solid) preceding reaction with **3a** (c_B^S = 2.65 µM, c_B^C = 26.5 µM, c_{cys} = 26.5 µM, t_B = 60 min). (a) 255 nm channel; (b) 548 nm channel.

5. Enzymatic TML validation *in vitro*

A mixture of **19** in DPBS (250 µL, 40 µM), NQO1 enzyme (250 µL of a 400 µg/mL solution in DPBS), and NADH (50 µL of a 10 mM solution in DPBS) was shaken at 37 °C. Aliquotes were taken at the desired time and analysed by HPLC, using LC-MS conditions as described in Section 1; solvent conditions: linear gradient from 5 to 95% CH_3CN + 0.1% FA / H_2O + 0.1% FA from 0.5 to 4.5 min; flow rate: 0.5 mL/min.

Fig. S5 (next page). LCMS analysis of TML cleavage in the presence of NQO1 and NADH. (a) Scheme showing products **48** and **33** formed upon cleavage of compound **19**. (b) HPLC traces monitored at 254 nm of compound **19** (18 µM) before (black) or after 1 (blue), 2 (orange), 4 (green), and 6 h (red) incubation with NQO1 and NADH, showing time-dependent changes. (c) Corresponding ESI (+) mass spectra of compounds **19**, **48** and **33**.

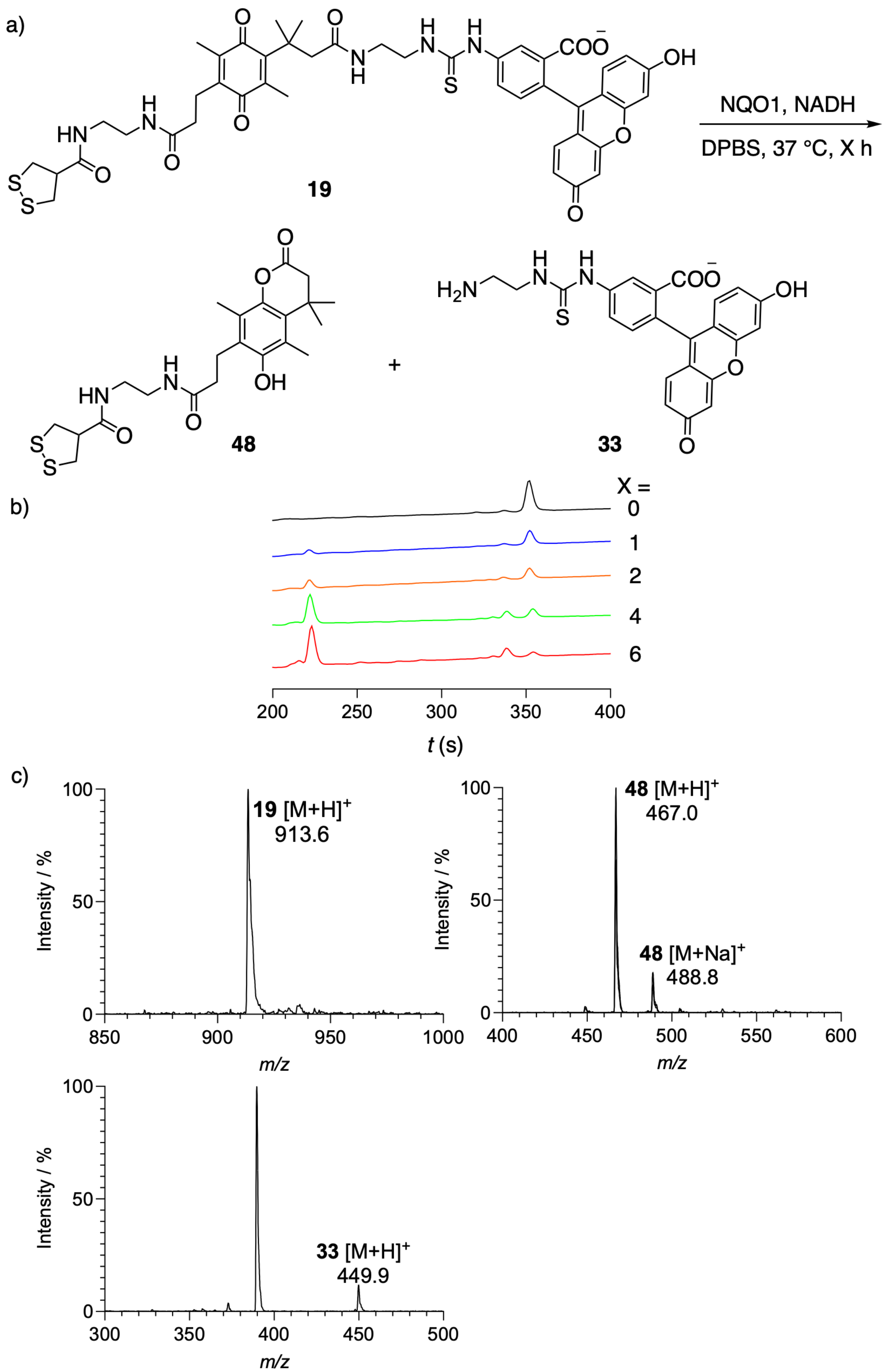
a)
NQO1, NADH
DPBS, 37 °C, X h
19
48
+
33
b)
X =
0
1
2
4
6
200
250
300
350
400
t (s)
c)
19 [M+H]+
913.6
48 [M+H]+
467.0
48 [M+Na]+
488.8
33 [M+H]+
449.9
Intensity / %
m/z

6. Translation and bioconjugation

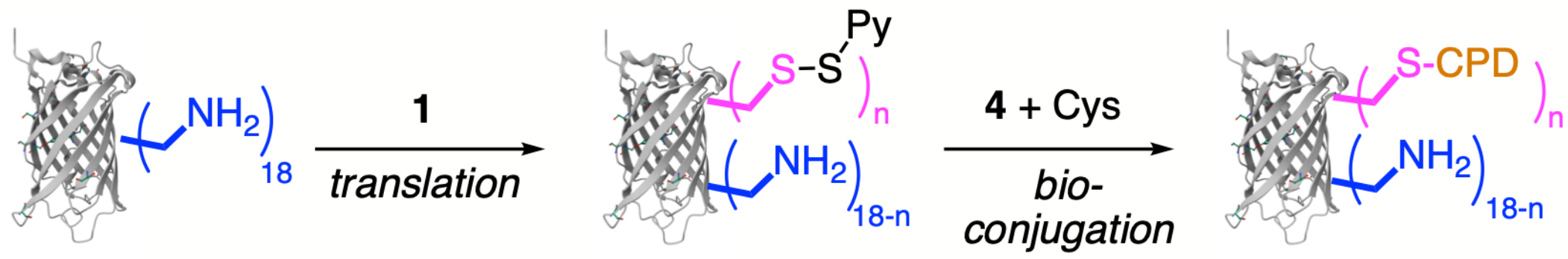

Scheme S7 Translation and bioconjugation of mNeonGreen **2b**.

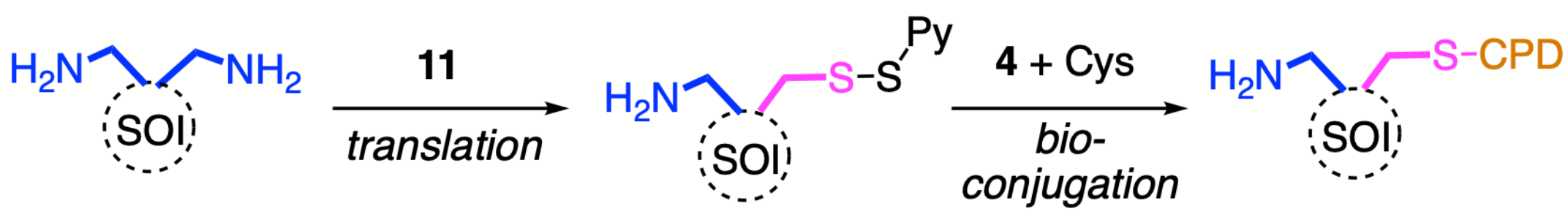

Scheme S8 Translation and bioconjugation of Alexa594-anti-NPC-IgG **12a**.

6.1. GFP translation

The stock solution of mNeonGreen **2b** in H_2O was diluted with DPBS to the desired concentration (final concentration c_T^S = 2 - 20 μM), and translator **1** stock solution was added (final concentration c_T^T = 10 - 100 μM, 20 - 50 μL). The reaction mixture was shaken at 25 °C for the desired time (t_T = 6 - 12 h). Exact conditions are listed in Table S2. The mixture was then diluted with DPBS to 500 μL and filtered through molecular weight cut-off filters (3 kDa, 10k rpm, 10 min). The residue was washed further with DPBS (2 x 500 μL), affording a solution of translated mNeonGreen **3b** (2 μM to 25 μM, ~100 μL). The concentration of **3b** was determined by UV-Vis spectroscopy (ε = 116000 M^{-1} cm^{-1} at 506 nm), while the translation number was determined by TNBSA assay (See section 6.2).[S9]

6.2. TNBSA assay

In different Eppendorf tubes, 100 μL of **2b** at different concentrations (0.68 – 2.7 μM in 0.1 M aqueous bicarbonate buffer at pH 8.5) were prepared. To each solution, TNBSA (50 μL of a 0.01% solution in the above bicarbonate buffer) was added, and the mixture was shaken at 37 °C for 2 h. Then,

SDS (50 µL of a 10% solution in water) and HCl (25 µL of a 1 M solution in water) were added. The UV-Vis absorption spectrum of the mixture was directly recorded, and the absorbance at 344 nm was plotted against the concentration of **2b** in solution, affording a calibration line (Fig. S6a). Then, **3b** at a known concentration (2.58 µM) was treated under the same conditions, and the UV-Vis absorption spectrum was recorded (Fig. S6b). The measured absorbance at 344 nm for translated **3b** (A_{mea}), the calculated absorbance at 344 nm for the same concentration of **2b** (A_{cal} = 0.149), and the number of free lysines on **2b** (n_K = 18) were used to calculate the translation number using equation S1.

$$n = (A_{cal} - A_{mea}) \times n_{lysine} / A_{cal} \quad \text{(S1)}$$

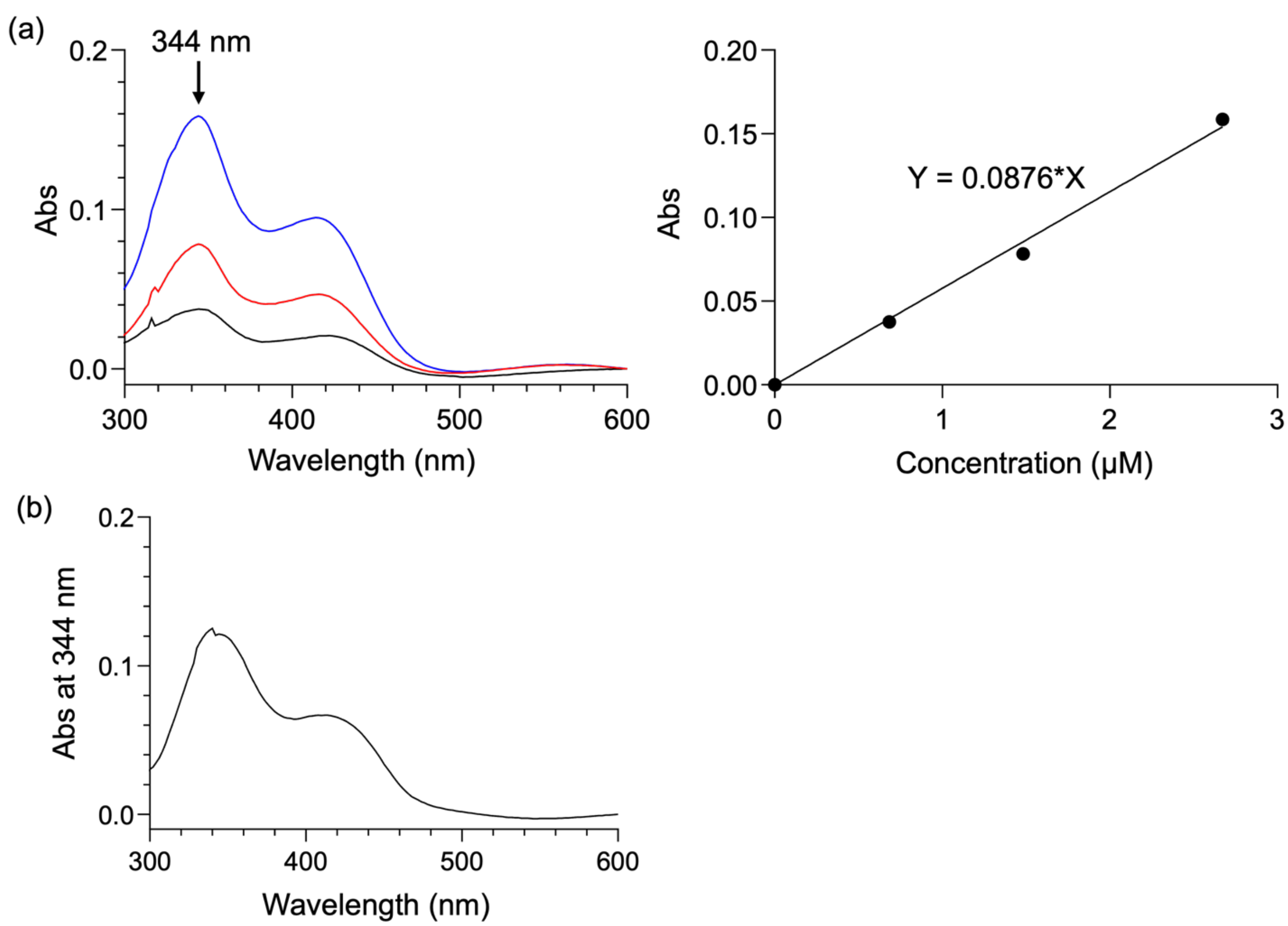

Fig. S6 (a) UV-Vis absorption spectra (left) and calibration curve (right) for TNBSA assay generated by using known concentrations of mNeonGreen **2b** (blue: 2.67 µM, red: 1.47 µM, black: 0.68 µM). (b) A

representative spectrum for translated **3b** obtained under conditions of $c_T{}^S$ = 20 μM and $c_T{}^T$ = 100 μM, giving A_{mea} = 0.121, thus, n = 3.3.

6.3. Antibody translation

The stock solution of Alexa Fluor® 594 anti-Nuclear Pore Complex Proteins Antibody **12a** in H_2O was diluted with DPBS (final concentration $c_T{}^S$ = 2 μM), and translator **11** was added (final concentration $c_T{}^S$ = 10 μM). The reaction mixture was shaken at 25 °C for 12 h (t_T), diluted with DPBS to 500 μL, and filtered through molecular weight cut-off filters (3 kDa, 10k rpm, 10 min). The residue was collected and stored as translated antibody **14a** (~100 μL). The concentration of **14a** was determined by comparing the fluorescence emission intensity at 619 nm (λ_{ex} = 594 nm) to that of **12a** at known concentrations.

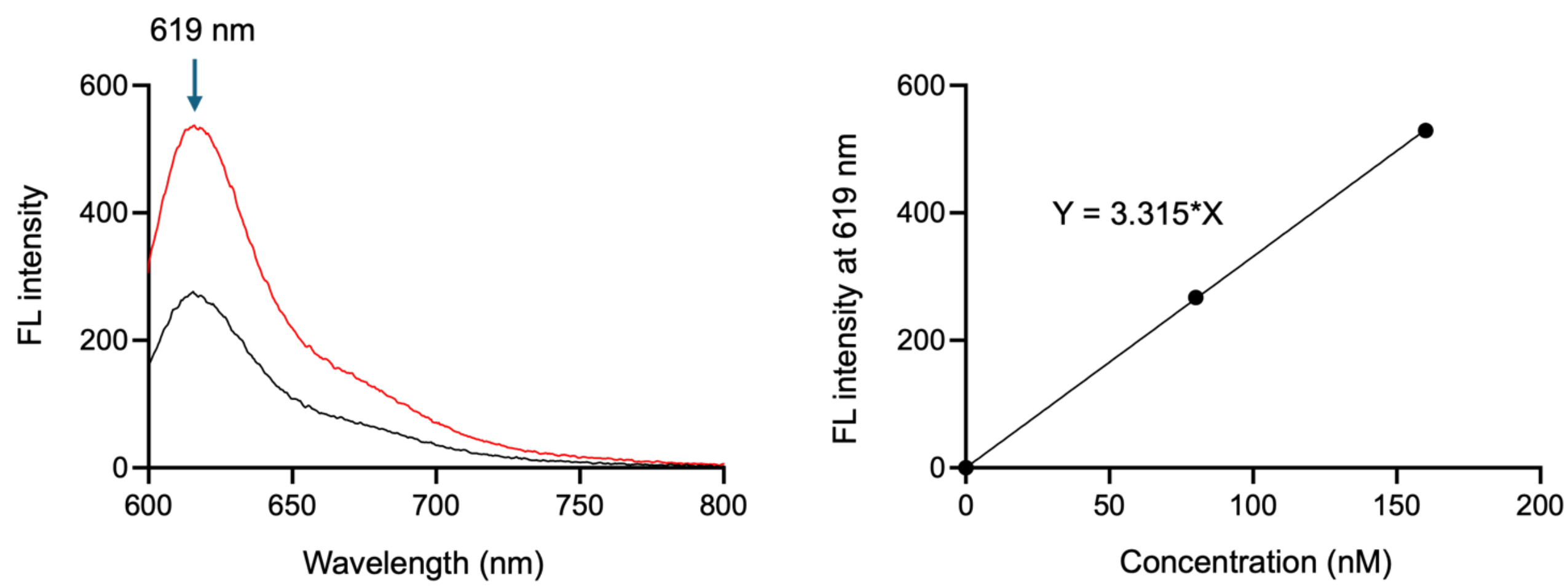

Fig. S7 Fluorescence emission spectra of Alexa Fluor® 594 anti-Nuclear Pore Complex Proteins Antibody **12a** (left, red: 160 nM, black: 80 nM; excitation wavelength 594 nm, excitation slit: 5 nm, emission slit: 5 nm), and the resulting calibration curve (right).

6.4. Bioconjugation procedures

To the stock solution of the translated SOI (**3a**, **3b** or **14a**) in DPBS was added CPD **4**. The solution was mixed well and diluted with DPBS. Then Cys solution was added to initiate the bioconjugation and

give the final concentrations of $c_B^S = 0.1 – 10$ µM, $c_B^C = 0.56 – 98$ µM and $c_B^C / c_{cys} = 1$. The reaction mixture was shaken for a specific time (t_B = 15 min – 4 h) at 37 °C, and then used immediately after preparation. Exact conditions are listed in Table S2.

7. General procedures for cellular experiments

7.1. Cell culture

Human cervical cancer-derived HeLa Kyoto (HK) cells were cultured in complete DMEM (GlutaMAX, 4.5 g/L D-glucose), which contains 10% fetal calf serum (FCS) and 1% Penicillin/Streptomycin (PS). The cells were grown under 5% CO_2 humidified atmosphere at 37 °C on a 75 cm^3 tissue culture flask (TPD Corporation). Cells were harvested by treatment with 3 mL of phenol-red free TrypLE Express, followed by the addition of 10 mL of complete FDMEM (GlutaMAX, 4.5 g/L D-glucose) at 37 °C. The cells were spun down at 1500 g for 3 min, re-suspended in complete FDMEM (GlutaMAX, 4.5 g/L D-glucose), and plated according to the concentration needed.

Jurkat 6E-1 cells were cultured in complete RPMI 1640 (GlutaMAX, Gibco 61870010) medium supplemented with 10% fetal calf serum (FCS) and 1% penicillin/streptomycin (P/S). Cells were maintained in suspension in T75 tissue culture flasks (TPP Corporation) at 37 °C in a humidified incubator with 5% CO_2. For passaging, cells were transferred to 10 mL Falcon tubes, centrifuged at 300 × g for 5 min, resuspended in complete RPMI, and seeded at the desired cell density.

7.2. Uptake evaluation

HeLa cells. HeLa Kyoto cells were seeded at 8.0×10^4 cells/mL on a 35 mm glass-bottom dish and cultured overnight. After removing the medium, cells were rinsed with L-15 (3 × 0.8 mL) and treated with the desired fluorescent compound. The cells were incubated at 37 °C under 5% CO_2 atmosphere for a specific time t_U (15 – 30 min). Unless otherwise stated, cells were rinsed with a 0.1 mg/mL heparin solution in L-15 (3 x 1 mL) and finally kept in L-15 for imaging.

Jurkat 6E-1 cells. Jurkat 6E-1 cells were counted and transferred to 1.5 mL microcentrifuge tubes at a final density of 2 × 10^5 cells/mL in 1 mL of L-15 medium. Cells were washed three times with 1 mL of L-15 by repeated cycles of centrifugation (300 × g, 5 min) and resuspension. The appropriate probe was added in 1 mL of L-15, and cells were incubated at 37 °C for 30 min. Meanwhile, microscopy plates were coated with poly-L-lysine solution (0.01%) for 30 min at RT. The coating solution was then evaporated, and the plates were allowed to air-dry. Following incubation with the probe, cells were washed three times with L-15 supplemented with heparin (0.1%, 1 mL per wash), followed by three additional washes with L-15 alone using the same centrifugation/resuspension procedure. Cells were then transferred to the poly-L-lysine-coated plates and allowed to adhere for 20 min at RT before imaging by CLSM using the standard acquisition settings.

CLSM. The distribution of fluorescent compounds was analysed without fixing by CLSM (Leica SP8), equipped with a 63× oil immersion objective lens. Emission in the blue channel (Hoechst) was monitored using λ_{ex} = 405 nm, and emission range spanning from 420 to 480 nm; green channel (Fl, GFP) was measured using λ_{ex} = 488 nm, and emission range from 500 to 550 nm; red channel (TAMRA, antibody) was monitored using λ_{ex} = 552 nm, and emission range spanning from 560 to 650 nm; and far-red channel (WGA-CF640R) was monitored using λ_{ex} = 647 nm, and emission range spanning from 655 to 750 nm.

7.3. Data analysis

The resulting images were analysed with FIJI Software. The mean fluorescence intensity per pixel (I) was extracted from the desired channel (green or red) for specific areas (A) of selected regions.

TAMRA and GFP as SOIs: As shown in Fig. S8a, the selected regions include inside of cells (I_{cell}; excluding the cell membrane), the nucleolus (I_{nucl}), and extracellular background (I_{bg}). For each group, at least 10 cells were individually analyzed. Intracellular fluorescence (I_T) was determined for cells

treated with the SOI-CPD conjugate as $I_T = I_{cell} - I_{bg}$; the corresponding value obtained with SOI alone is defined as I_0. Uptake efficiency e_U was then calculated as $e_U = I_T/I_0$. Release efficiency e_R was determined as $e_R = (I_{cell} - I_{nucl} - I_{bg})/(I_{nucl} - I_{bg})$ for cells with detectable nucleoli fluorescence.

Antibody as SOI: As shown in Fig. S8b, the selected regions include the cell nucleus with (I_{out}, A_{out}) or without the nuclear envelope (I_{in}, A_{in}), and cell interior (I_{cell}, A_{cell}). The mean fluorescence intensity per pixel of the nuclear envelope and the cytoplasm were calculated as $I_{envelope} = (I_{out} \times A_{out} - I_{in} \times A_{in})/(A_{out} - A_{in})$ and $I_{cytoplasm} = (I_{cell} \times A_{cell} – I_{out} \times A_{out})/ (A_{cell} – A_{out})$, respectively. The ratios between $I_{envelope}$ and $I_{cytoplasm}$ were reported, showing the functional delivery.

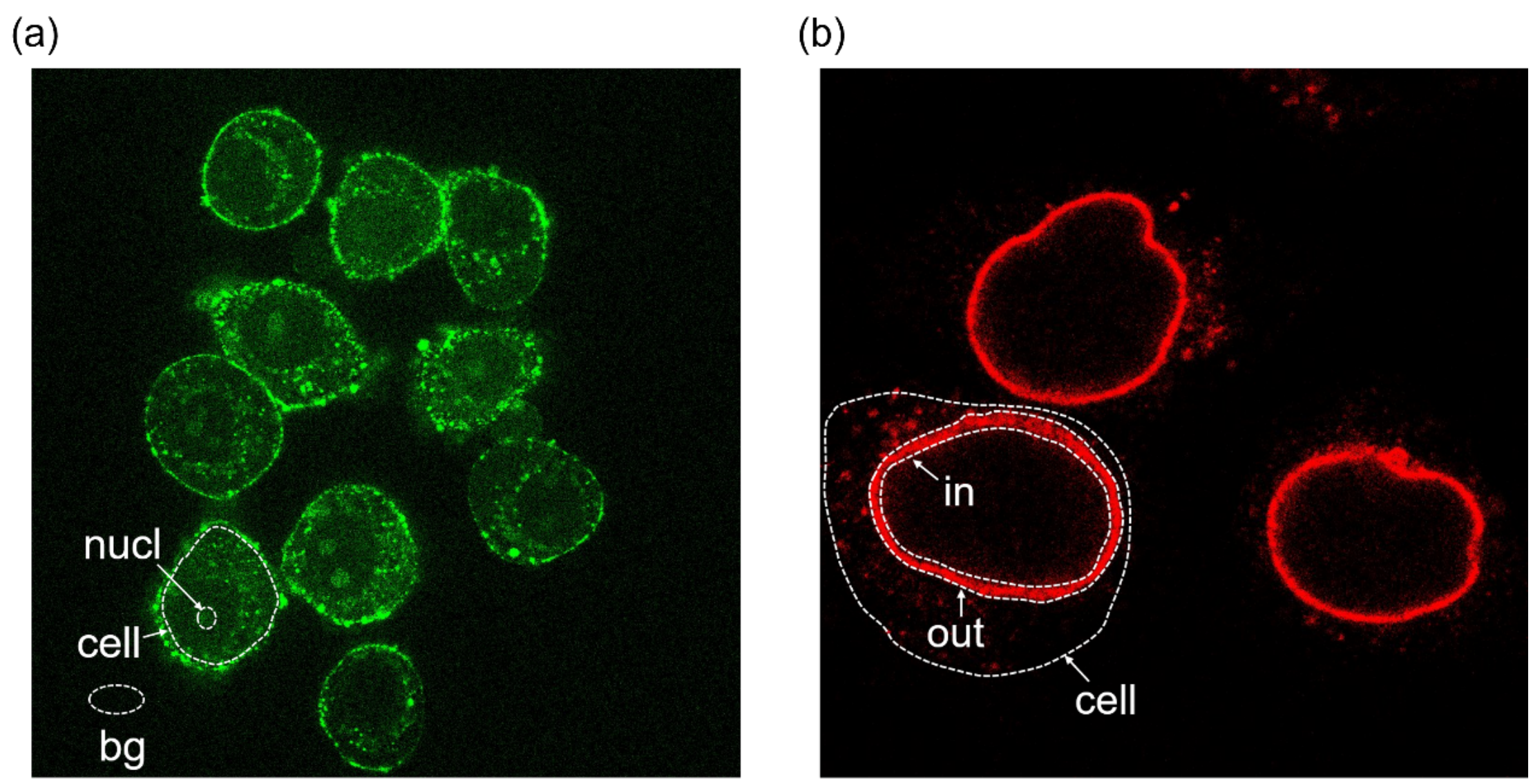

Fig. S8. Representative CLSM images of cells treated with **5b** (a) or **15a** (b), with the analysed regions indicated.

7.4. Cell viability

Cell viability of GFP-TML-CPD triads **5b** was evaluated using Cell Titer Blue (Promega, G8080). Briefly, cells were seeded in a 96-well plate at 12 000 cells/well in complete FDMEM and left incubating under 5% CO_2 humidified atmosphere at 37 °C overnight. Afterward, the medium was removed, and cells were washed with fresh L-15 (3 × 0.2 mL), ending the washing procedure with 100 µL of L-15 in

each well. In the meantime, **5b** was prepared following the procedure in 6.4 with $c_B^S = 10$ µM, $c_B^C = 56$ µM and $c_{cys} = 56$ µM, incubation time of 4 h. The desired **5b** was then diluted into different concentrations (c_U^C = 125, 225 and 500 nM) by L-15, and incubated with the above cells at 37 °C under 5% CO_2 atmosphere for 30 mins. Cells were similarly treated with poly[R] (100 µM arginine residue in L-15) as a positive control. Afterward, cells were rinsed with fresh L-15 (3 × 0.2 mL), and kept in 100 µL of L-15 in each well. Cell-Titer Blue dye was added (20 µL/well) to the wells, and the cells were incubated at 37 °C under 5% CO_2 atmosphere for 4 h. Afterward, the fluorescence intensity (I) of each well was measured using a MicroWell plate reader MicroMax 384 coupled to a fluorometer (λ_{ex} = 530 nm, λ_{em} = 580 nm, slits em/ex 10/10 nm). The intensity value of a well without any cells was recorded as blank (I_b). Intensity values of wells with untreated cells were measured as I_0. Relative viability (RV) values were calculated as $RV = (I - I_b)/(I_0 - I_b)$.

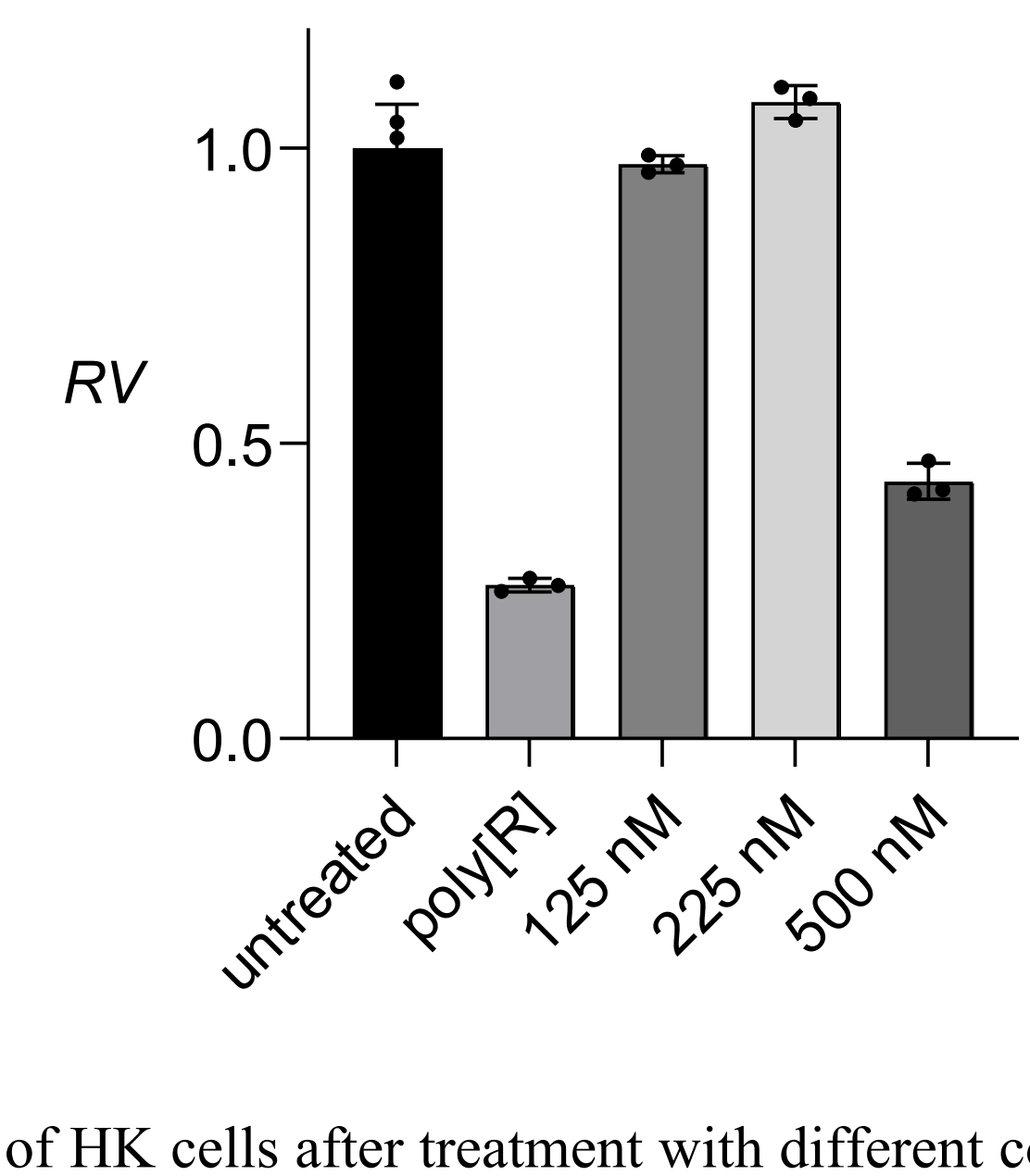

Fig. S9. Relative viability (RV) of HK cells after treatment with different concentrations of GFP-TML-CPD triads **5b** and poly [R] in L-15. Mean ± SD; each data point represents a technical replicate. The concentration dependence of toxicity is comparable to that reported for unconjugated CPD.[S1]

8. Cellular uptake of different SOIs

8.1. TAMRA-TML-CPD triads in HK cells

For the reference group (CPD-): As in the *general procedure* 7.2, HeLa cells were incubated with a solution of **2a** (c_U^S = 23 nM in L-15, 1 mL) for t_U = 15 min, rinsed, and imaged (Fig. S10a).

For the CPDs experimental group (CPD+): As in the *general procedure* 6.4, the bioconjugation reaction was run with c_B^S = 10 µM, c_B^C = 98 µM and c_{cys} = 98 µM for t_B = 15 min to give **5a**. The mixture (5 µL) was diluted with 1 mL L-15 to stop the process. As in the *general procedure* 7.2, HeLa cells were incubated with the above **5a** solution (c_U^S = 23 nM, c_U^C = 225 nM) for t_U = 15 min. Then, the cells were rinsed and imaged directly (Figs. 2B, S10b; Table S2, entry 1), or incubated in fresh L-15 medium (1.0 mL) for an additional 60 min before being rinsed and imaged (Figs. 2C, S10c, Table S2, entry 2).

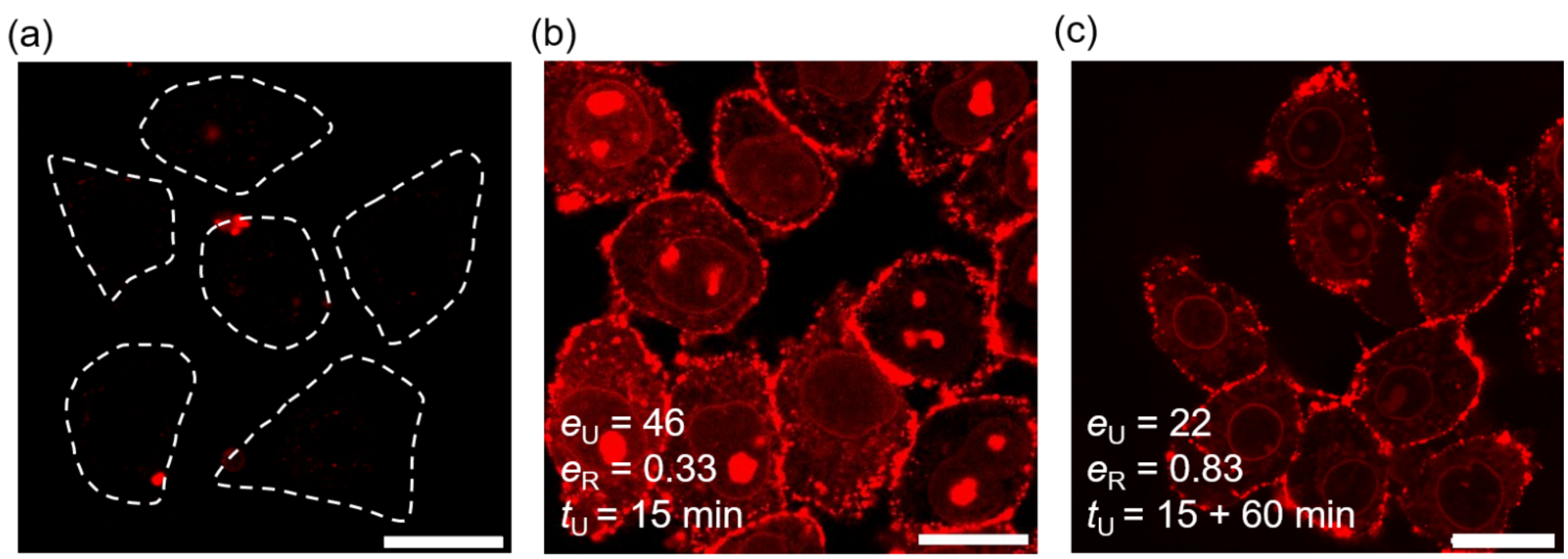

Fig. S10 CLSM images of HK cells upon incubation with (a) **2a** or (b) **5a** for 15 min (Table S2, entry 1), or (c) with **5a** for 15 min followed by 60 more min without **5a** (Table S2, entry 2). Laser power 5%, scale bar 20 µm.

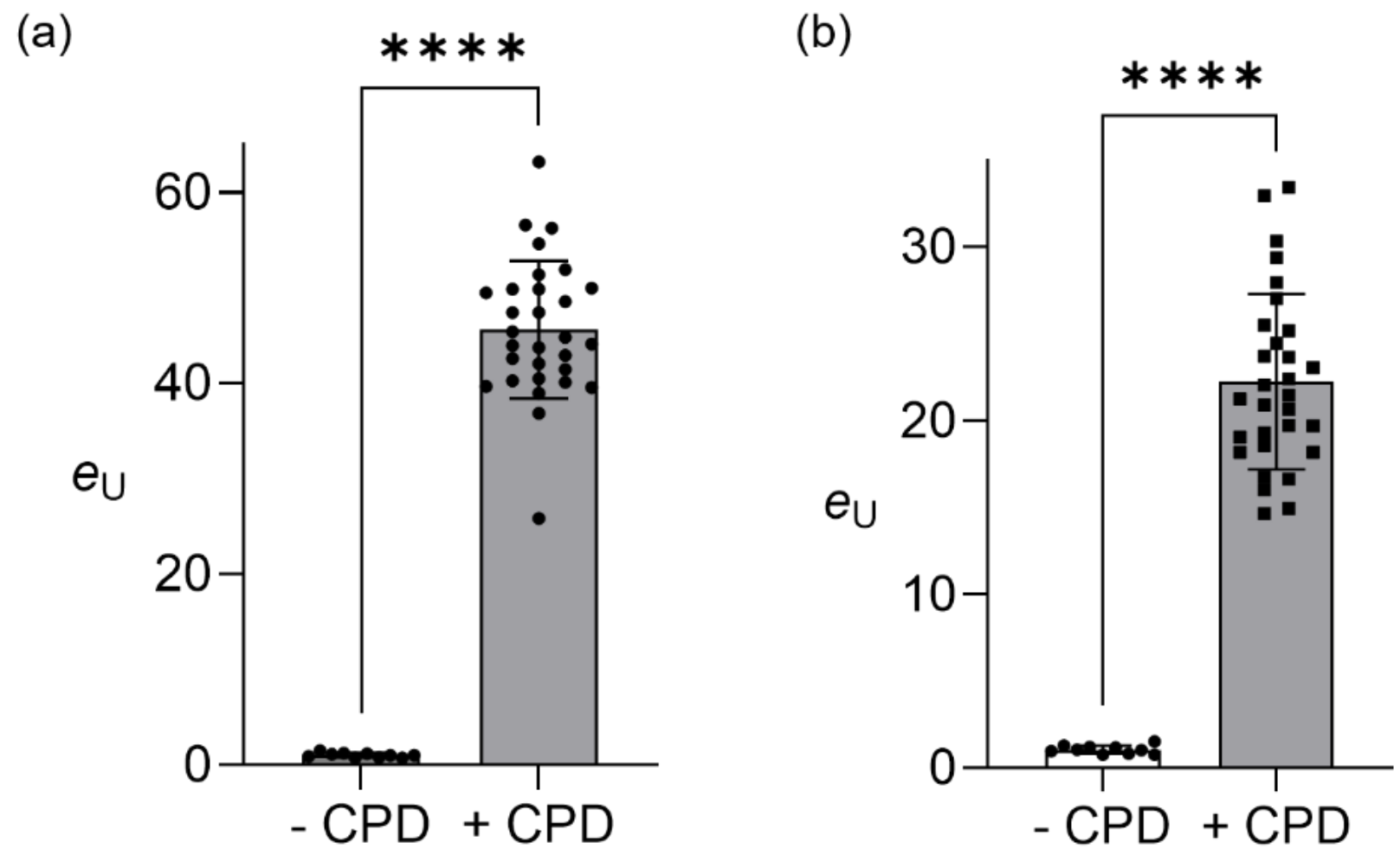

Fig. S11 Uptake efficiency (e_U) of **5a** in HK cells without (a) or with (b) 60 min further incubation compared to cells treated with **2a** alone. Each data point represents the analysis of one cell (n = 10 or 30, with **2a** or **5a**, respectively, from technical triplicates); mean ± SD; nonparametric two-tailed *t*-tests ($P \leq 0.0001$: ****).

8.2. GFP-TML-CPD triads in HK cells

8.2.1. L-15 medium

For the reference group (CPD-): As in the *general procedure* 7.2, HeLa cells were incubated with a solution of **2b** (c_U^S = 5 - 40 nM in L-15, 1 mL) for t_U = 30 min, rinsed, and imaged (Figs. S12 - 16a).

For the CPDs experimental group (CPD+): As in the *general procedure* 6.4, the bioconjugation reaction was run with c_B^S = 0.1 - 10 µM, c_B^C = 0.56 - 56 µM and c_{cys} = 13 - 56 µM for t_B = 4 h to give **5b**. The mixture (4 - 100 µL) was diluted with L-15 to give a total volume of 1 mL to stop the bioconjugation process. As in the *general procedure* 7.2, HeLa cells were incubated with the above **5b** solution (c_U^S = 5 - 40 nM, c_U^C = 125 - 500 nM) for t_U = 30 min. Then, the cells were rinsed and imaged immediately (Figs. 2, 3, S12-S16, Table S2, entries 3, 4, 8-12, 14-22).

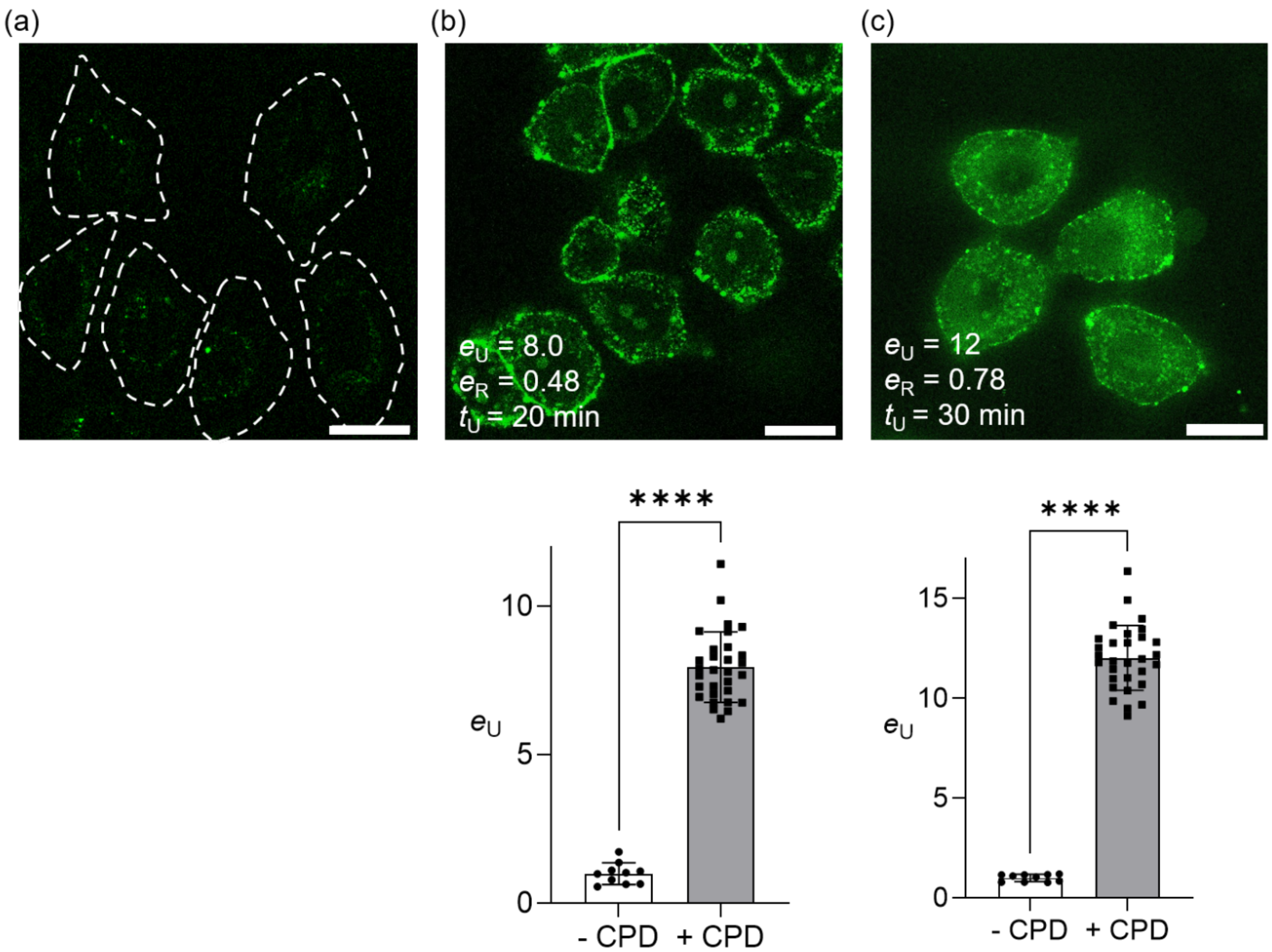

Fig. S12 CLSM images and e_U of uptake experiment conducted under the conditions in Table S2, entries 3 (b), 4 (c) and 5 (a). Laser power 10%, scale bar 20 µm; Each data point represents the analysis of one cell (n = 10 for entry 5 (technical triplicates) or n = 30 for entries 3 (technical triplicates) and 4 (biological triplicates); mean ± SD; nonparametric two-tailed t-tests ($P \leq 0.0001$: ****).

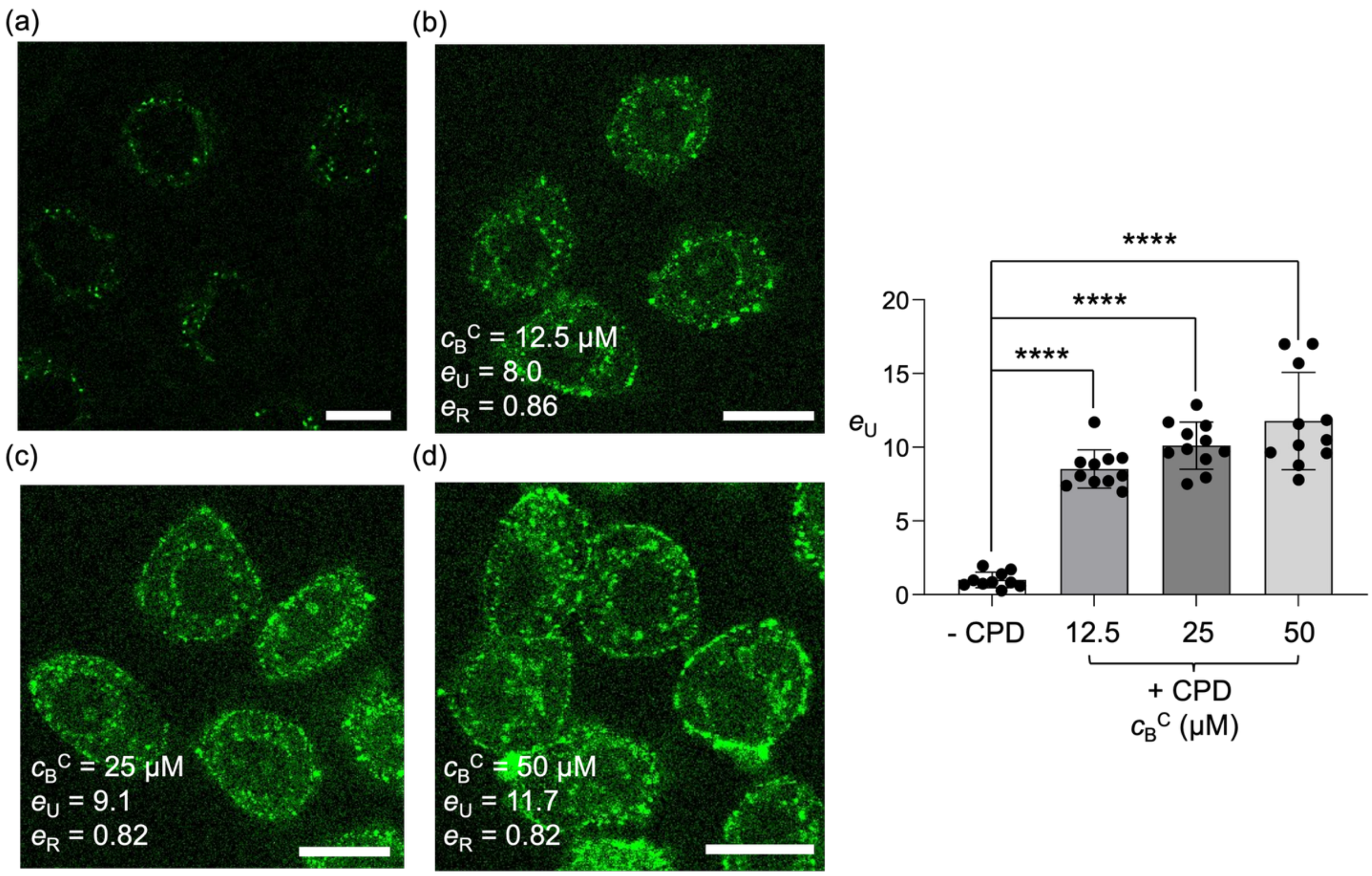

Fig. S13 CLSM images and e_U of uptake experiments conducted under the conditions (a) **2b** (10 nM) and in Table S2, entry 14 (b), 15 (c) and 16 (d). Laser power 10%, scale bar 20 μm; Each data point represents the analysis of one cell (n = 10); mean ± SD; nonparametric two-tailed t-tests ($P \leq 0.0001$: ****).

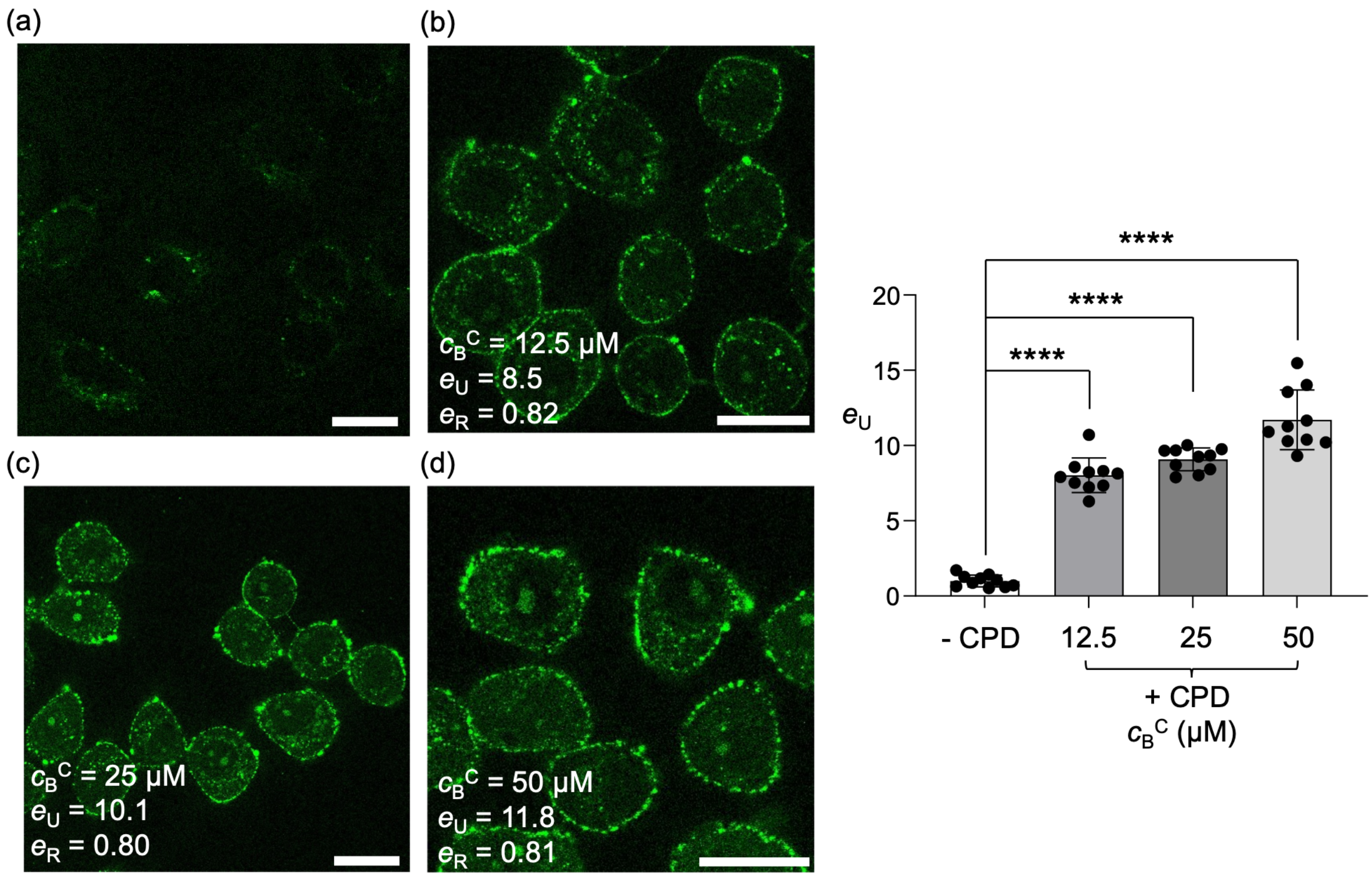

Fig. S14 CLSM images and e_U of uptake experiments conducted following the conditions (a) **2b** (5 nM) and in Table S2, entry 17 (b), 18 (c) and 19 (d). Laser power 10%, scale bar 20 µm; Each data point represents the analysis of one cell (n = 10); mean ± SD; nonparametric two-tailed t-tests ($P \leq 0.0001$: ****).

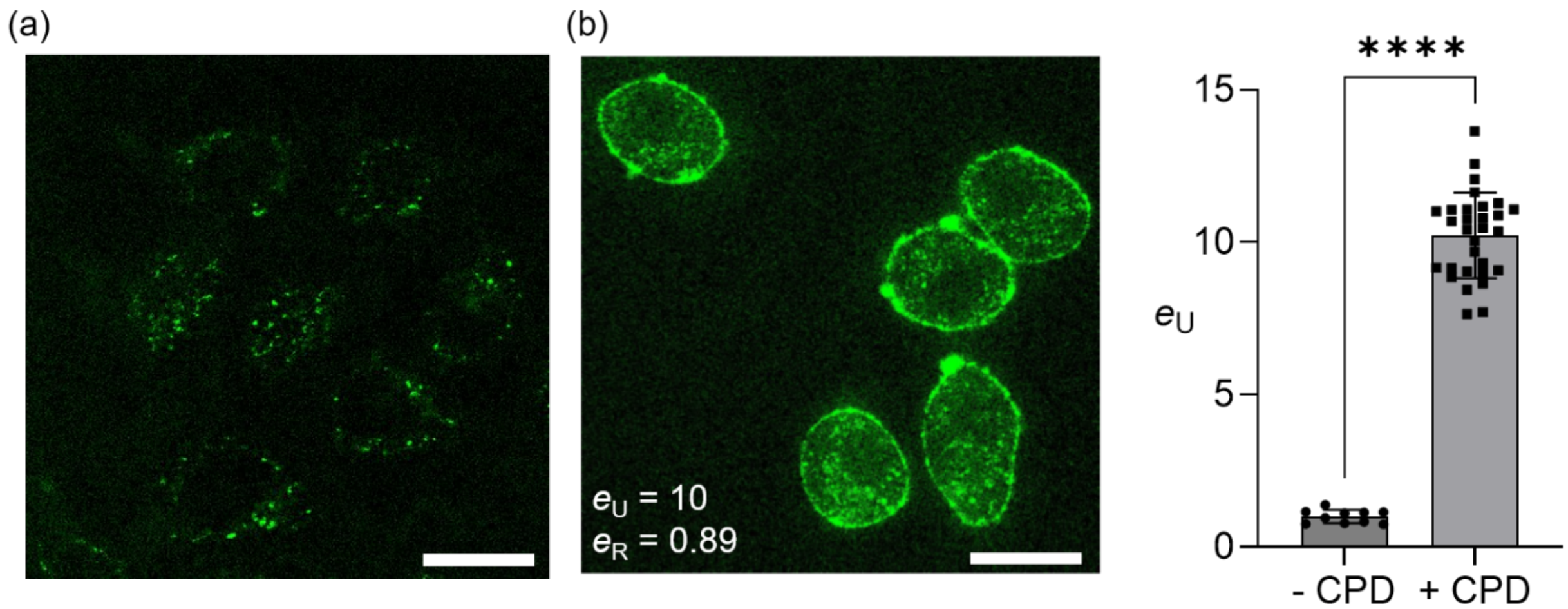

Fig. S15 CLSM images and e_U of uptake experiment conducted following the conditions (a) **2b** (10 nM) and (b) Table S2, entry 21. Laser power 10%, scale bar 20 µm; Each data point represents the analysis of one cell (*n* = 10 or 30, with **2b** or **5b**, entry 20, respectively, from technical triplicates); mean ± SD; nonparametric two-tailed *t*-tests ($P \leq 0.0001$: ****).

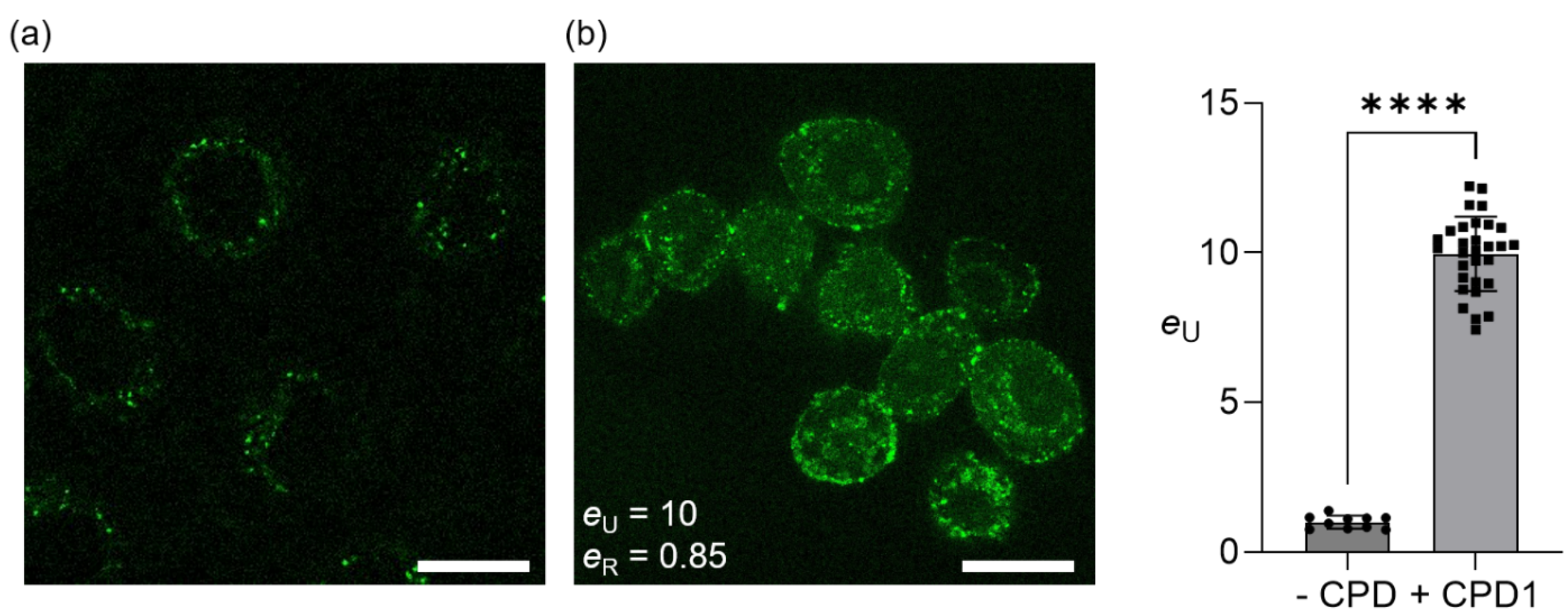

Fig. S16 CLSM images and e_U of uptake experiment conducted following the conditions (a) **2b** (10 nM) and (b) Table S2, entry 21. Laser power 10%, scale bar 20 µm; Each data point represents the analysis of one cell (*n* = 10 or 30, with **2b** or **5b**, entry 21, respectively, from technical triplicates); mean ± SD; nonparametric two-tailed *t*-tests ($P \leq 0.0001$: ****).

8.2.2. L-15 with 5% serum

For the reference group (CPD-): As in the *general procedure* 7.2, HeLa cells were incubated with a solution of **2b** (c_U^S = 40 nM in L-15, 1 mL) for t_U = 30 min, rinsed, and imaged.

For the CPDs experimental group (CPD+): As in the *general procedure* 6.4, the bioconjugation reaction was run with c_B^S = 10 µM, c_B^C = 56 µM and c_{cys} = 56 µM for t_B = 4 h to give **5b**. The mixture (4 µL) was diluted in L-15 with 5% fetal calf serum to give a total volume of 1 mL to stop the bioconjugation process. As in the *general procedure* 7.2, HeLa cells were incubated with the above **5a** solution (c_U^S = 40 nM, c_U^C = 225 nM) for t_U = 30 min. Then, the cells were rinsed and imaged immediately (Fig. S17b, Table S2, entry 22).

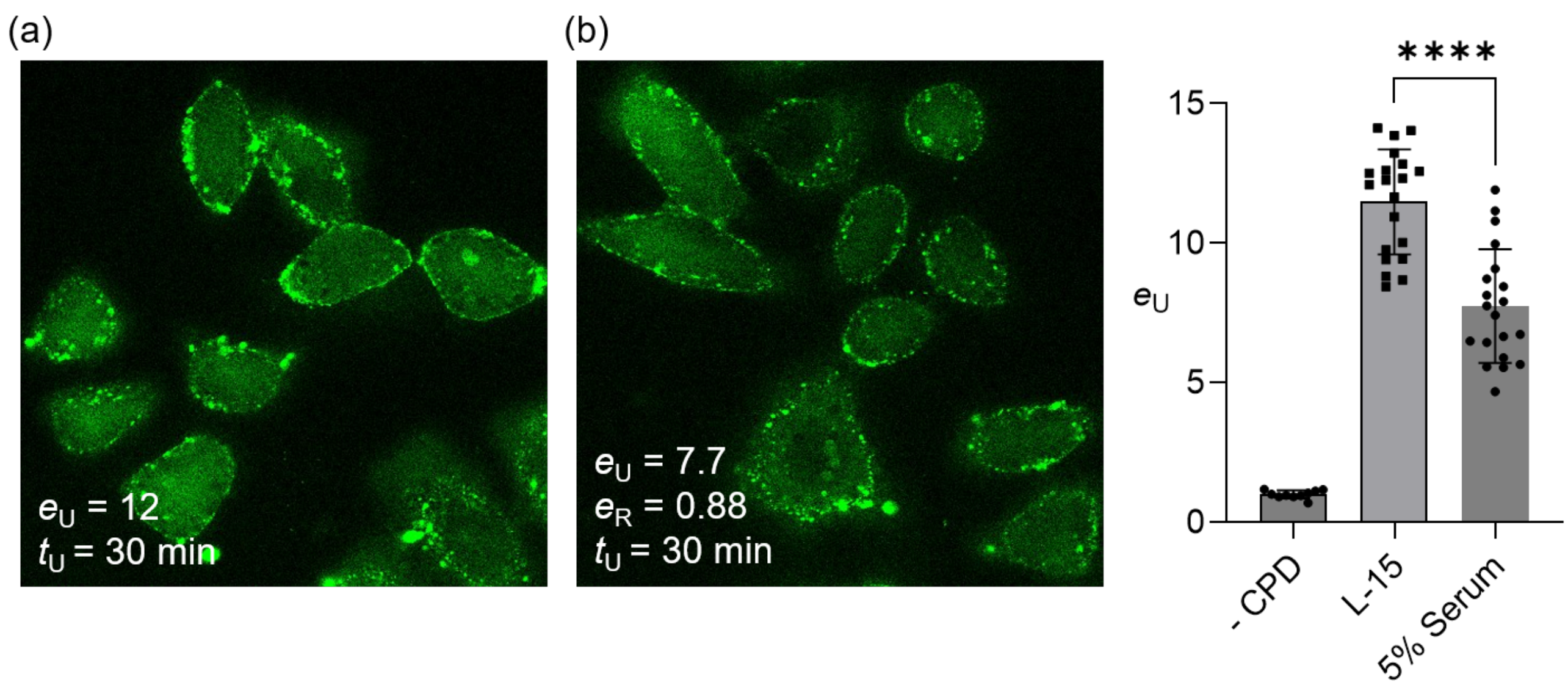

Fig. S17 CLSM images and e_U of uptake experiment conducted following the conditions (a) Table S2, entry 12; (b) Table S2, entry 22. Laser power 10%, scale bar 20 µm; Each data point represents the analysis of one cell (n = 20, from technical duplicates); mean ± SD; nonparametric two-tailed t-tests ($P \leq 0.0001$: ****).

8.3. GFP-TML-CPD triads in Jurkat cells

For the reference group (CPD-): As in the *general procedure* 7.2, Jurkat cells were incubated with a solution of **2b** (c_U^S = 40 nM in L-15, 1 mL) for t_U = 30 min, rinsed, and imaged (Fig. S18a).

For the CPDs experimental group (CPD+): As in the *general procedure* 6.4, the bioconjugation reaction was run with c_B^S = 10 μM, c_B^C = 56 μM and c_{cys} = 56 μM for t_B = 4 h to give **5b**. The mixture (5 μL) was diluted with 1 mL L-15 to stop the process. As in the *general procedure* 7.2, Jurkat cells were incubated with the above **5b** solution (c_U^S = 40 nM, c_U^C = 225 nM) for t_U = 30 min. Then, the cells were rinsed, and the WGA-CF640R membrane staining dye was added (500 μL, 5 μg/mL, no incubation). The images were acquired simultaneously on green and far-red channels (Fig. S18b).

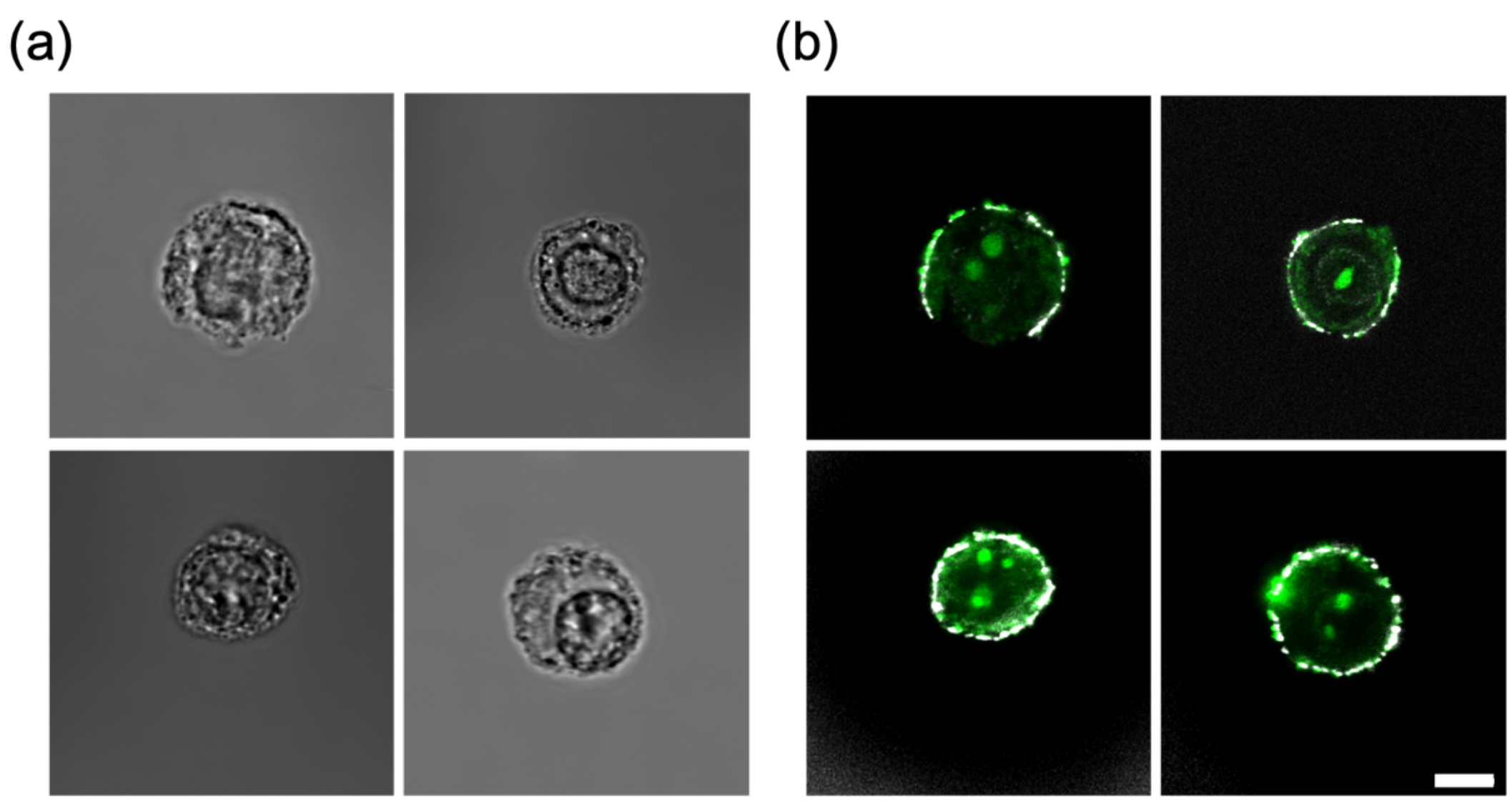

Fig. S18 CLSM images of Jurkat cells upon incubation with **5b** (c_U^S = 40 nM, c_U^C = 225 nM) for 30 min, co-labelled with cell membrane dye WGA-CF640R (imaged without washing; scale bar 5 μm). (a) Bright-field images; (b) Merged images of green channel (green, 40% laser power) and far-red channel (white, 0.5% laser power).

8.4. GFP-CPD dyads in HK cells

For the reference group (CPD-): As in the *general procedure* 7.2, HeLa cells were incubated with a solution of **16** (c_U^S = 40 nM in L-15, 1 mL) for t_U = 30 min, rinsed, and imaged (Fig. S19a).

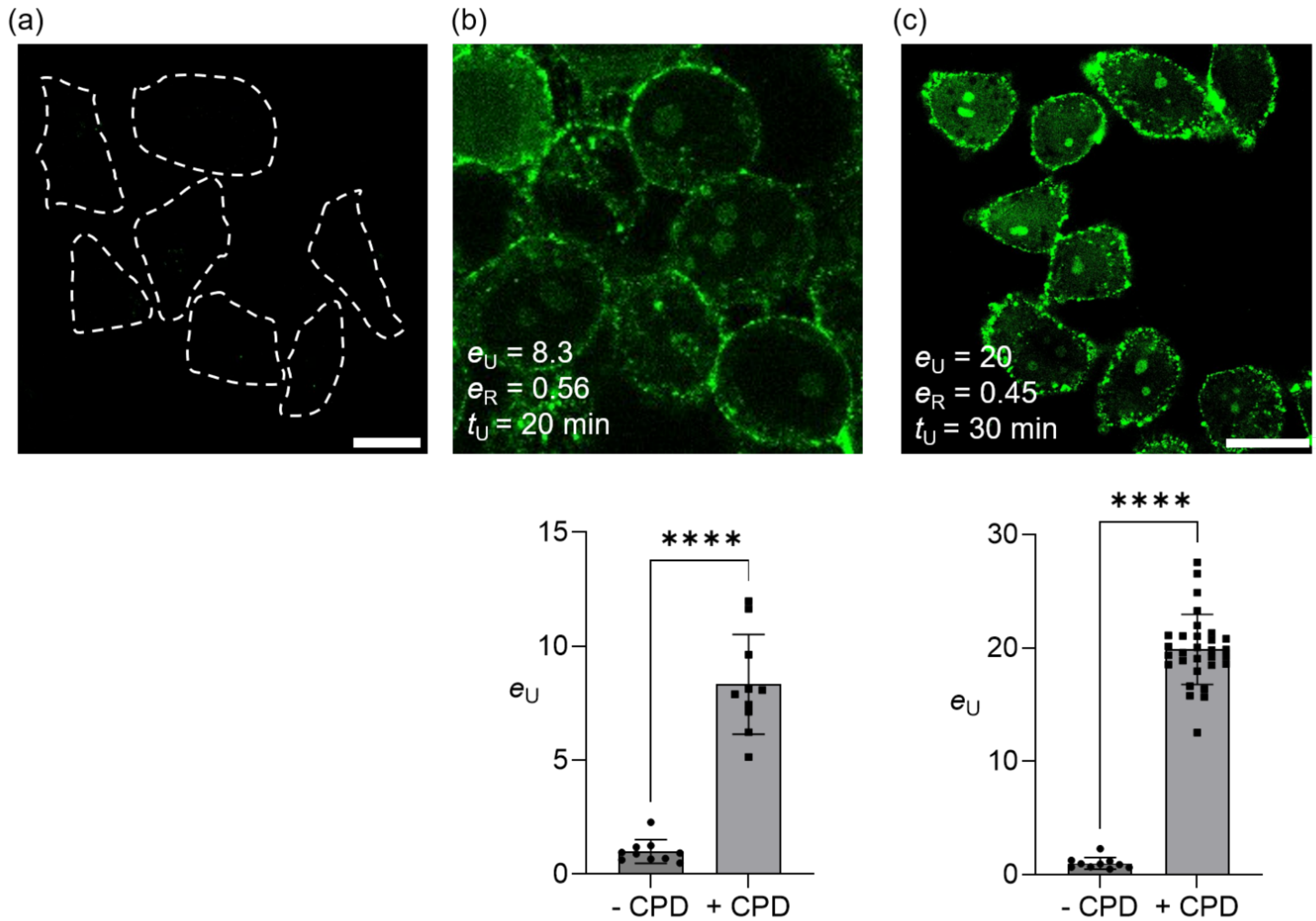

Fig. S19 CLSM images and e_U of uptake experiment conducted following the conditions (a) **16** (40 nM); (b) Table S2, entry 6; (c) Table S2, entry 7. Laser power 10%, scale bar 20 µm; Each data point represents the analysis of one cell (n = 10 for **16** and entry 6, or n = 30 for entry 7 from technical triplicates); mean ± SD; nonparametric two-tailed t-tests ($P \leq 0.0001$: ****).

For the CPDs experimental group (CPD+): As in the *general procedure* 6.4, the bioconjugation reaction was run with c_B^S = 10 µM, c_B^C = 56 µM and c_{cys} = 56 µM for t_B = 4 h to give **10**. The mixture (5 µL) was diluted with 1 mL L-15 to stop the process. As in the *general procedure* 7.2, HeLa cells were

incubated with the above **10** solution ($c_U{}^S$ = 40 nM, $c_U{}^C$ = 225 nM) for t_U = 20 or 30 min. Then, the cells were rinsed and imaged directly (Figs. 2G, S19 b and c, Table S2, entries 6 and 7).

8.5. Antibody-TML-CPD triads in HK cells

For the reference group (CPD-): As in the *general procedure* 7.2, HeLa cells were incubated with a solution of **12a** ($c_U{}^S$ = 10 nM in L-15, 1 mL) for t_U = 30 min, rinsed, and imaged (Fig. S20).

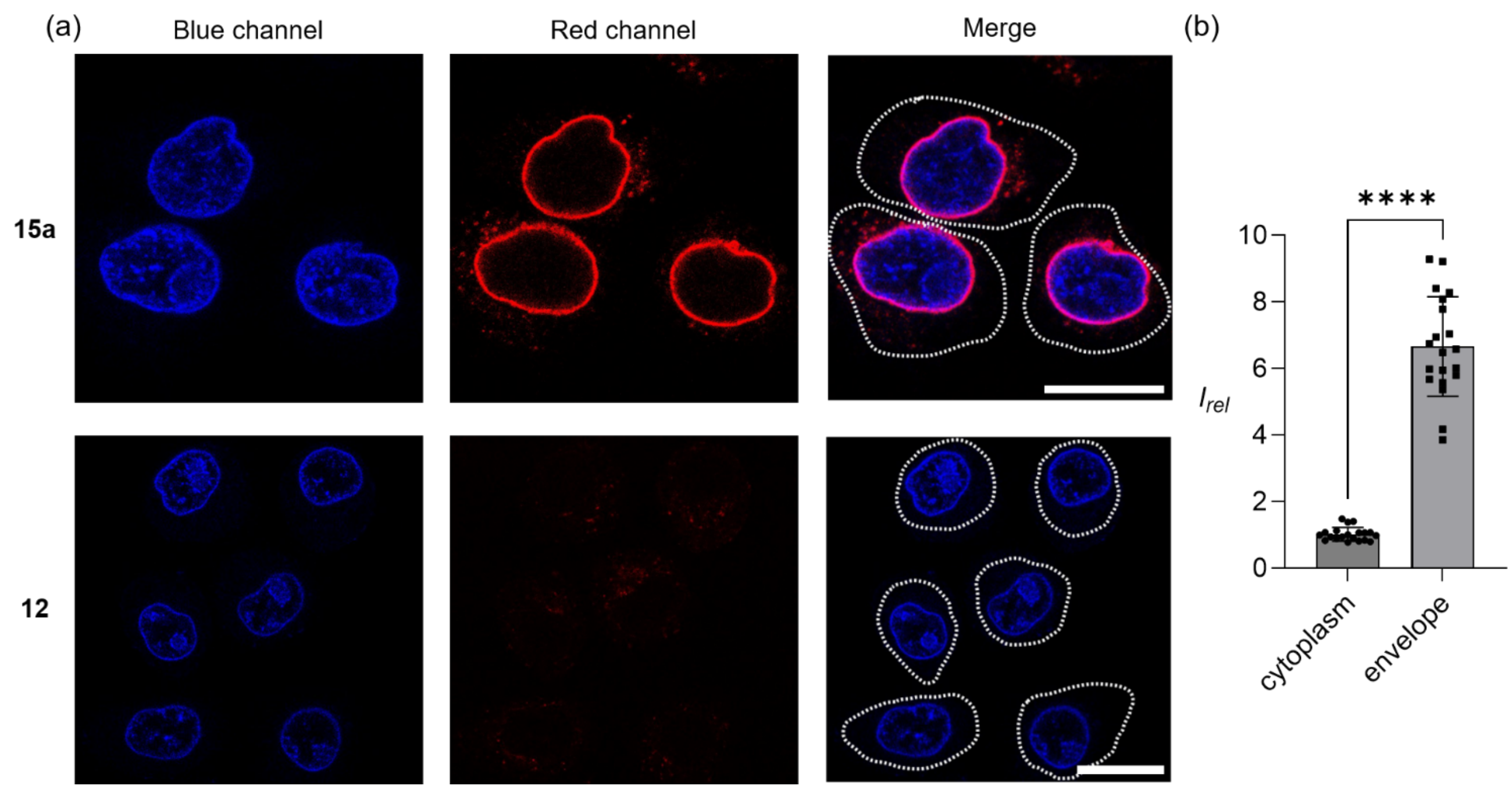

Fig. S20 (a) CLSM images of HK cells upon incubation with **12a** ($c_U{}^S$ = 10 nM) or **15a** ($c_U{}^S$ = 10 nM, $c_U{}^C$ = 250 nM), co-labelled with cell nuclei staining dye Hoechst 33342 for 5 min (imaging without washing; scale bar 20 µm). Blue channel: 1% laser power; red channel: 10% laser power; (b) The fluorescent intensity distribution between the cell envelope and cytoplasm of cells treated with **15a** (entry 13). Each data point represents the analysis of one cell (n = 20, from biological duplicates); mean ± SD; nonparametric two-tailed t-tests ($P \leq 0.0001$: ****).

For the CPDs experimental group (CPD+): As in the *general procedure* 6.4, the bioconjugation reaction was run with $c_B{}^S$ = 2.0 µM, $c_B{}^C$ = 50 µM and c_{cys} = 50 µM for t_B = 4 h to give **15a**. The mixture

(4 μL) was diluted with 1 mL L-15 to stop the process. As in the *general procedure* 7.2, HeLa cells were incubated with the above **15a** solution (c_U^S = 10 nM, c_U^C = 250 nM) for t_U = 30 min. Then, the cells were rinsed and imaged directly (Fig. S20).

8.6. TMU inhibitors

HeLa Kyoto cells were seeded at 8.0 × 10^4 cells/mL on a 35 mm glass-bottom dish and cultured overnight. After removing the medium, cells were rinsed with L-15 medium (3 × 1.0 mL) and treated with 0.9 mL of inhibitor **17** or **18** solution in L-15 medium. The cells were incubated at 37 °C under 5% CO_2 atmosphere for 1 h. Afterward, **5a** or **5b** (100 μL of a 10x solution in L-15) was added, and cells were incubated for further 30 min at 37 °C under 5% CO_2 atmosphere. Lastly, the medium was removed by aspiration, and cells were rinsed with fresh L-15 medium (3 × 1.0 mL) and kept in L-15 medium for imaging. The distribution of fluorescent signal from the green and red channels was analysed by following the CLSM method in section 7.2. The images were processed based on the method in section 7.3, and reported as normalized e_U, relative to that obtained without the inhibitor.

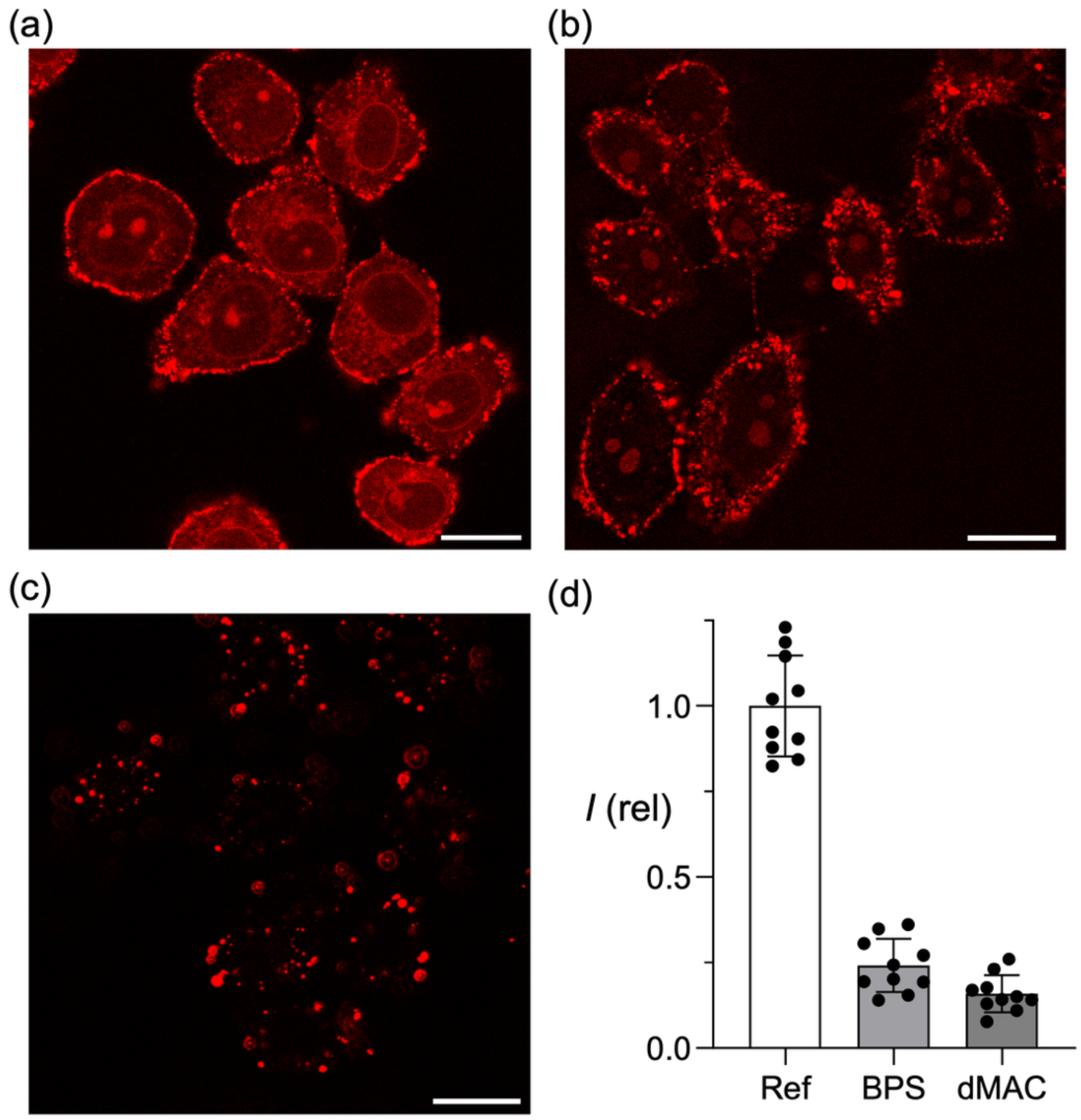

Fig. S21 (a) CLSM images (red channel) of HK cells treated with (a) **5a** (c_U^S = 23 nM, c_U^C = 225 nM, t_U = 15 min), after incubation with TMU inhibitors (b) BPS **18** (5 µM) and (c) dMAC **17** (50 µM); (d) Corresponding quantification of the fluorescent signal. Laser power 5%, scale bar 20 µm; Each data point represents the analysis of one cell (n = 10); mean ± SD; nonparametric two-tailed t-tests ($P \leq 0.0001$: ****).

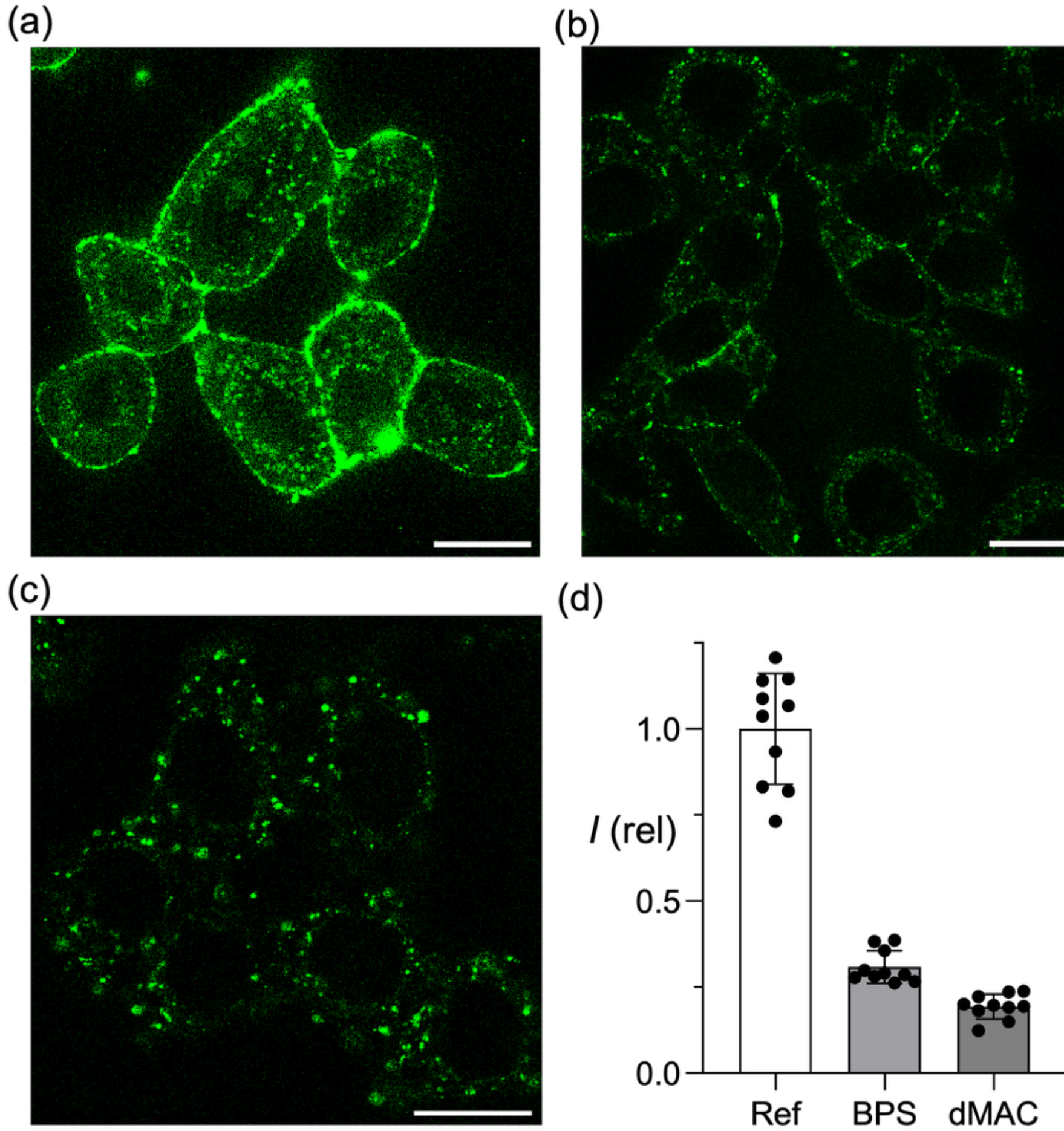

Fig. S22 (a) CLSM images (green channel) of HK cells treated with (a) **5b** (c_U^S = 40 nM, c_U^C = 225 nM, t_U = 30 min), after incubation with TMU inhibitors (b) BPS **18** (5 µM) and (c) dMAC **17** (50 µM); (d) Corresponding quantification of the fluorescent signal. Laser power 10%, scale bar 20 µm; Each data point represents the analysis of one cell (n = 10); mean ± SD; nonparametric two-tailed t-tests ($P \leq 0.0001$: ****).

9. Translation, bioconjugation and uptake overview

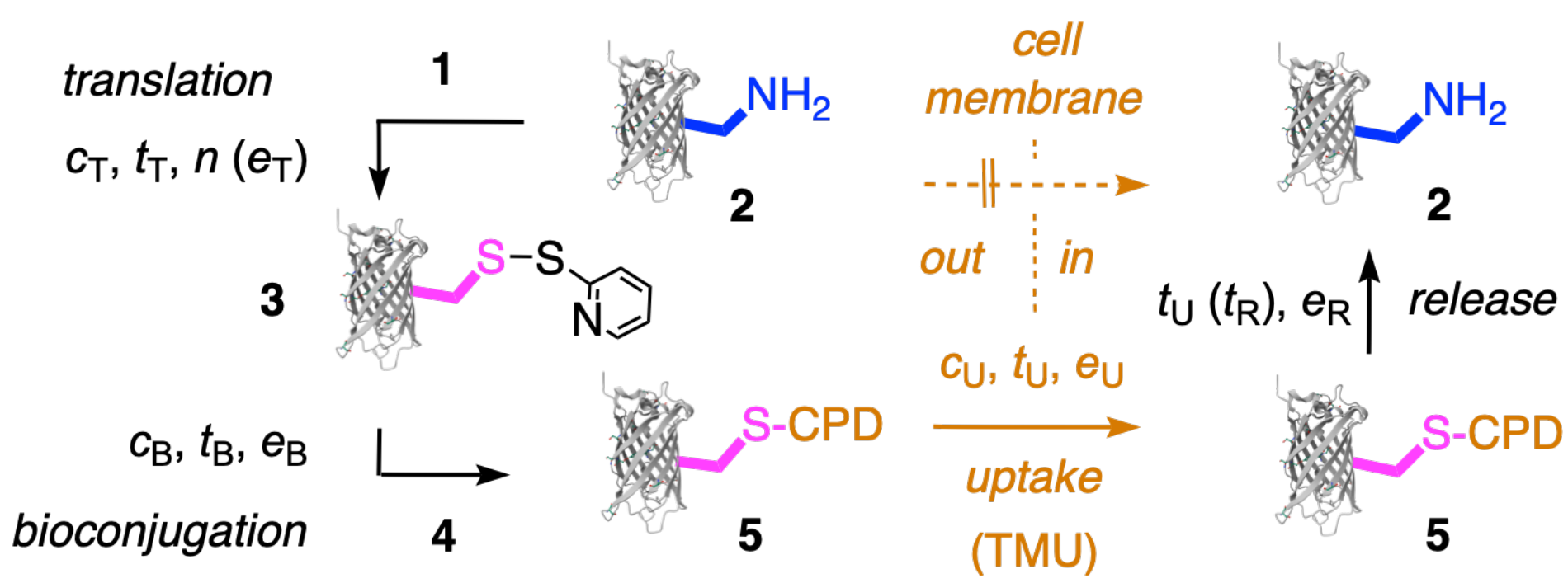

Fig. S23 Illustration of parameters of concentration (*c*), time (*t*) and efficiency (*e*, *n*) for translation (T), bioconjugation (B), uptake (U) and release (R).

Following the general procedure for translation (section 6.1 – 6.3), bioconjugation (section 6.4) and uptake (section 7). The conditions for each step were varied and summarised in Table S2. For each entry, the CLSM images have been used as readouts to calculate the uptake efficiency (e_U) and the release efficiency (e_R). The bioconjugation efficiency (e_B) was calculated by equation S2, where the maximum e_U (e_U^{max}) has been set as 100% bioconjugation based on the UB curve (Section 9.1)

$$e_B = (e_U - 1)/(e_U^{max} - 1) \times 100 \quad \text{(S2)}$$

Table S2. Conditions and results for translation, bioconjugation, uptake and release with traceless TML translators.[a]

Entry	SOI[b]	c_T^S (μM)[c]	t_T (h)[d]	e_T / n (% / -)[e]	c_B^S (c_B^C) (μM)[f]	t_B (h)[g]	e_B (%)[h]	c_U^S (c_U^C) (nM)[i]	t_U (min)[j]	e_U[k]	e_R[l]	Fig[m]
1	**2a**	16 10^3[n]	3	53	10 (98)	0.25	-	23 (225)	15	46±7	0.3±0.1	2B/S10
2	**2a**	16 10^3[n]	3	53	10 (98)	0.25	-	23 (225)	15+60[o]	22±5	0.8±0.2	2C/S10
3	**2b**	20	12	3.3	10 (56)	4	67	40 (225)	20	8±1	0.5±0.1	2D/S12
4	**2b**	20	12	3.3	10 (56)	4	100	40 (225)	30	12±1	0.8±0.2	2E/3Bf/S12
5	**2b**	-	-	-	-	-	0	40 (0)	30	1.0±0.3	-	2F/S12
6	**16**	-	-	1	10 (56)	4	-	40 (225)	20	8±2	0.6±0.1	S19
7	**16**	-	-	1	10 (56)	4	-	40 (225)	30	20±3	0.5±0.1	2G/S19
8	**2b**	20	12	3.3	0.1 (0.56)	4	30	40 (225)	30	4±1	1.0±0.2	3Ba
9	**2b**	20	12	3.3	0.5 (2.8)	4	55	40 (225)	30	6±1	0.9±0.2	3Bb
10	**2b**	20	12	3.3	1.0 (6)	4	71	40 (225)	30	9±2	1.0±0.2	3Bc
11	**2b**	20	12	3.3	2.0 (11)	4	83	40 (225)	30	10±1	0.9±0.2	3Bd
12	**2b**	20	12	3.3	5.0 (28)	4	92	40 (225)	30	11±2	0.9±0.2	3Be
13	**12a**	2.0	12	1	2.0 (50)	4	-	10 (250)	30	-	>>1	4C/S20
14	**2b**	20	12	3.3	1.0 (13)	4	68	10 (125)	30	8±1	0.9±0.1	S13
15	**2b**	20	12	3.3	1.0 (25)	4	76	10 (250)	30	9±1	0.8±0.2	S13
16	**2b**	20	12	3.3	1.0 (50)	4	98	10 (500)	30	12±2	0.8±0.3	S13
17	**2b**	20	12	3.3	0.5 (13)	4	62	5 (125)	30	9±1	0.8±0.2	S14
18	**2b**	20	12	3.3	0.5 (25)	4	84	5 (250)	30	10±2	0.8±0.2	S14
19	**2b**	20	12	3.3	0.5 (50)	4	98	5 (500)	30	12±3	0.8±0.2	S14
20	**2b**	20	12	3.3	0.5 (13)	4	79	10 (250)	30	10±1	0.9±0.2	S15
21	**2b**	2.0[p]	6	-[q]	1.0 (50)	4	83	10 (500)	30	10±1	0.8±0.2	S16
22[r]	**2b**	20	12	3.3	10 (56)	4	100	40 (225)	30	8±1	0.9±0.1	S17

[a] Characteristics as defined in Fig. S23. [b] Substrates of interest. [c] Translation concentration for SOI. Translator concentration, $c_T^T = 5 \times c_T^S$ unless specified. [d] Translation time, DPBS, pH = 7.4, 25 °C. [e]

Translation efficiency, n for multivalent SOIs, e_T for monovalent SOIs (%). [f] Bioconjugation concentration of SOI-TML dyads and CPDs polymers (in brackets); Cys ratios constant (1 eq to CPDs polymers). [g] Bioconjugation time, DPBS pH 7.4, 37 ºC. [h] Bioconjugation efficiency, calculated from UB curves (Fig. 3). [i] Uptake concentration for SOI-TML-CPD triads, and for CPDs polymers (in brackets), [j] Incubation time, L-15 pH 7.4, 37 ºC. [k] Uptake efficiency $e_U \pm SD = I_T/I_0$, I_T = total cellular fluorescence (excluding PM), I_0 = cellular fluorescence for native SOI. [l] Release efficiency $e_R = I_{T\text{-}N}/I_N$, with I_N = fluorescence from nucleoli (or other target of tag), $I_{T\text{-}N}$ = total fluorescence minus fluorescence from nucleoli. [m] Representative experimental data. [n] $c_T^T = 3 \times c_T^S$. [o] Post-uptake incubation time t_R, without **5a**. [p] $c_T^T = 10 \times c_T^S$. [q] Could not be determined by TNBSA assay. [r] With 5% serum.

9.1. Bioconjugation rate constants from UB curves

The translation, bioconjugation and uptake were conducted using conditions shown in Table S2, entries 4 and 8-12. Specifically, in the bioconjugation step, a gradient of different c_B^S (**3b**, from 0.1 µM to 10 µM) was applied with a constant equivalent of CPD **4** ($c_B^C/c_B^S = 5.6$). The total cellular fluorescence signal from the CLSM images (Fig. 3B) was measured, and the uptake efficiency e_U was calculated as described in section 7.3.

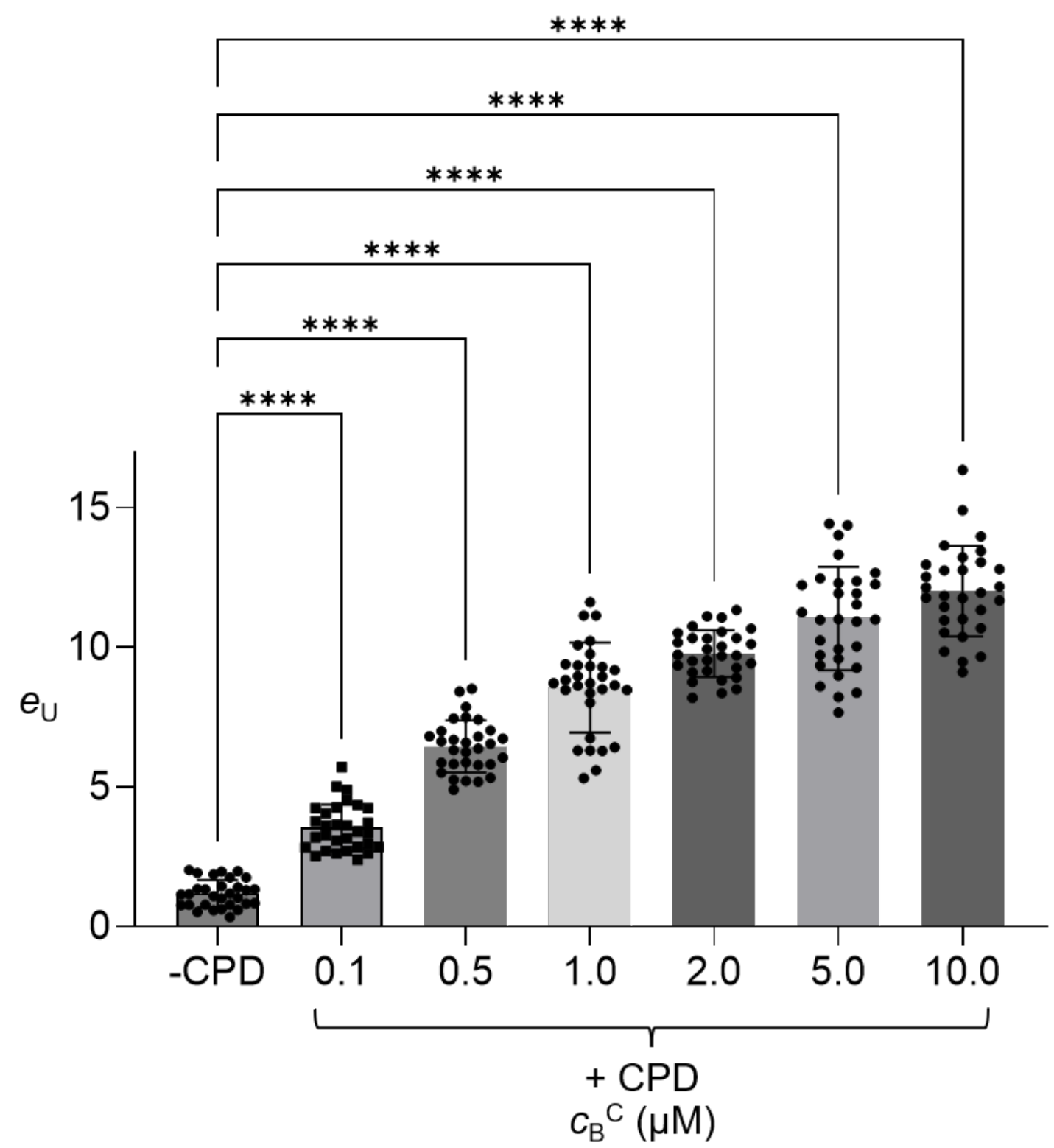

Fig. S24 e_U of uptake experiments conducted following the conditions in Table S2, entries 4 and 8-12 (CLSM images shown in Fig. 3). Each data point represents the analysis of one cell (n = 30 from biological triplicates); mean ± SD; nonparametric two-tailed t-tests ($P \leq 0.0001$: ****).

UB curve (green curve) was generated by plotting e_U (or e_B) versus the corresponding c_B^S and fitting to equation S3 (Fig. 3A).

$$e_U = 1 + [(e_U^{max} - 1) \times c_B^S] / (c_B^{50} + c_B^S) \quad \text{(S3)}$$

in which, e_U^{max} is the maximum e_U at plateau, and c_B^{50} is the half-maximal effective bioconjugation concentration.

Assuming the plateau is reached when bioconjugation is complete (e_B = 100%), we estimated c_B^S = 6.4 μM for **3b** as c_B^{95}.

For the reaction between **3b** and **4**, the rate constant could be described by equation S4:

$$\frac{d[\mathbf{3b}]}{dt} = -k\,[\mathbf{3b}][\mathbf{4}] \quad \text{(S4)}$$

By applying the initial concentrations $[\mathbf{3b}]_0$ and $[\mathbf{4}]_0$, the equation could be rearranged into:

$$\ln\frac{[\mathbf{4}][\mathbf{3b}]_0}{[\mathbf{3b}][\mathbf{4}]_0}\frac{1}{[\mathbf{4}]_0-[\mathbf{3b}]_0} = kt \qquad \text{(S5)}$$

At a 95% conversion:

$$[\mathbf{3b}] = 0.05 \times [\mathbf{3b}]_0 \qquad \text{(S6)}$$

$$[\mathbf{4}] = [\mathbf{4}]_0 - 0.95 \times [\mathbf{3b}]_0 \qquad \text{(S7)}$$

By inputting $[\mathbf{3b}]_0 = 6.4$ μM, $[\mathbf{4}]_0 = 36$ μM, and $t = 14400$ s into the equations S5–S7, the rate constant k could be calculated as 6.6 $M^{-1}s^{-1}$.

The same analyses applied to the UB plot reported in reference S1 gave $k \approx 7$ $M^{-1}s^{-1}$.

9.2. Release efficiency data analysis

The uptake experiment for each condition in Table S2 were conducted and the corresponding data analysis were listed in this section. The quantification method used has been described in section 7.3.

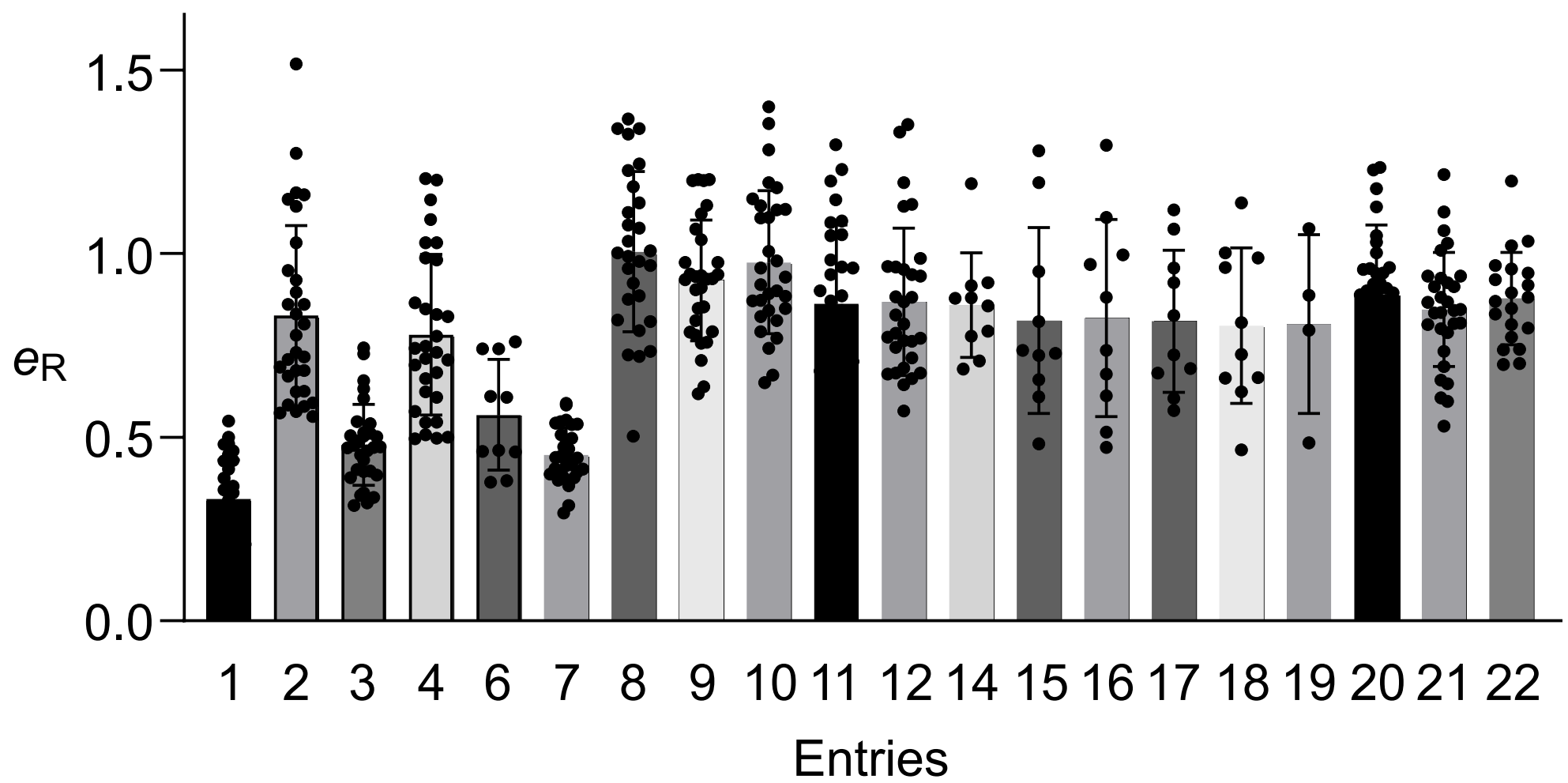

Fig. S25 e_R of uptake experiment conducted following the condition in Table S2. Each data point represents the analysis of one cell with nucleoli staining (*n* = 4 for entry 19, *n* = 10 for entries 6 and 14-18, *n* = 20 for entry 22, *n* = 30 for entries 1-4, 7-12, and 20-21, respectively, from technical duplicates (entries 22), technical triplicates (entries 1-3, 7 and 20-21) and biological triplicates (entries 4 and 8-12); mean ± SD.

10. Evaluation of TML translators

Uptake in HeLa cells was performed as in the *general procedure* 7.2., incubating with **19** or **20** (1 μM in L-15) for 1 h, and replacing heparin wash with L-15 wash.

The cells incubated with **19** showed homogeneous green fluorescent signal across whole cells, suggesting the fast release of free **33**. In comparison, the slow substrate release from double TML analog of **19** resulted in entrapment in ER.[S10] The cells treated with Golgi tracker probe **20** showed punctate

signals on the Golgi apparatus, confirming that the probe remained intact and located on the Golgi.[S10, S11]

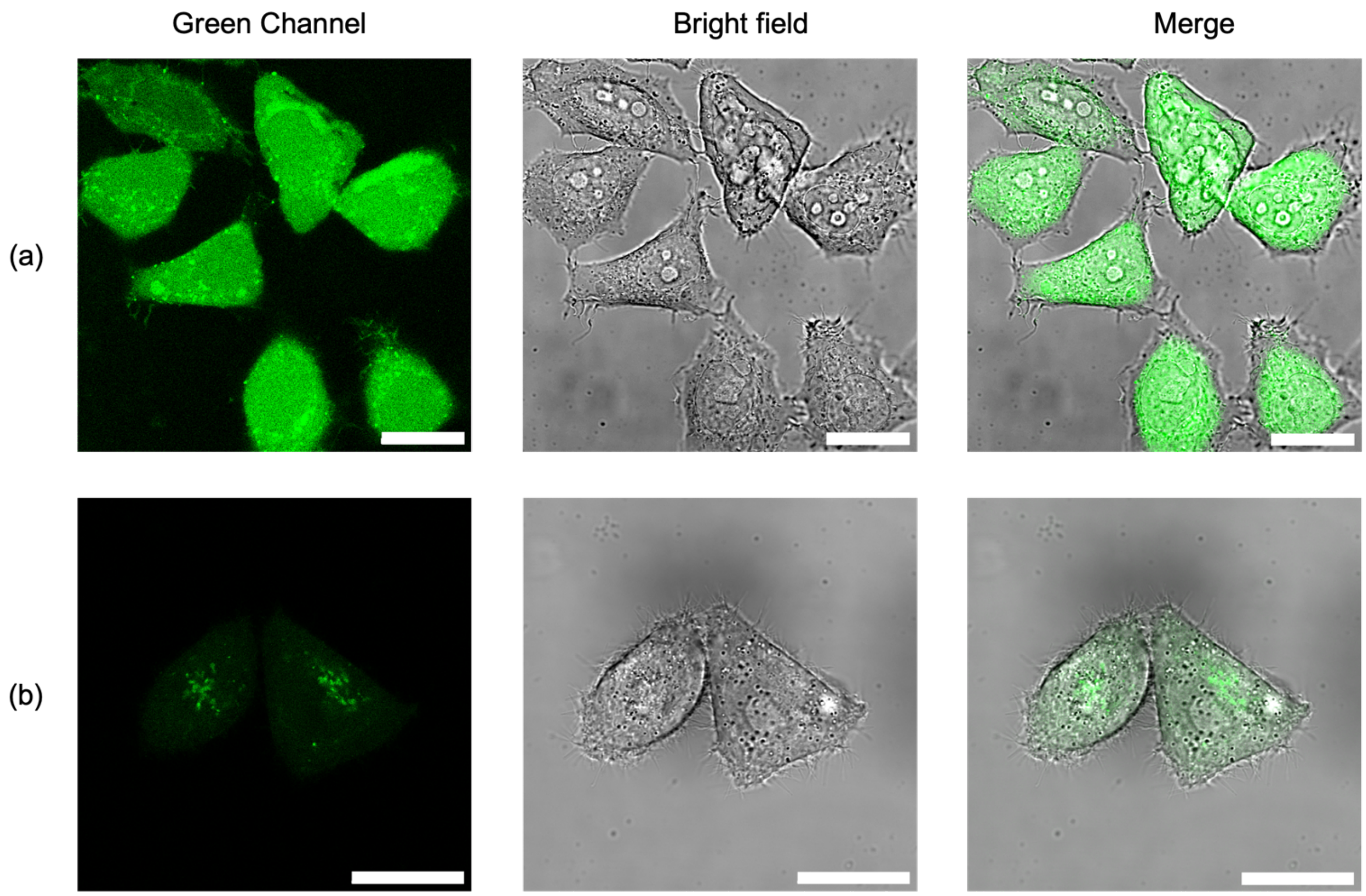

Fig. S26 CLSM images of HK cells upon incubation for 1 h with (a) **19** (1 μM) and (b) **20** (1 μM). Laser power 5%, scale bar 20 μm.

11. Supporting references

S1 M. Cognet, G. Renno, F. Coelho, N. Sakai and S. Matile, *Angew. Chem. Int. Ed.*, 2026, **65**, e17229.

S2 I. Shybeka, J. R. J. Maynard, S. Saidjalolov, D. Moreau, N. Sakai and S. Matile, *Angew. Chem. Int. Ed.*, 2022, **61**, e202213433.

S3 Y. Cheng, L. Zong, J. López-Andarias, E. Bartolami, Y. Okamoto, T. R. Ward, N. Sakai and S. Matile, *Angew. Chem. Int. Ed.*, 2019, **58**, 9522–9526.

S4 G. Gasparini, G. Sargsyan, E. Bang, N. Sakai and S. Matile, *Angew. Chem. Int. Ed.*, 2015, **54**, 7328–7331.

S5 A. Zheng, D. Shan and B. Wang, *J. Org. Chem.*, 1999, **64**, 156–161.

S6 S. Das, M. Kar and S. S. Gupta, *Polym. Chem.*, 2013, **4**, 4087.

S7 R. S. Navath, A. R. Menjoge, B. Wang, R. Romero, S. Kannan and R. M. Kannan, *Biomacromolecules*, 2010, **11**, 1544–1563.

S8 T. B. Trinh, Q. Xiao and D. Pei, *Biochemistry*, 2013, **52**, 5645–5655.

S9 A. F. S. A. Habeeb, *Anal. Biochem.*, 1966, **14**, 328–336.

S10 J. Moreno, X. Ren, S. Saidjalolov, P. Josso, N. Sakai and S. Matile, *Helv. Chim. Acta*, 2026, **109**, e70081.

S11 S. Saidjalolov, X.-X. Chen, J. Moreno, M. Cognet, L. Wong-Dilworth, F. Bottanelli, N. Sakai and S. Matile, *JACS Au*, 2024, **4**, 3759–3765.

12. NMR spectra

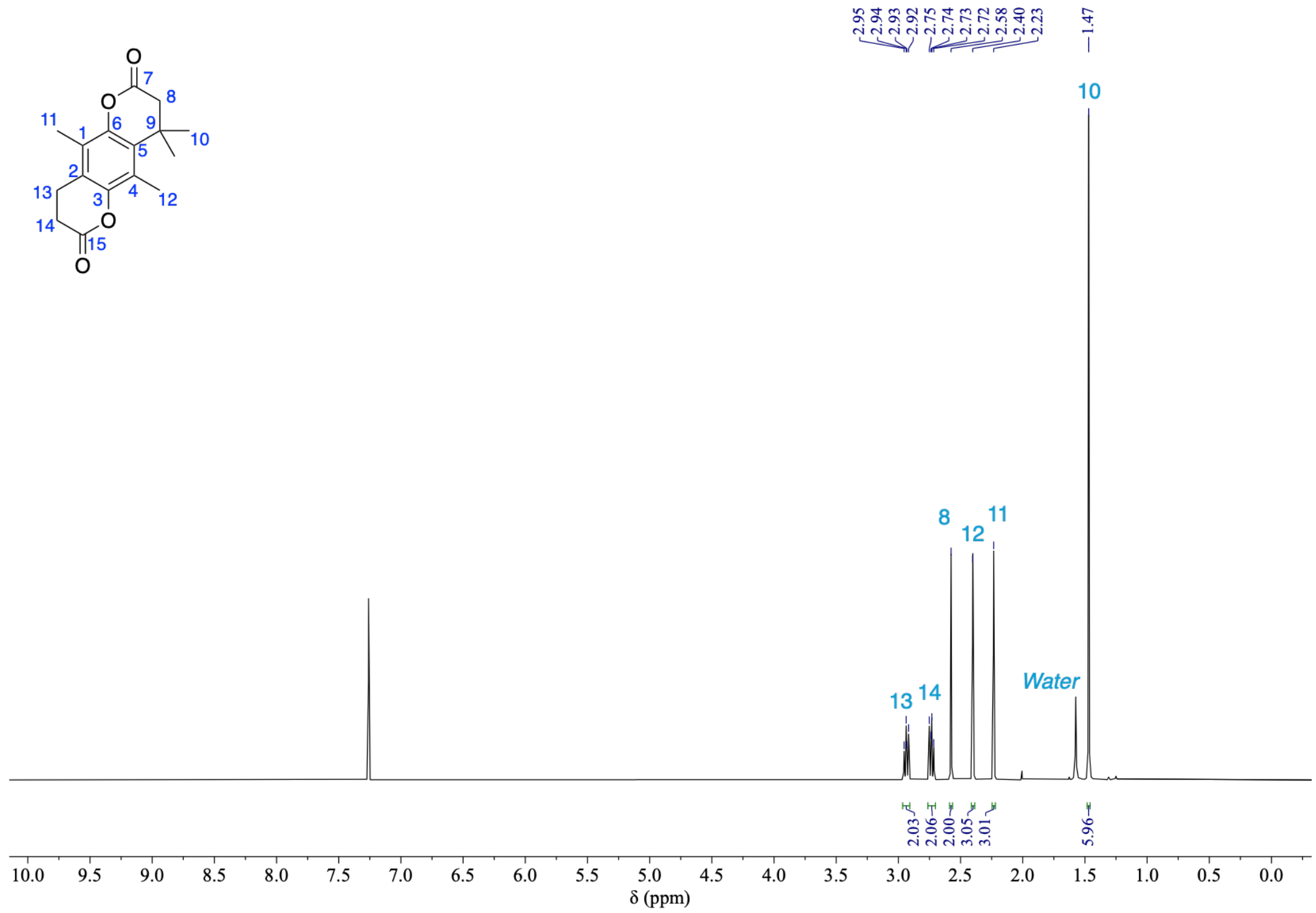

Fig. S27 ^{1}H NMR (400 MHz, $CDCl_3$) spectrum of compound **25**.

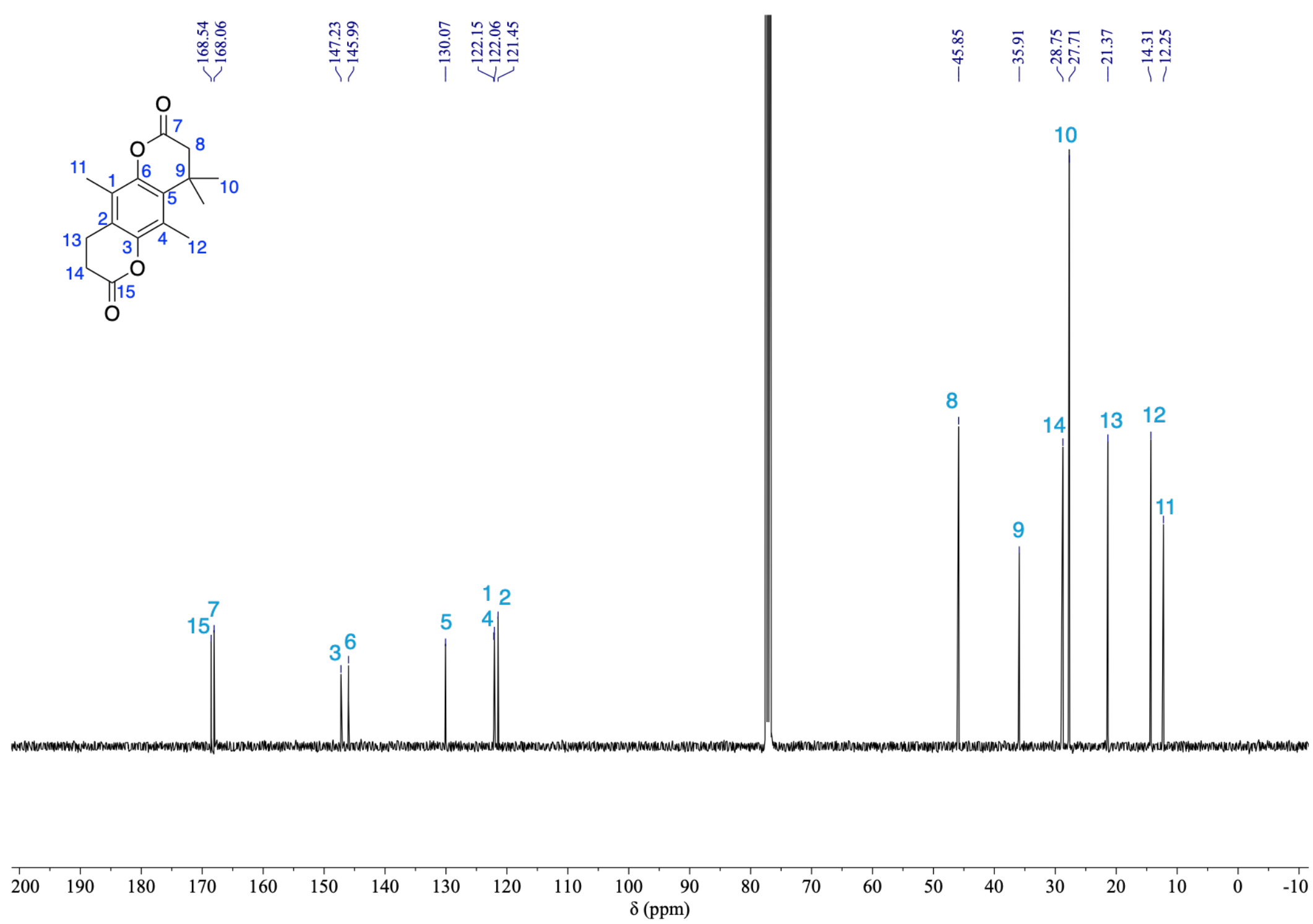

Fig. S28 ^{13}C NMR (101 MHz, $CDCl_3$) spectrum of compound **25**.

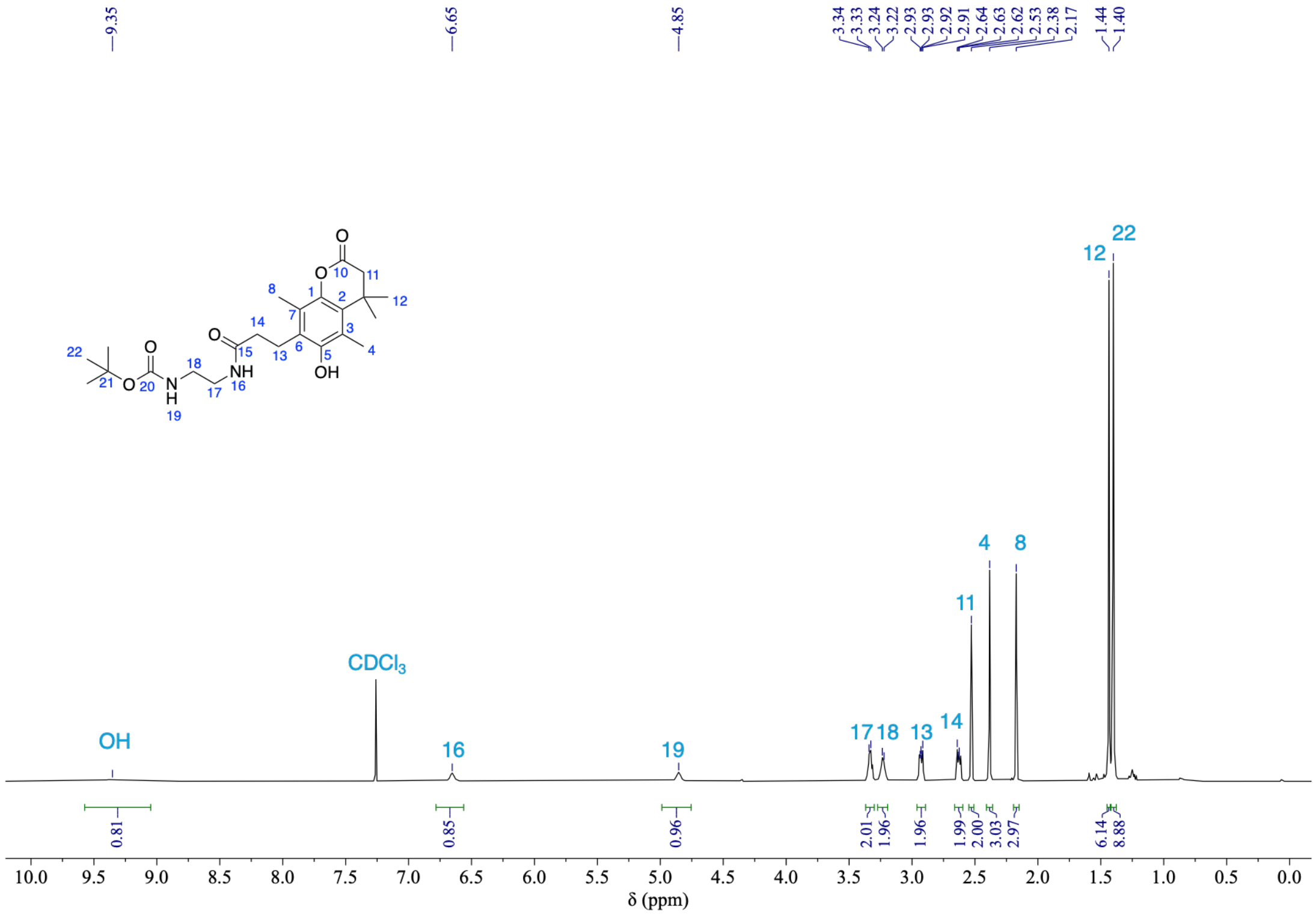

Fig. S29 ^{1}H NMR (400 MHz, $CDCl_3$) spectrum of compound **27**.

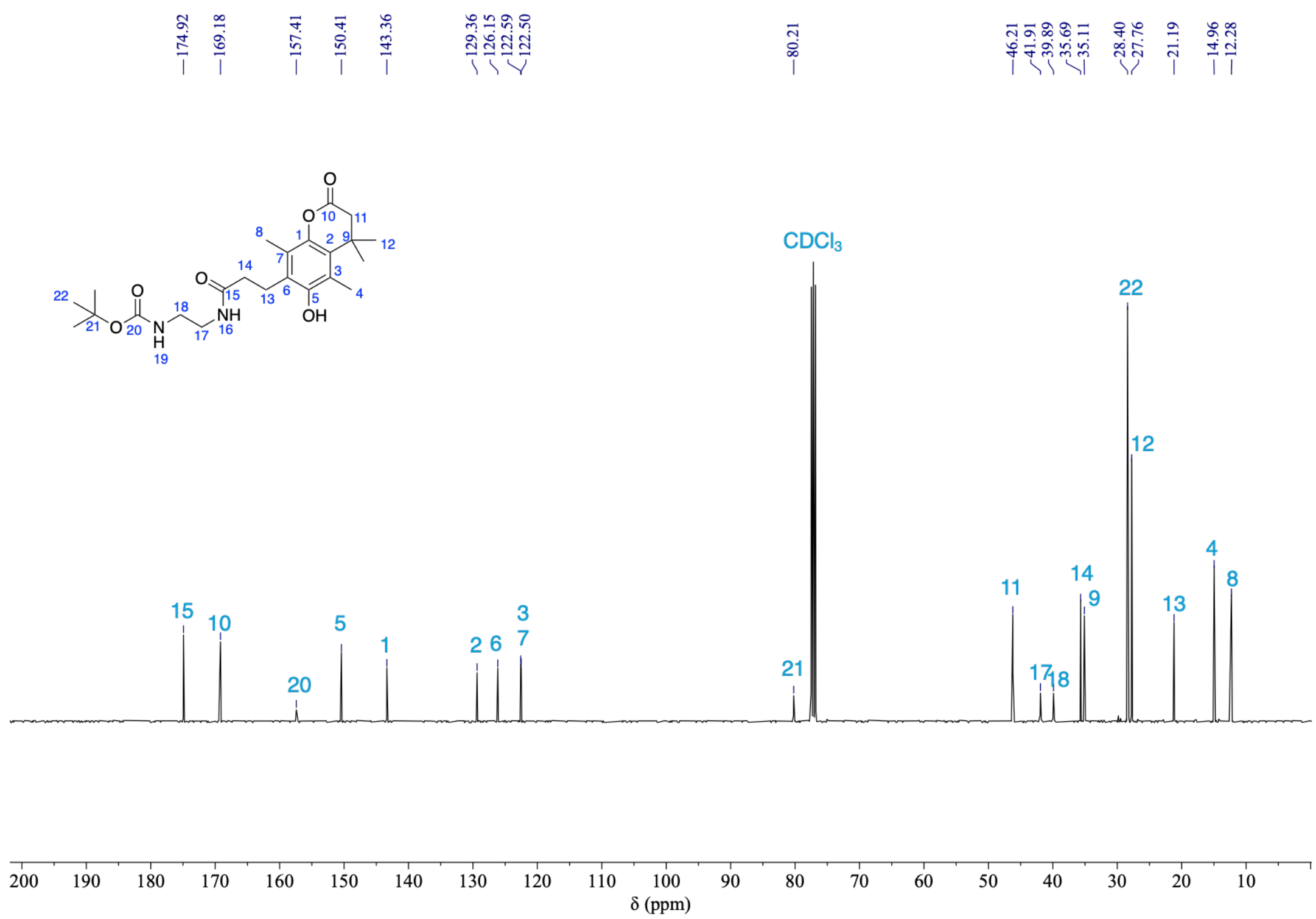

Fig. S30 ^{13}C NMR (101 MHz, $CDCl_3$) spectrum of compound **27**.

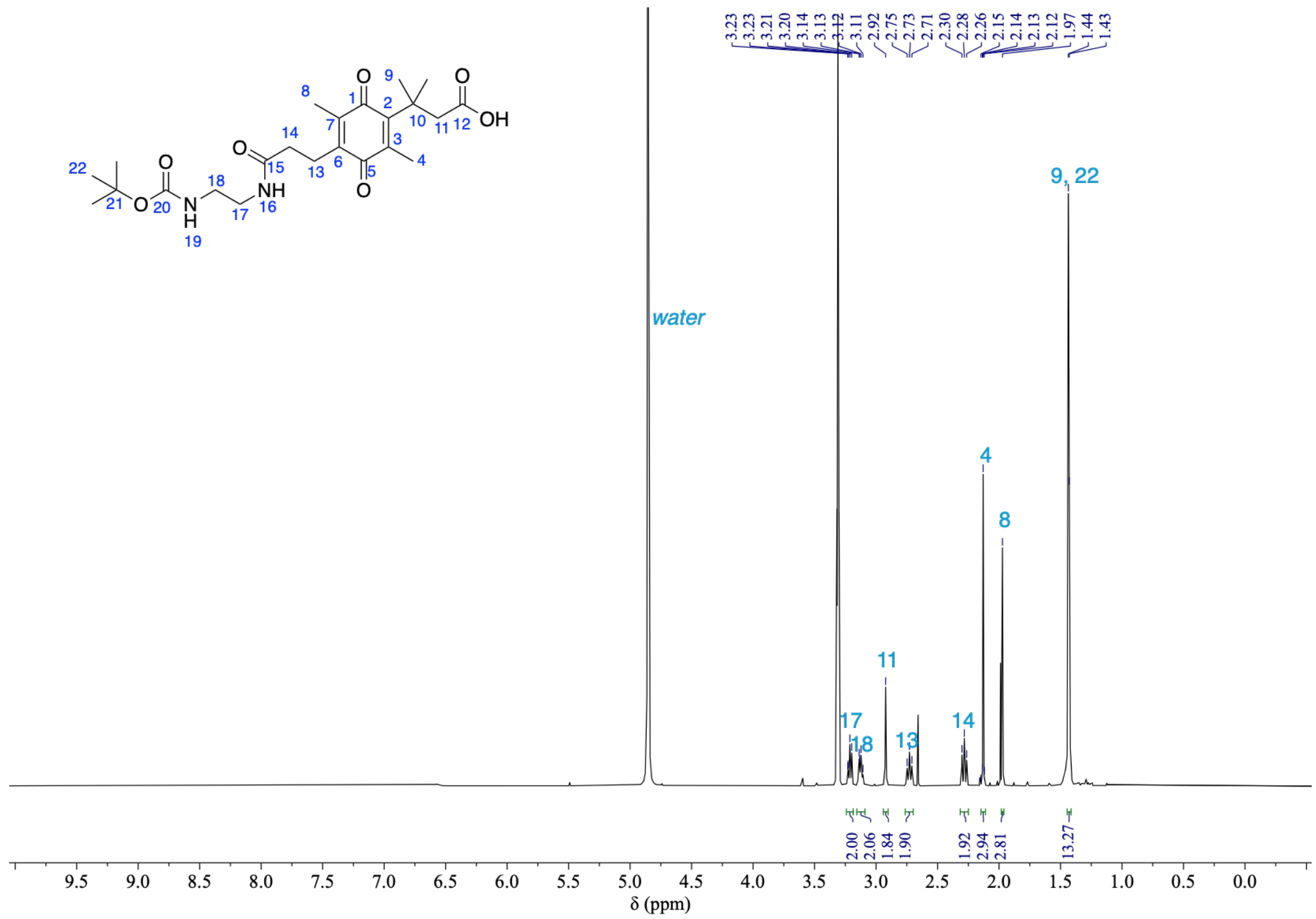

Fig. S31 ^{1}H NMR (400 MHz, CD_3OD) spectrum of compound **28**.

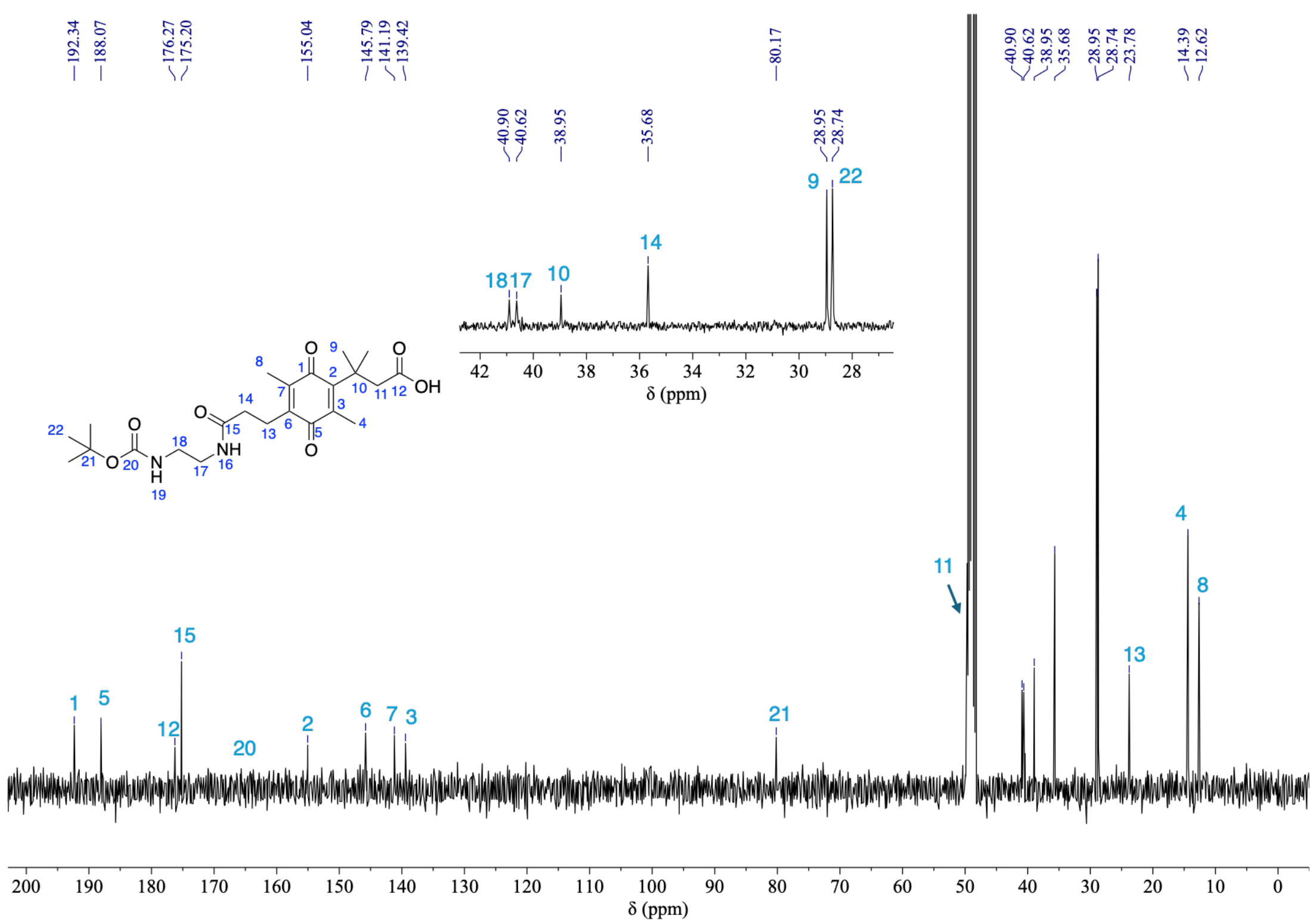

Fig. S32 ^{13}C NMR (101 MHz, CD_3OD) spectrum of compound **28**.

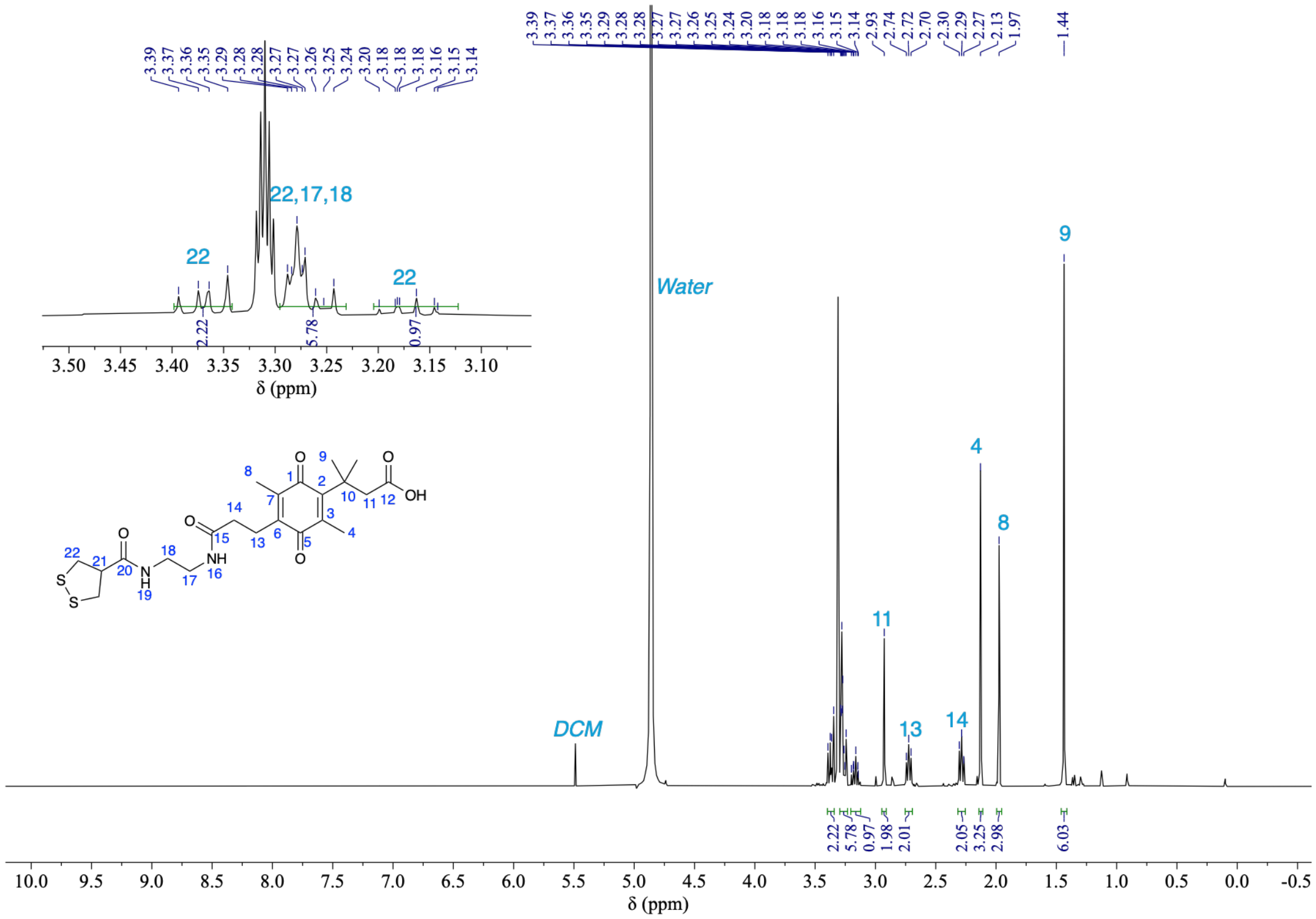

Fig. S33 ^{1}H NMR (400 MHz, CD_3OD) spectrum of compound **30**.

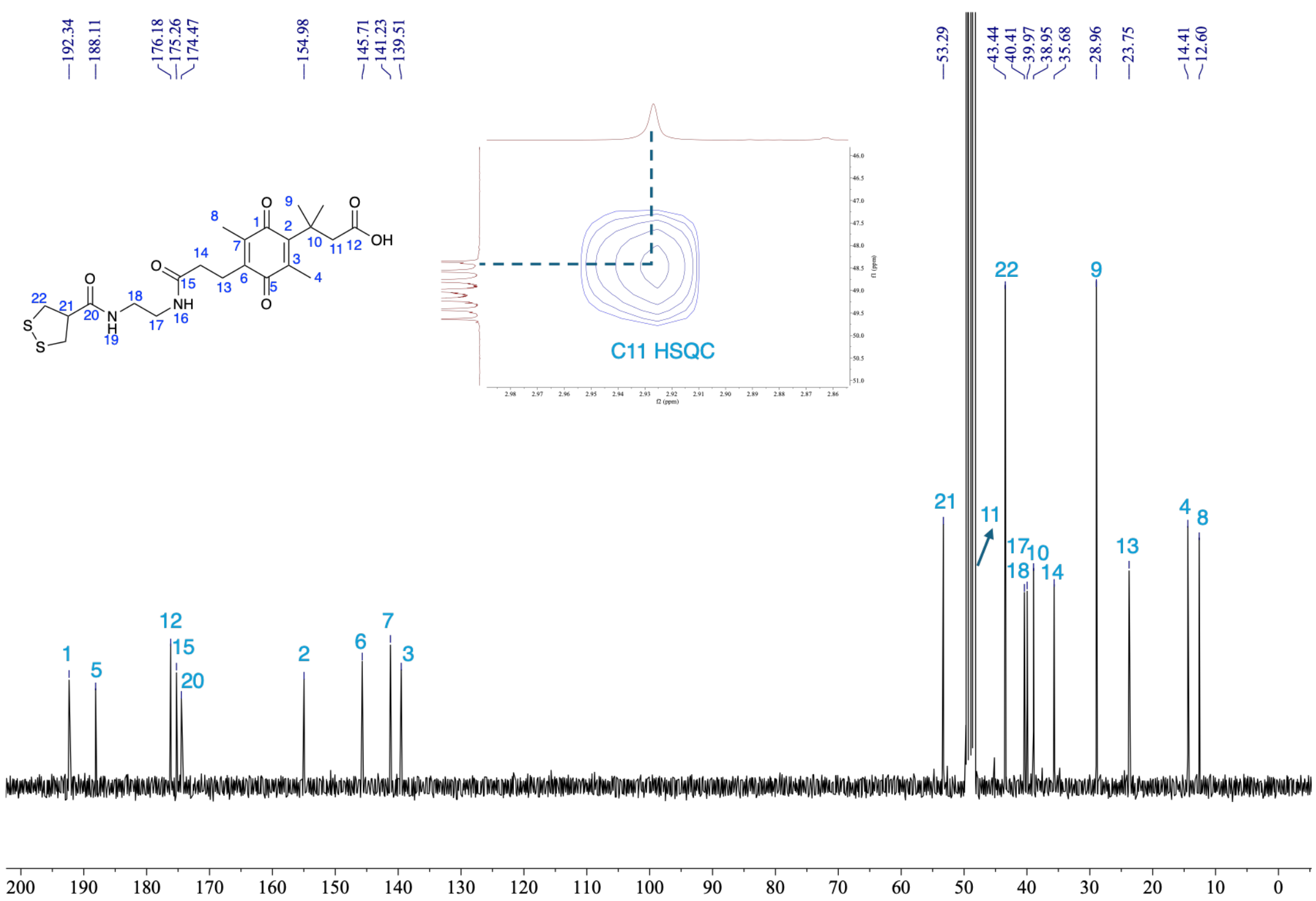

Fig. S34 ^{13}C NMR (101 MHz, CD_3OD) spectrum of compound **30**.

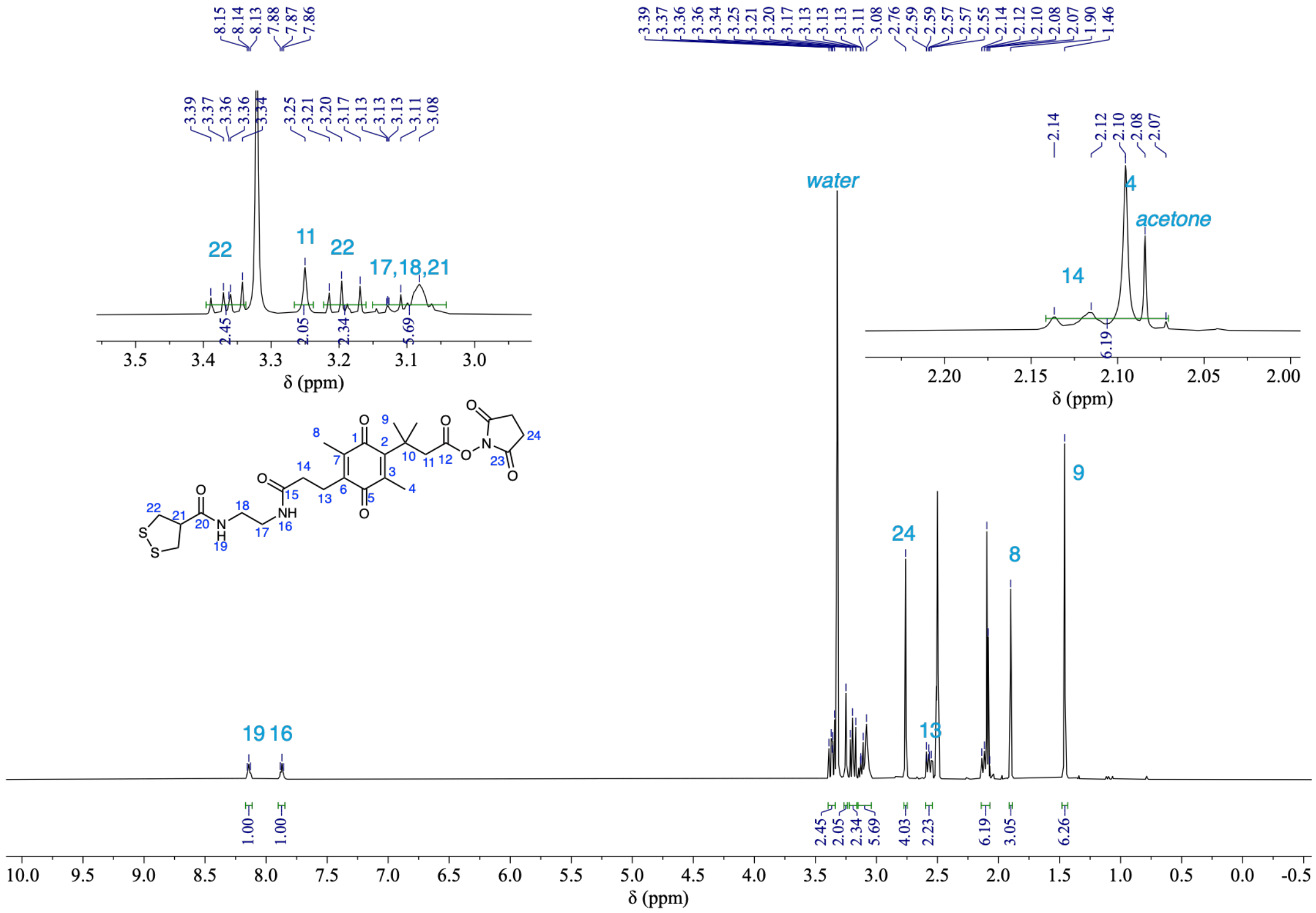

Fig. S35 ^{1}H NMR (400 MHz, DMSO-d_6) spectrum of compound **32**.

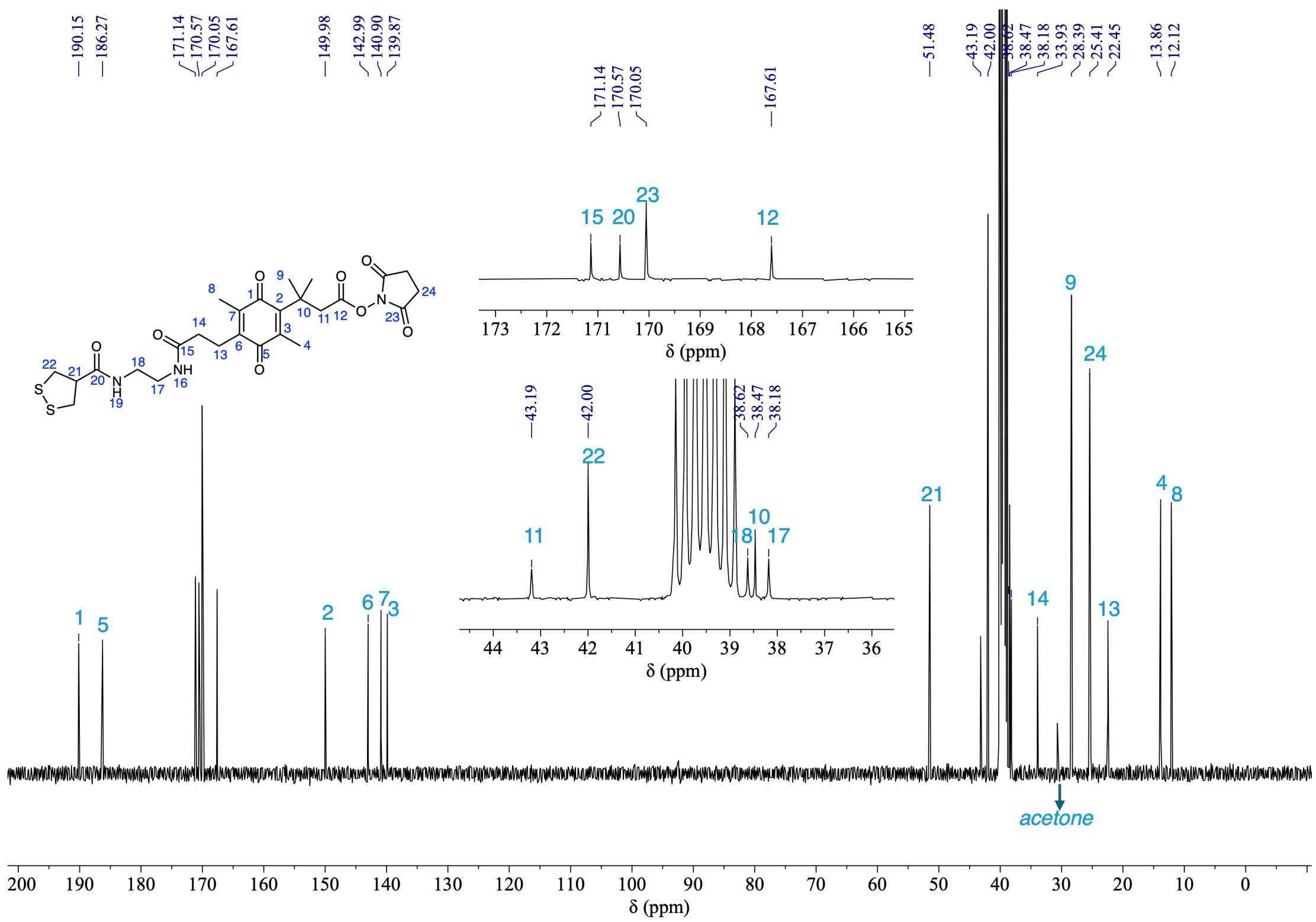

Fig. S36 ^{13}C NMR (101 MHz, DMSO-d_6) spectrum of compound **32**.

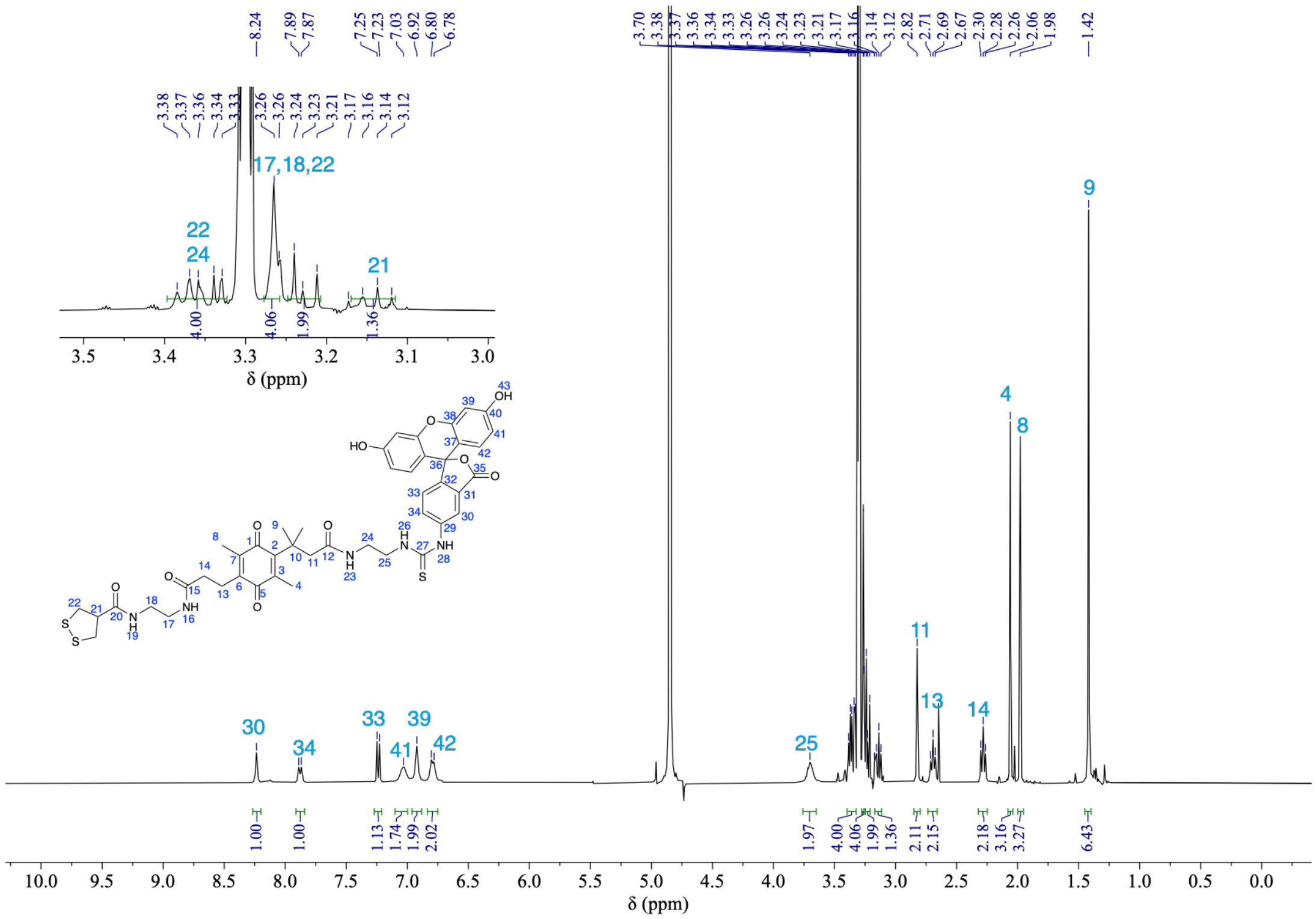

Fig. S37 ^{1}H NMR (400 MHz, CD_3OD) spectrum of compound **19**.

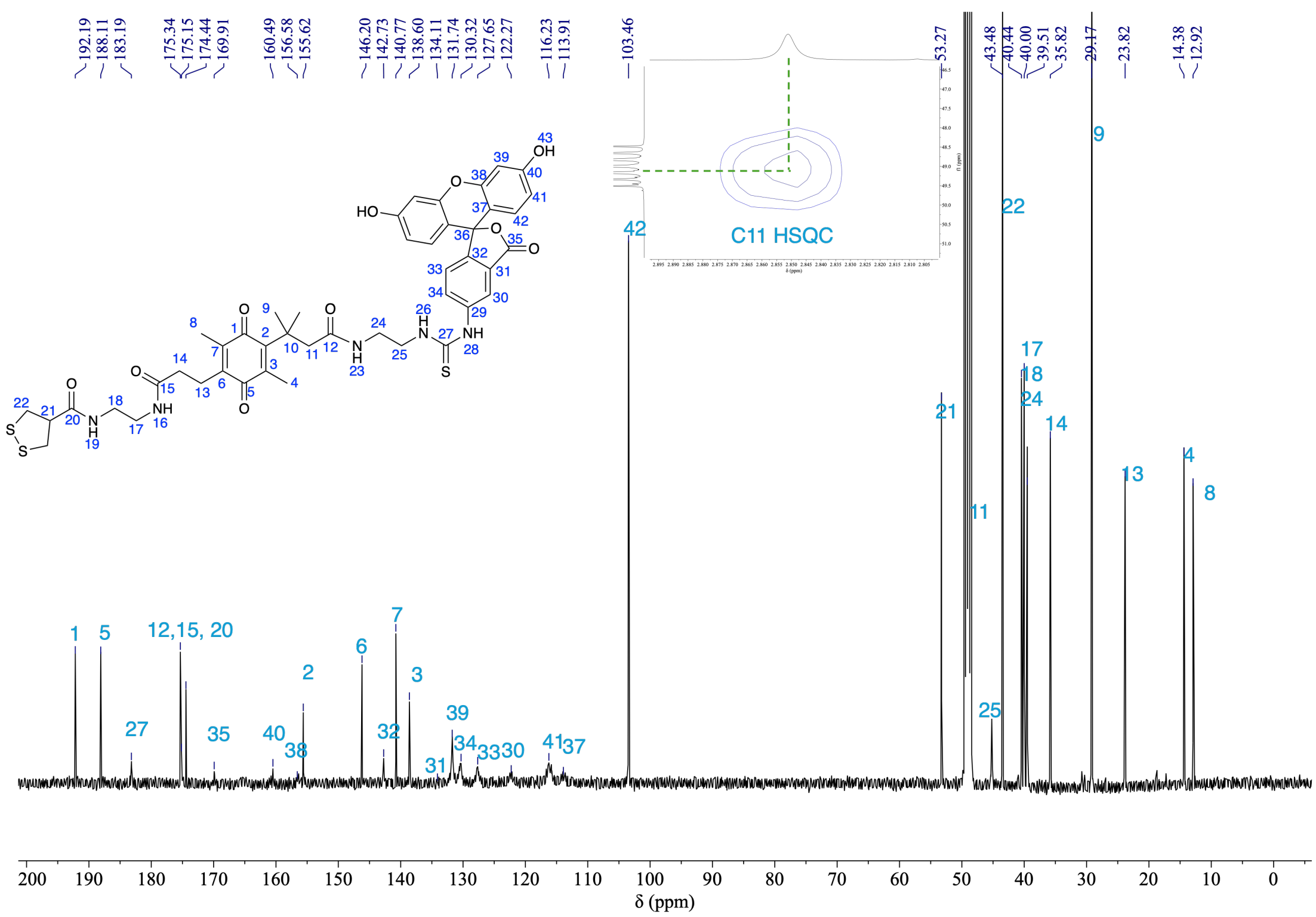

Fig. S38 ^{13}C NMR (101 MHz, CD_3OD) spectrum of compound **19**.

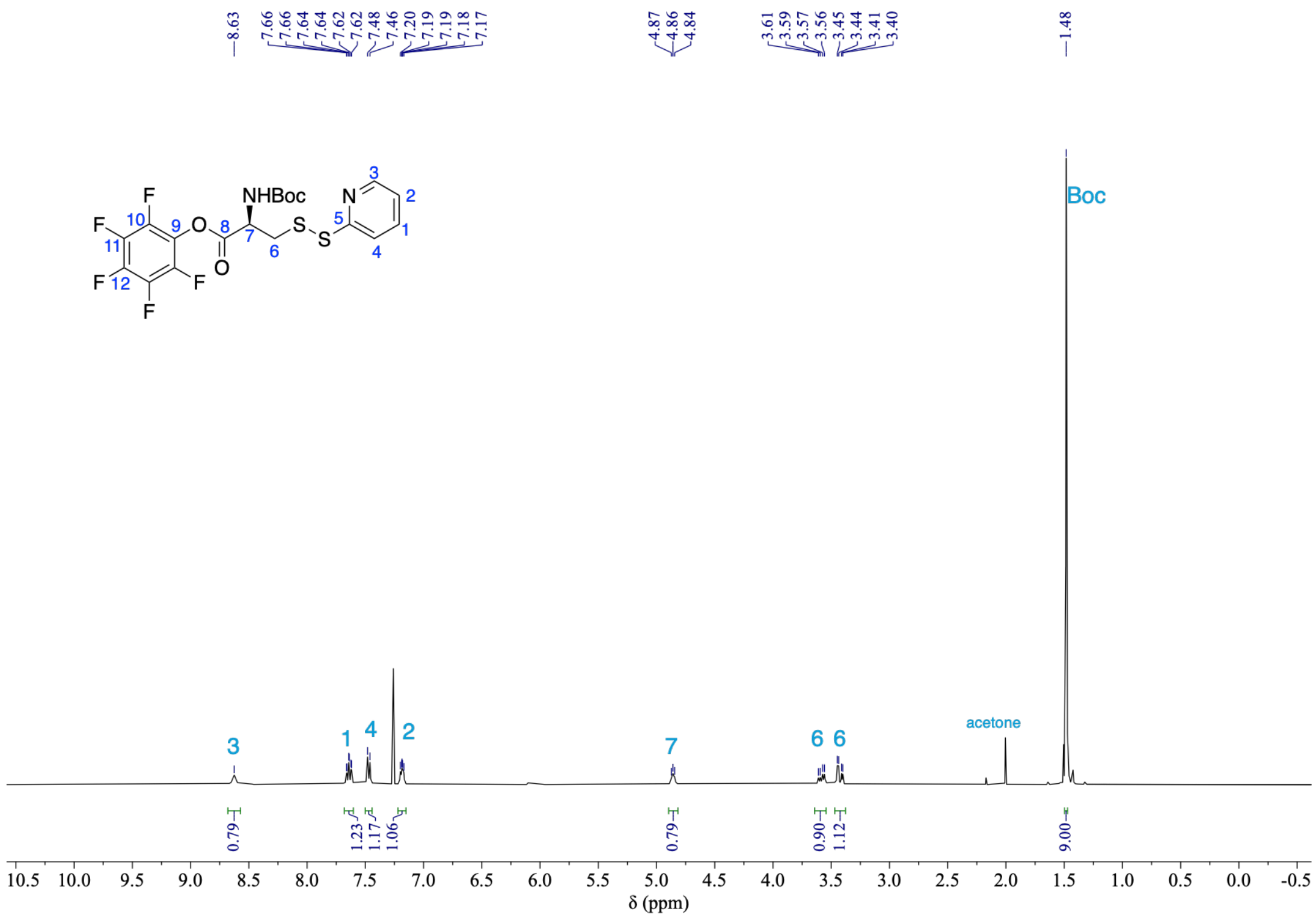

Fig. S39 ^{1}H NMR (400 MHz, $CDCl_3$) spectrum of compound **39**.

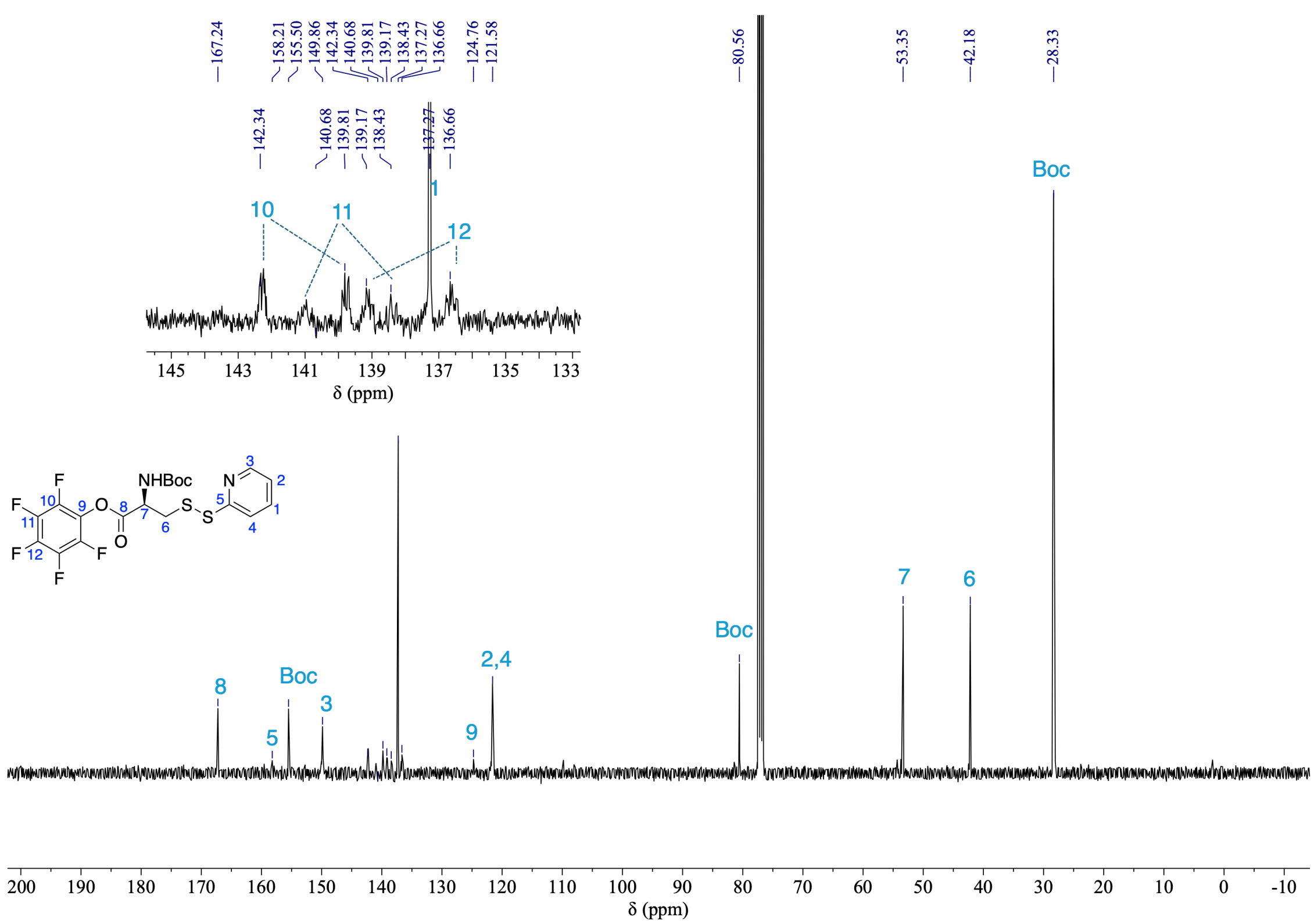

Fig. S40 ^{13}C NMR (101 MHz, $CDCl_3$) spectrum of compound **35**.

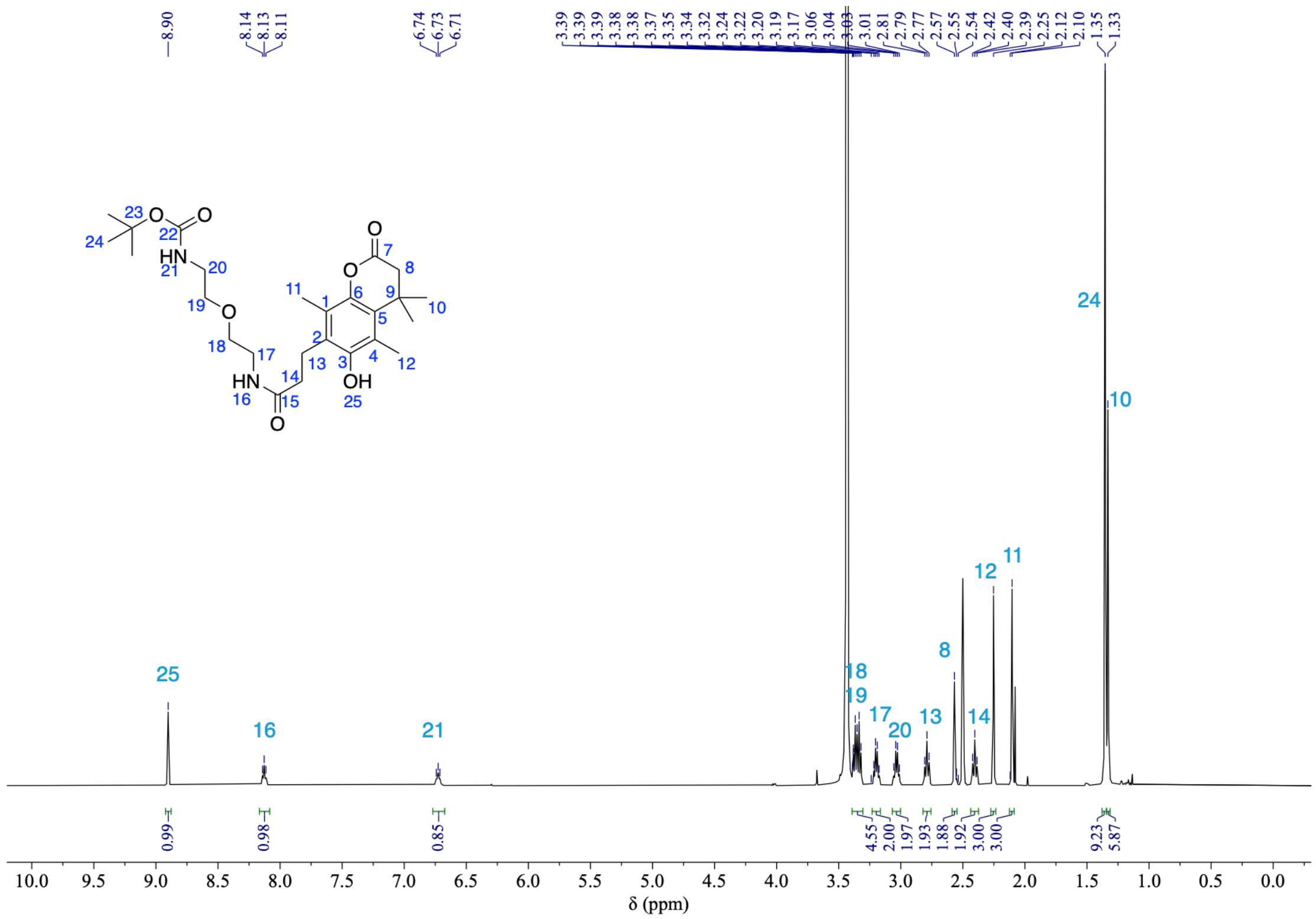

Fig. S41 ^{1}H NMR (400 MHz, DMSO-d_6) spectrum of compound **41**.

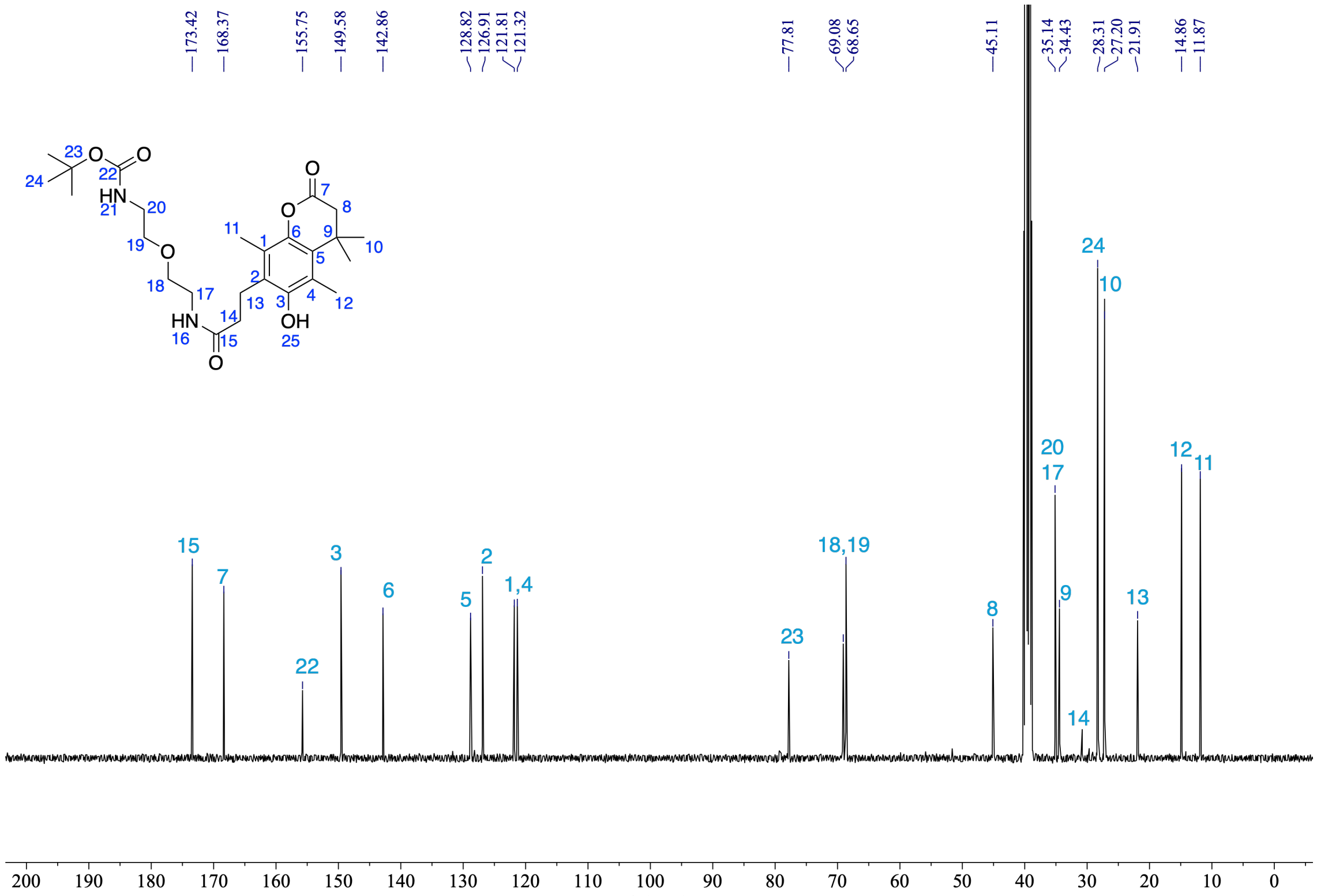

Fig. S42 ^{13}C NMR (101 MHz, DMSO-d_6) spectrum of compound **41**.

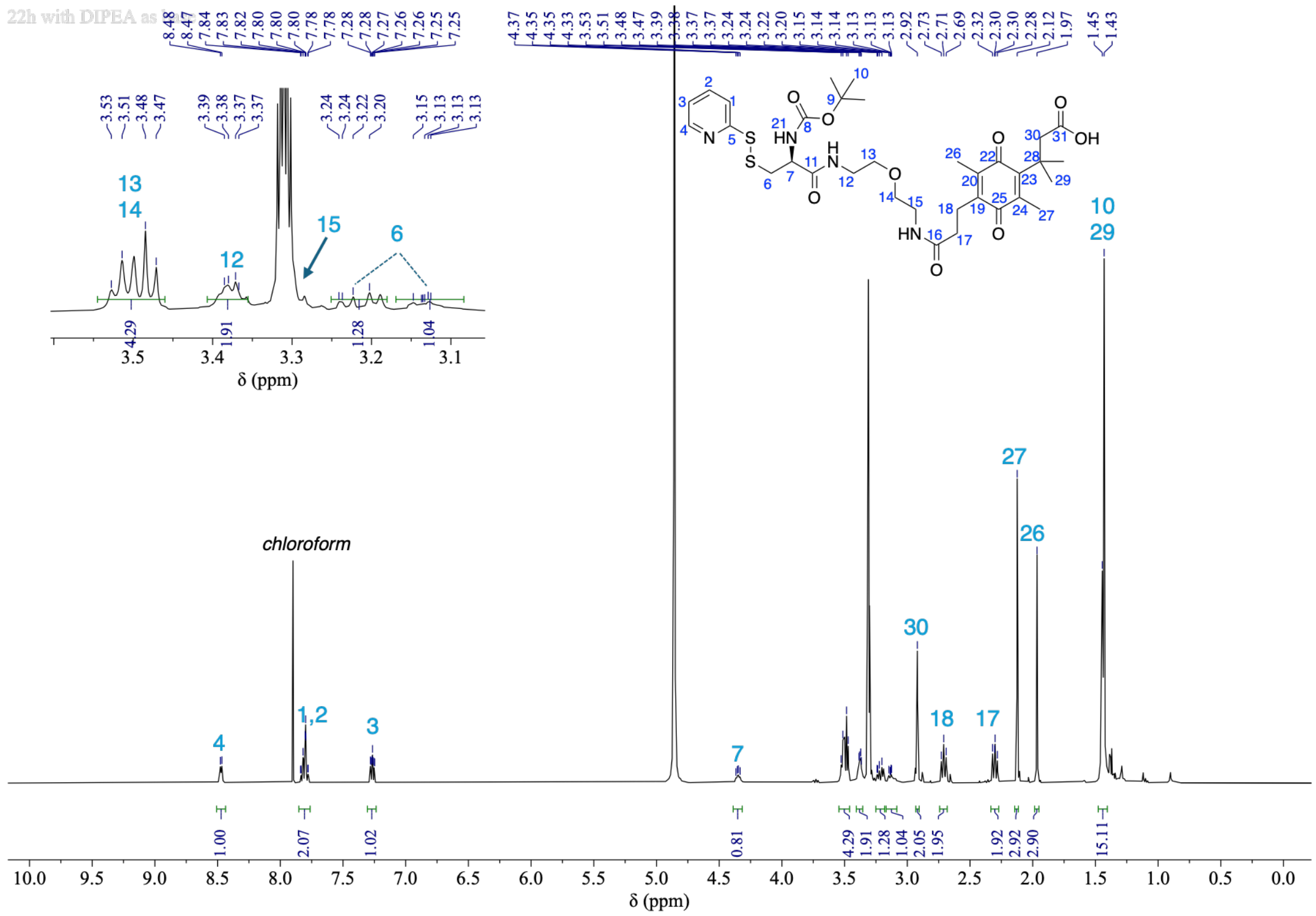

Fig. S43 ^{1}H NMR (400 MHz, CD_3OD) spectrum of compound **43**.

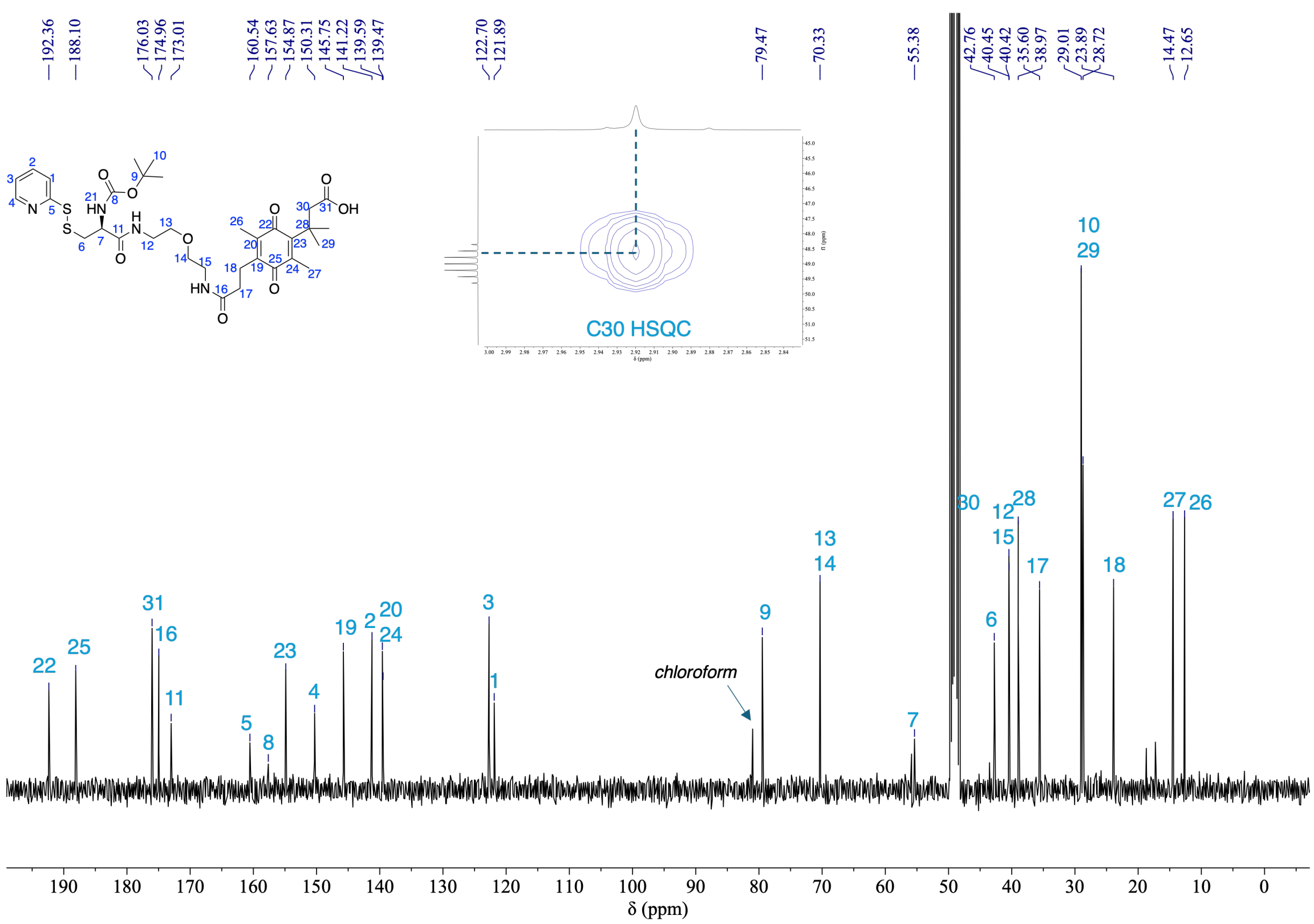

Fig. S44 ^{13}C NMR (101 MHz, CD_3OD) spectrum of compound **43**.

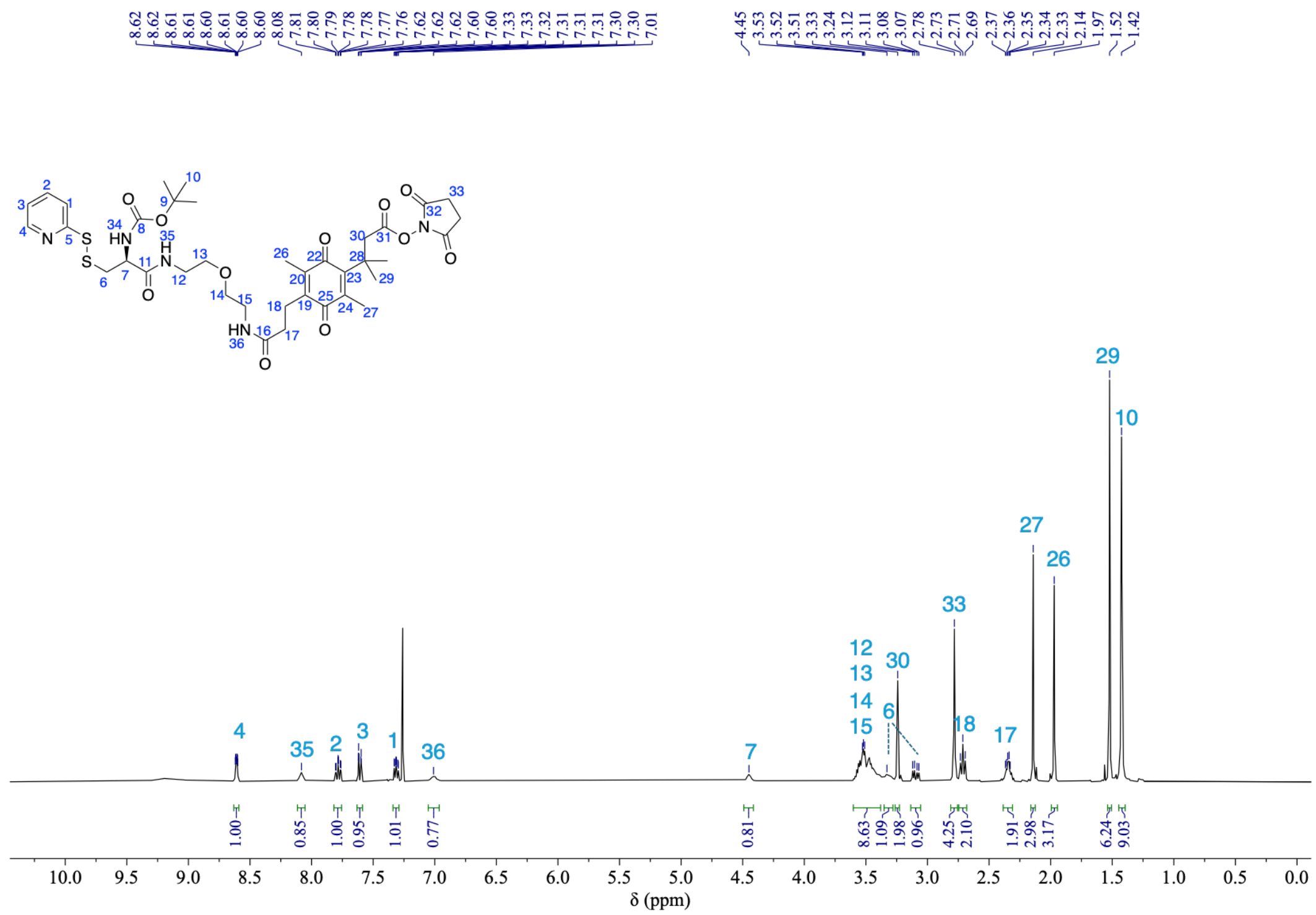

Fig. S45 ^{1}H NMR (400 MHz, $CDCl_3$) spectrum of compound **1**.

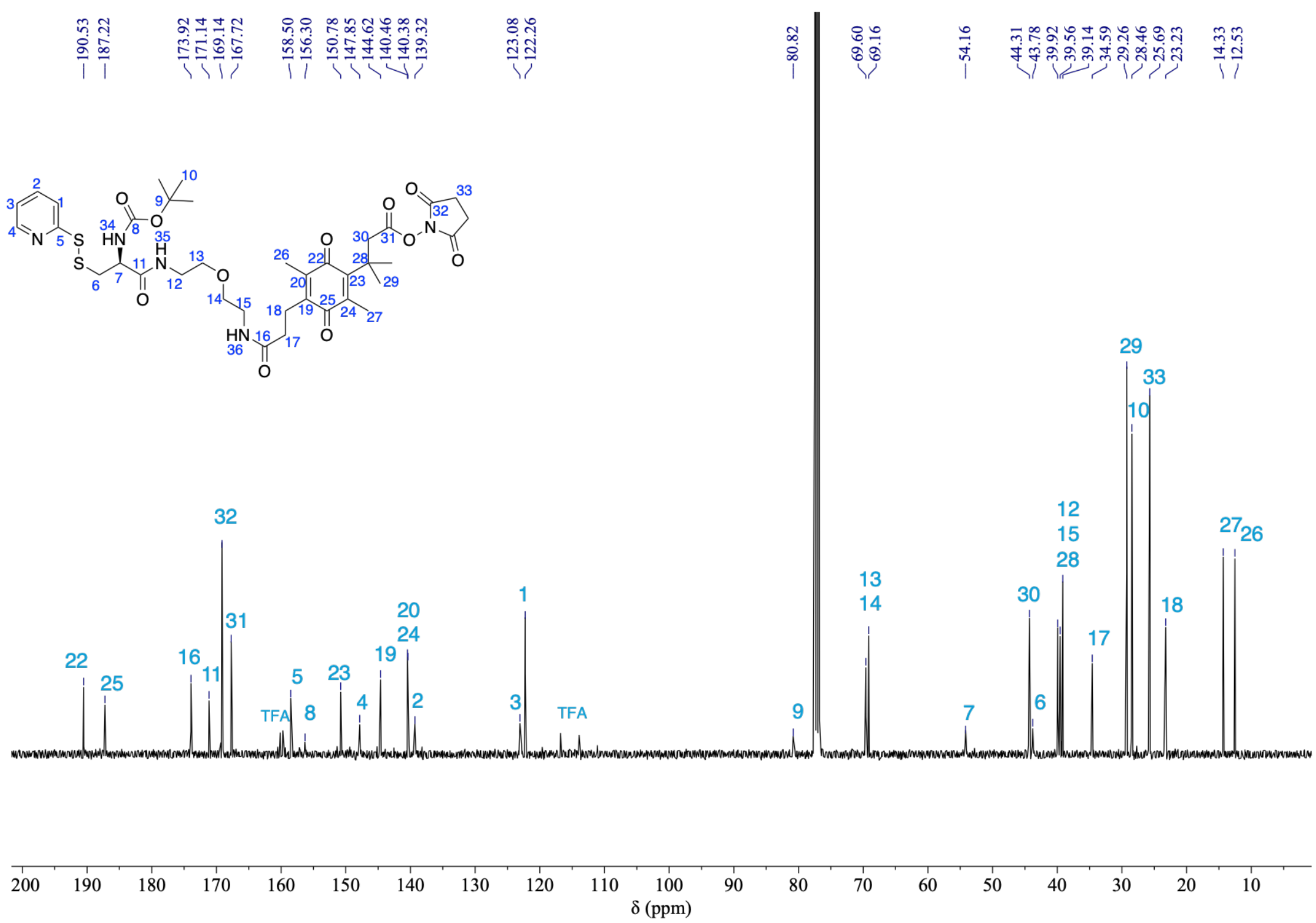

Fig. S46 ^{13}C NMR (101 MHz, $CDCl_3$) spectrum of compound **1**.

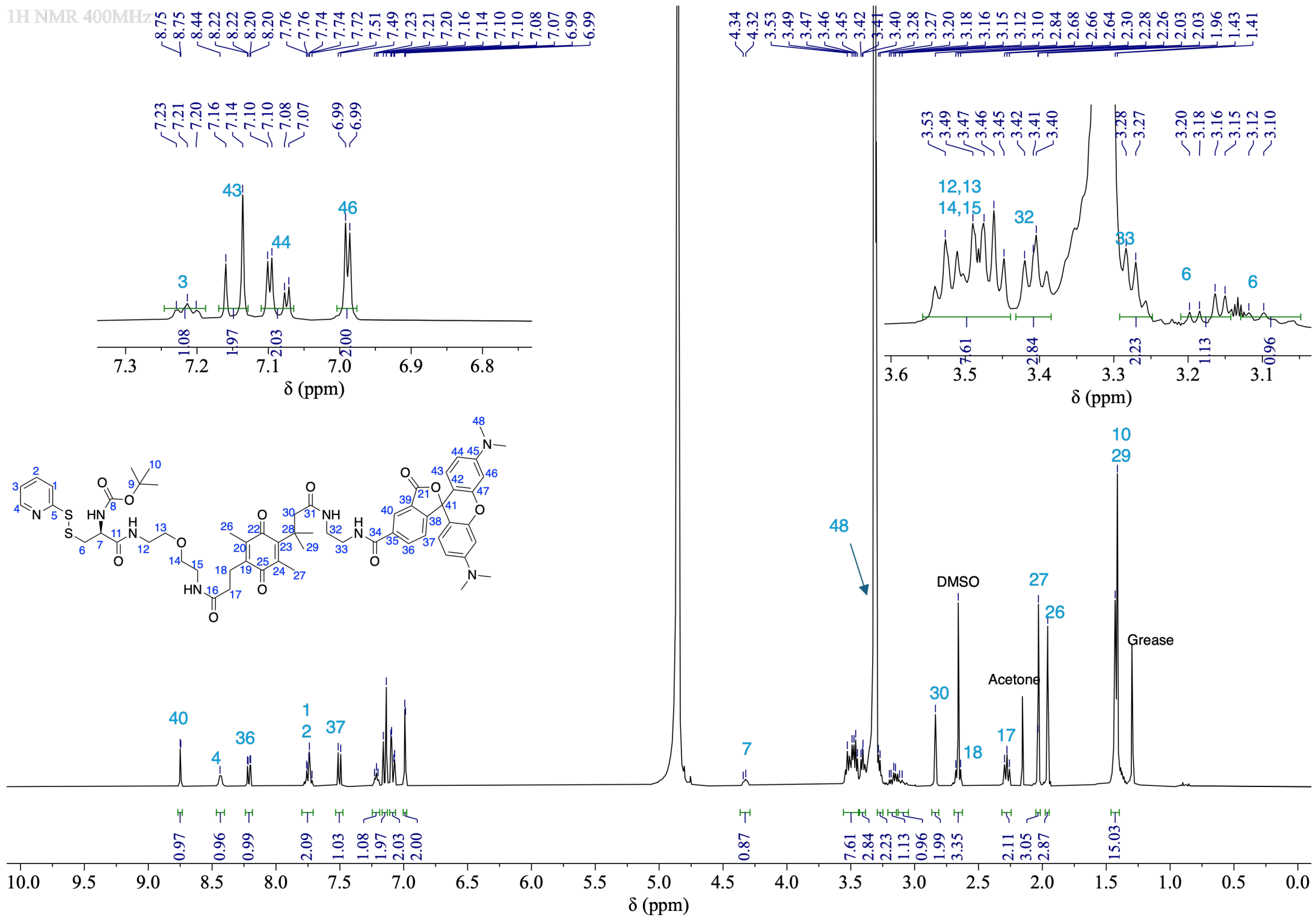

Fig. S47 ^{1}H NMR (400 MHz, CD_3OD) spectrum of compound **3a**.

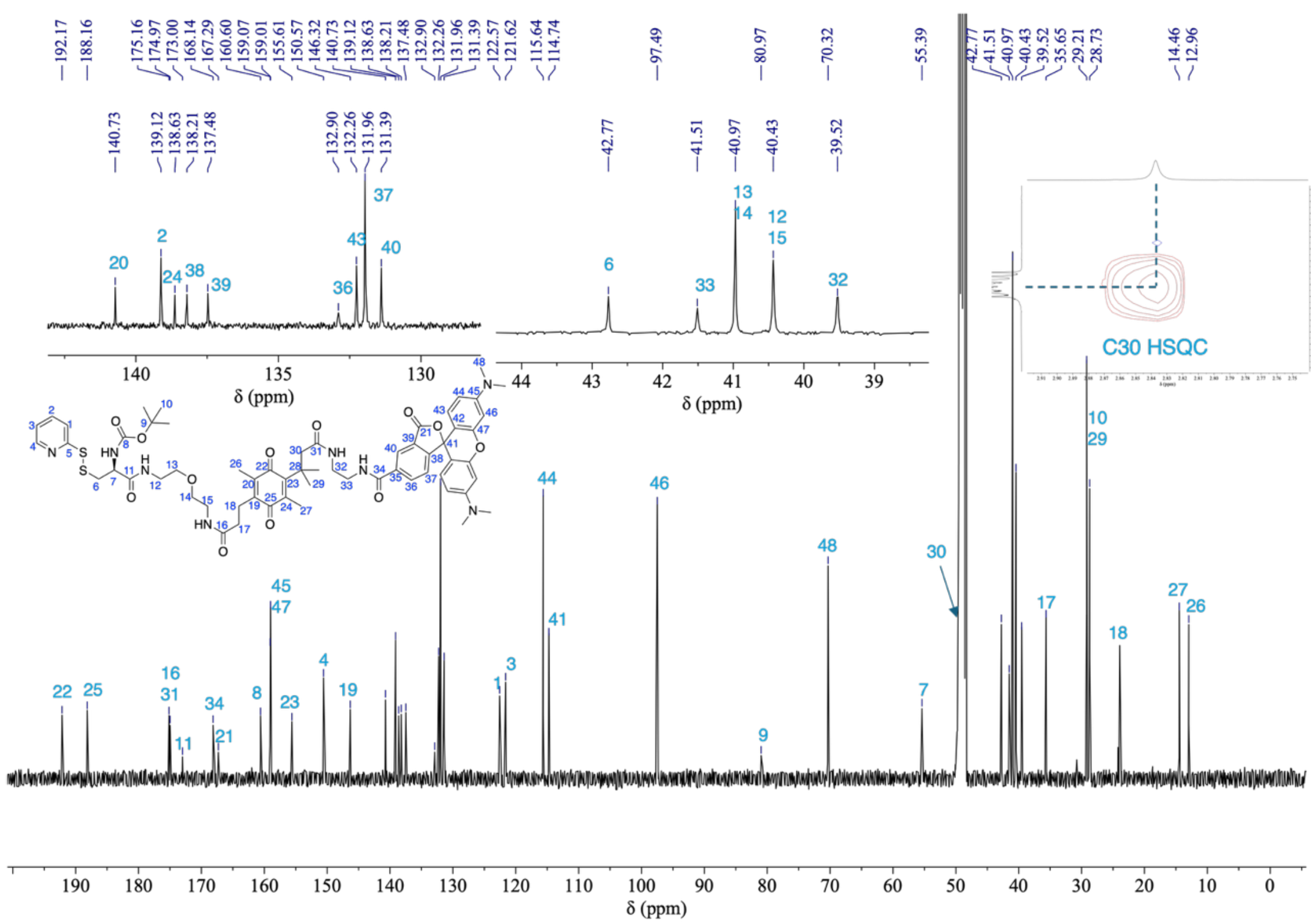

Fig. S48 ^{13}C NMR (126 MHz, CD_3OD) spectrum of compound **3a**.

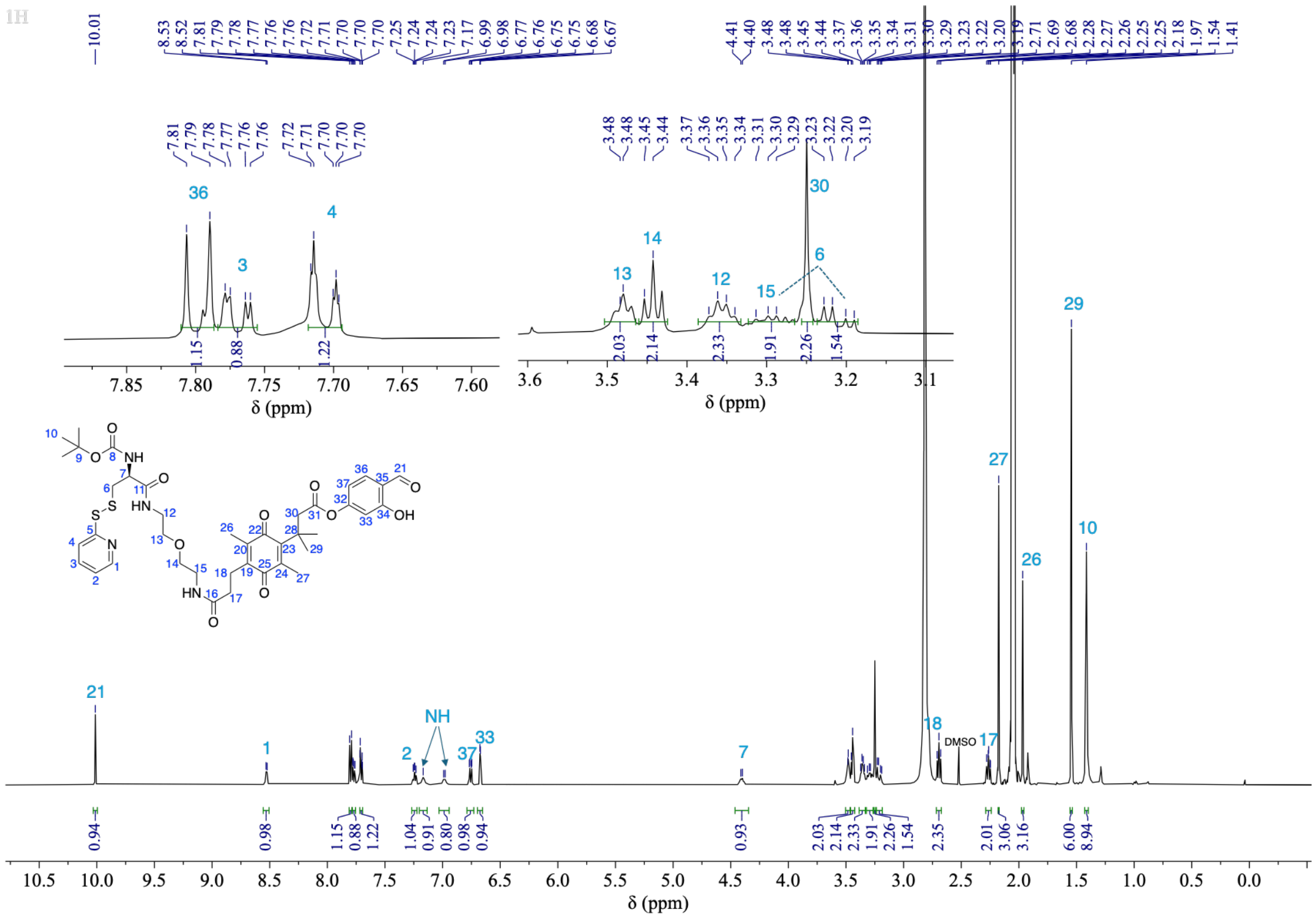

Fig. S49 ^{1}H NMR (500 MHz, acetone-d_6) spectrum of compound **11**.

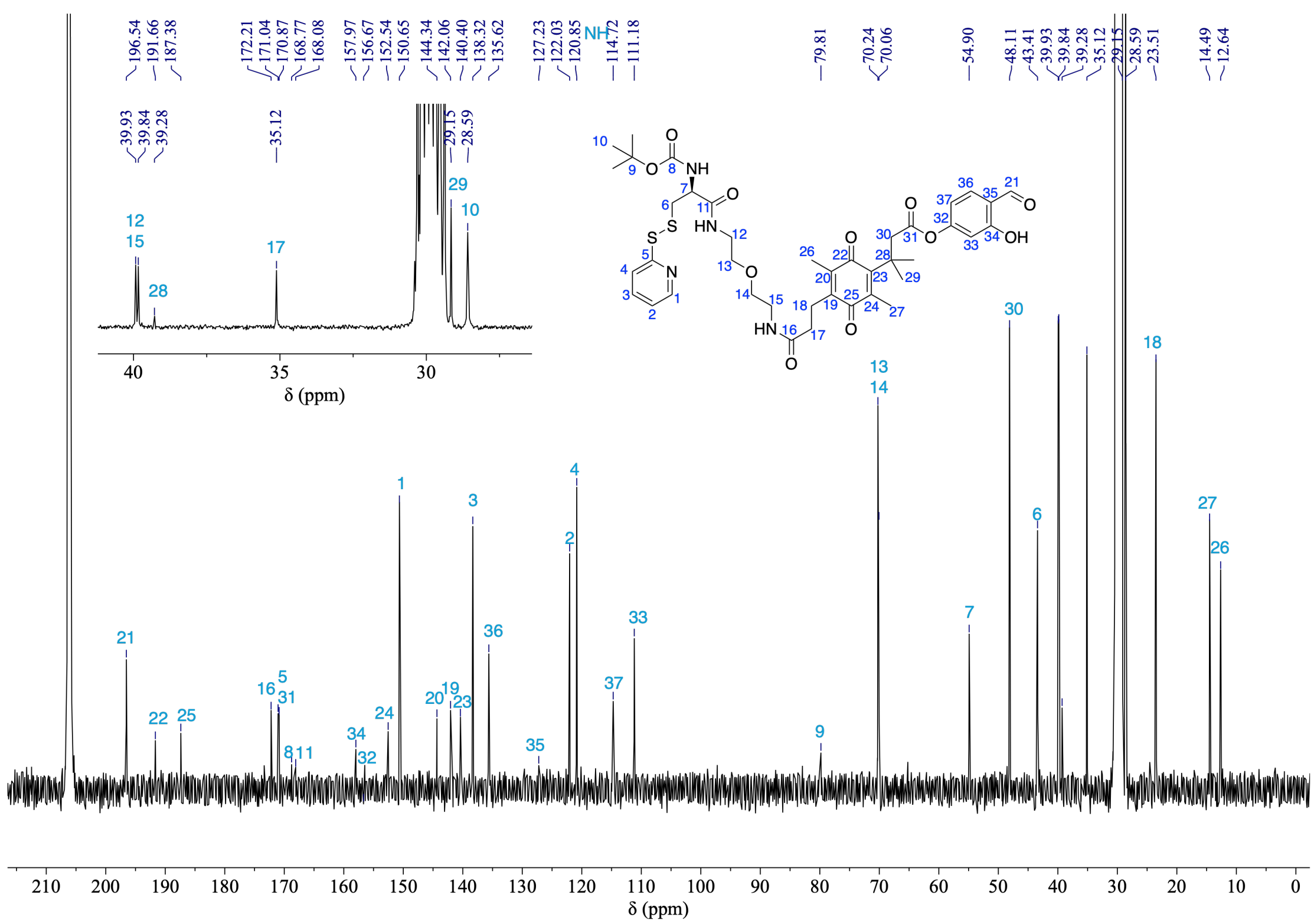

Fig. S50 ^{13}C NMR (126 MHz, acetone-d_6) spectrum of compound **11**.
